# Human neuronal networks on micro-electrode arrays as a tool to assess genotype-phenotype correlation in *CACNA1A*-related disorders

**DOI:** 10.1016/j.stemcr.2025.102783

**Published:** 2026-01-22

**Authors:** Marina P. Hommersom, Sofía Puvogel, Nicky Scheefhals, Eleonora Carpentiero, Marga Bouma, Ellen van Beusekom, Lieke Dillen, Bart P.C. van de Warrenburg, Nael Nadif Kasri, Hans van Bokhoven

**Affiliations:** 1 Department of Human Genetics, Radboud University Medical Center, Donders Institute for Brain, Cognition, and Behaviour, Nijmegen 6500 HB, the Netherlands; 2 Department of Neurology, Radboud University Medical Center, Donders Institute for Brain, Cognition, and Behaviour, Nijmegen 6500 HB, the Netherlands; 3 Department of Cognitive Neurosciences, Radboud University Medical Center, Donders Institute for Brain, Cognition, and Behaviour, Nijmegen 6500 HB, the Netherlands

**Keywords:** *CACNA1A*, ataxia, epilepsy, migraine, micro-electrode arrays, neuronal networks, induced pluripotent stem cells, human disease modelling, variant classification, GABAergic neurons

## Abstract

*CACNA1A*-related disorders constitute a diverse group of neurological conditions, including ataxia, migraine, and epilepsy. Despite extensive genetic studies, clear genotype-phenotype correlations remain elusive. Moreover, next-generation sequencing has identified many variants of uncertain significance (VUS). Here, we leveraged patient-derived and CRISPR-Cas9-engineered human neuronal networks to explore relationships between *CACNA1A* variants and neurophysiological activity. *CACNA1A* haploinsufficiency induced subtle alterations in glutamatergic network activity, whereas missense variants had a more pronounced effect on overall network function. Network fingerprints were most affected from patients where ataxia co-occurred with migraine or epilepsy. Furthermore, we analyzed the impact of CRISPR-Cas9-induced VUS on network developmental trajectories. Although functional changes could not be directly linked to clinical phenotypes, all tested variants induced measurable alterations in neuronal network function, supporting their classification as likely pathogenic. These findings highlight the potential of human neuronal networks as a translational model for evaluating *CACNA1A* variant effects and improving clinical variant interpretation.

## Highlights

• *CACNA1A* patient-derived human neuronal networks showed variant-specific alterations
• A gain-of-function variant linked to epilepsy caused fragmented synchronization
• This variant also led to an early increased inhibitory drive in GABAergic neurons
• Human neuronal networks supported classification of three VUS as likely pathogenic

## Graphical abstract

**Figure:**
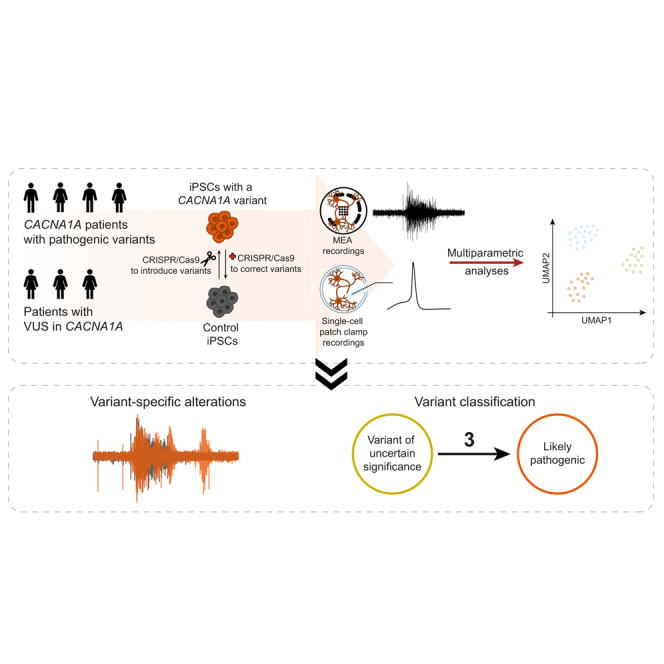

## Teaser

Van Bokhoven and colleagues demonstrate functional profiling of *CACNA1A* patient-derived neuronal networks, including some with CRISPR-Cas9-corrected/mimicked variants. They uncovered variant-specific alterations and provided support for classification of three variants of uncertain significance (VUS). Findings highlighted both the strengths and limitations of iPSC-based neuronal modelling, with significant implications for future endeavours to improve variant classification and understanding of *CACNA1A*-related disorders.

## Introduction

*CACNA1A*-related disorders encompass a heterogeneous group of neurological conditions, primarily characterized by episodic or progressive ataxia, hemiplegic migraine, and various forms of epilepsy (Indelicato and Boesch 2021; Hommersom et al., 2022). The gene *CACNA1A* encodes the pore-forming subunit of the P/Q-type voltage-gated calcium channel, Ca_V_2.1, which plays a critical role in neuronal function. Ca_V_2.1 is predominantly located at presynaptic terminals, where it regulates neurotransmitter release (Llinás et al., 1989; Turner et al., 1992; Uchitel et al., 1992; Luebke et al., 1993; Takahashi and Momiyama 1993; Wheeler et al., 1994; Lübbert et al., 2019), but it is also present in dendrites and soma contributing to the control of neuronal excitability (Yu et al., 2010). The channel’s function is fine-tuned by its position in the plasma membrane, its kinetic properties, and voltage dependence, which are influenced by the intrinsic properties of specific splice isoforms and their interactions with various auxiliary subunits (Soong et al., 2002; Chaudhuri et al., 2004; Davies et al., 2007; Buraei and Yang 2010; Thalhammer et al., 2017). Given this complexity, it is unsurprising that *CACNA1A* variants can have wide-ranging molecular, cellular, and systemic effects.

Functional analysis of overexpressed *CACNA1A* variants in HEK293T cells has provided valuable insights into specific effects of genetic variants on Ca_V_2.1 channel function (Guida et al., 2001; Cuenca-León et al., 2009; Garza-López, et al. 2012, 2013; Condliffe et al., 2013; Ohmori et al., 2013; Bahamonde et al., 2016; Huang et al., 2019; Jiang et al., 2019; Gandini et al., 2021). However, these models lack the physiological complexity and cellular context of the human brain. They do not capture the influence of genetic background, alternative splicing, or auxiliary subunit interaction, limiting their relevance for understanding the full spectrum of *CACNA1A-*related phenotypes.

In contrast, human-derived neuronal networks offer a distinct advantage for modelling neurological disorders, as they maintain both the human genetic context and physiological relevance. Another direct advantage of using patient-derived cells is that they allow to make direct connection between experimental data and a specific constellation of clinical features. Advances in CRISPR-Cas9 gene-editing techniques enable us to precisely investigate the effects of specific variants by utilizing isogenic cell lines. Furthermore, electrophysiological techniques allow for detailed examination of neuronal (network) activity, shedding light on how human variants affect single-cell or network-level output. Previously, we have demonstrated that control networks exhibit robust and highly similar functional properties (Mossink et al., 2021) and that disturbances in neuronal network activity are characteristic for various neurodevelopmental disorders (Frega et al., 2019; Klein Gunnewiek et al., 2020; Mossink et al., 2021; Linda et al., 2022; Wang et al., 2022; van Hugte et al., 2023). In our recent work, we demonstrated that *CACNA1A* haploinsufficiency in induced pluripotent stem cell (iPSC)-derived neurons results in altered network synchronization that is accompanied by reduced synaptic function and increased intrinsic excitability (Hommersom et al., 2024). However, a key limitation of this study was that we relied solely on CRISPR-Cas9-engineered control iPSCs that prevented us from linking our findings to specific clinical manifestations.

To address this issue, we here use multiple patient iPSC-derived neuronal networks consisting of glutamatergic only or glutamatergic and GABAergic neurons to characterize alterations in neurophysiological activity. We measured their electrical activity, both through patch clamp recordings to study single-cell level activity and micro-electrode arrays (MEAs) to study neuronal network-level activity. We hypothesized that neuronal network functioning could discriminate the different iPSC-derived networks based on the different variant types and/or different phenotypes. We show that specific parameters could be correlated to Ca_V_2.1 gain-of-function with ataxia and epilepsy as a clinical phenotype, which were rescued by Ca_V_2 inhibitors. Moreover, we demonstrate an increased inhibitory drive in GABAergic neurons carrying this gain-of-function variant. Lastly, we explored the classification of variants of uncertain significance (VUS) by leveraging the neuronal network phenotypes, hereafter referred to as network fingerprints.

## Results

### *CACNA1A* patient-derived glutamatergic neuronal networks show variant type-specific network fingerprints

**Figure 1:**
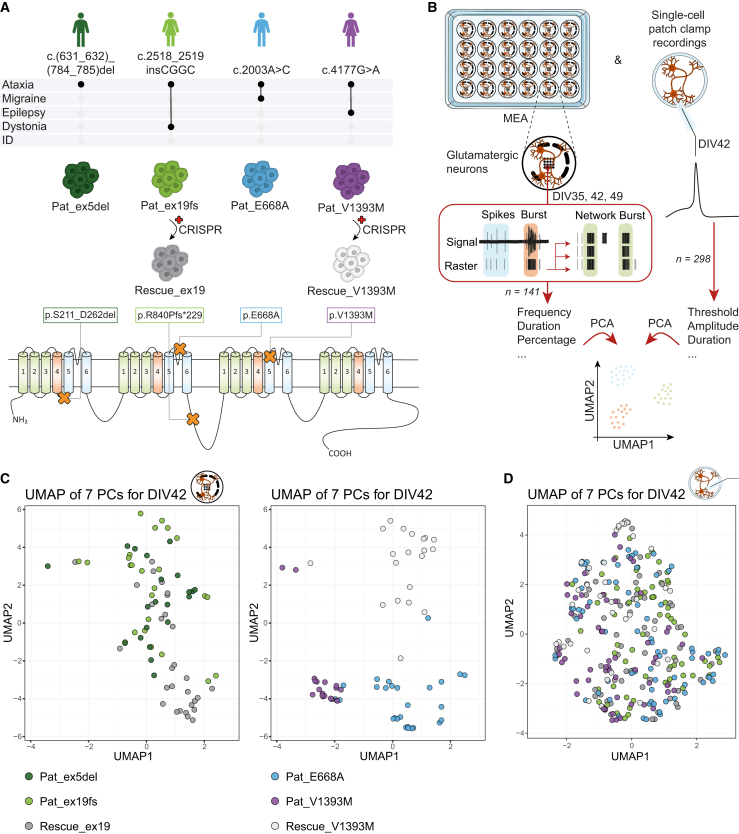
Clustering of *CACNA1A* patient-derived glutamatergic neuronal networks based on their electrophysiological activity over late development (A) Schematic overview of the clinical phenotypes of *CACNA1A* patients included in this study with their genetic variants (NM_00127221). Two patient-derived induced pluripotent stem cell (iPSC) lines were genetically edited with CRISPR-Cas9 to generate isogenic controls. Genetic variants are also represented as orange crosses in a schematic representation of Cav2.1. Transmembrane domains are shown in green, voltage-sensitive domains in red, and pore-forming domains in blue. (B) Schematic overview of neuronal differentiation toward recording network activity on micro-electrode arrays (MEAs) at days *in vitro* (DIV)35, 42, and 49 and recording single-cell electrophysiological activity at DIV42. Principal component analysis (PCA) was performed on parameters extracted from these recordings, and seven principal components (PCs) were plotted in uniform manifold approximation and projection (UMAP) space. (C) UMAP analysis on seven PCs obtained from network activity parameters at DIV42 for all iPSC-derived networks indicated in (A). *n =* 21/3 for Pat_ex5del, *n =* 25/4 for Pat_ex19fs, *n =* 29/4 for Rescue_ex19, *n =* 27/4 for Pat_E668A, *n =* 19/3 for Pat_V1393M, and *n =* 20/2 for Rescue_V1393M. (D) UMAP analysis on seven PCs obtained from single-cell intrinsic properties at DIV42 for all iPSC-derived indicated in (A), except for Pat_ex5del. *n =* 68/8 for Pat_ex19fs, *n =* 63/7 for Rescue_ex19, *n =* 73/5 for Pat_E668A, *n =* 57/5 for Pat_V1393M, and *n =* 41/2 for Rescue_V1393M. See also Figure S1.

In order to characterize the neuronal activity of different *CACNA1A* patient-derived neuronal networks, we used iPSC lines of four different patients with diverse clinical phenotypes and genetic variants (Figure 1A). All patients showed episodic or chronic ataxia, either isolated or combined with dystonia, migraine, or epilepsy. The patient-derived iPSC lines harboured different genetic variants, including an exon deletion (Pat_ex5del), a frameshift variant (Pat_ex19fs), and two missense variants, of which one was predicted loss-of-function (Pat_E668A) and one gain-of-function (Pat_V1393M) (Jiang et al., 2019). We generated two isogenic control lines, by rescuing the frameshift variant of Pat_ex19fs (Rescue_ex19) and the missense variant of Pat_V1393M (Rescue_V1393M). We then differentiated these iPSC lines toward glutamatergic neurons via doxycycline-induced *Ngn2* overexpression and recorded their neuronal network activity using MEAs over late development, when all networks showed synchronization in the form of network bursts (Figure 1B). For all networks, except for Pat_ex5del, we also recorded their intrinsic electrophysiological activity through single-cell patch-clamp recordings (Figure 1B). To get a first impression on how the different neuronal network activities would compare to each other, we performed a principal component analysis (PCA) on all available parameters. For 39 MEA parameters recorded over 3 days *in vitro* (DIVs), we plotted the results of seven principal components (PCs) in a uniform manifold approximation and projection (UMAP) space (Figures 1C and S1A). Patient lines harbouring missense variants that are associated with ataxia combined with migraine and epilepsy, clustered further apart from the (isogenic) control lines than patient lines with full loss-of-function alleles that were associated with ataxia and dystonia. For eleven single-cell electrophysiological properties recorded at DIV42, we applied the same approach but observed no clustering (Figure 1D). This suggests that this multiparametric approach cannot be used to discriminate different patient-derived networks with their single-cell intrinsic electrophysiological activity, whereas changes were uncovered between the different networks using single parameters (Figure S1B).

**Figure 2:**
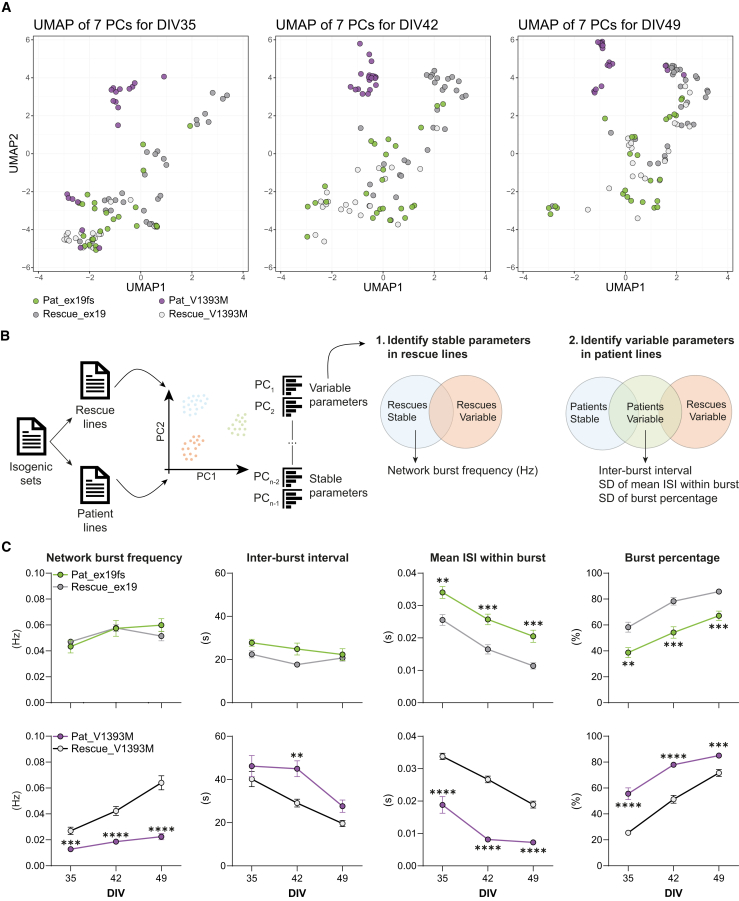
Identification of variant- or phenotype-specific developmental changes in network activity parameters (A) Uniform manifold approximation and projection (UMAP) analysis on seven principal components (PCs) obtained from network activity parameters at days *in vitro* (DIV)35, 42, and 49 for the isogenic iPSC-derived networks indicated in Figure 1A. (B) Schematic overview of analysis steps taken to isolate network activity parameters. (C) Network activity parameters over DIV35, 42, and 49 for the patient and isogenic rescue lines, including network burst frequency, inter-burst interval, mean inter-spike interval (ISI) within burst, and burst percentage. *n = 25/4* for Pat_ex19fs, *n =* 29/4 for Rescue_ex19, *n =* 19/3 for Pat_V1393M, and *n =* 20/2 for Rescue_V1393M. Data are represented as mean ± standard error of the mean (SEM). ^∗∗^*p* < 0.01, ^∗∗∗^*p* < 0.001, ^∗∗∗∗^*p* < 0.0001, two-way ANOVA with mixed-effects model if there are missing values, followed by Šídák’s test for multiple comparisons. See also Figure S1; Table S1.

To narrow down on the parameters that could describe the different variant types (*null* variants vs. missense variants), we focused on the isogenic pairs (Figure 2A). We first stratified the dataset into patient-derived networks and isogenic control (rescue) networks (Figure 2B) and performed PCA on these separate datasets. By selecting ten parameters contributing the most to each PC, we identified those responsible for the greatest variance, i.e., variable parameters (belonging to PC1 and PC2), as well as those contributing the least variance, i.e., stable parameters (belonging to the last two PCs). To minimize the inclusion of extraneous variables, we applied two filtering strategies. First, we identified parameters that remained stable across both rescue networks (Figure 2B). Next, by overlaying the 20 variable parameters with the 20 stable parameters, we extracted those that contributed exclusively to the last two PCs and not to the first two PCs (Table S1). This approach yielded a key parameter: network burst frequency, defined by the number of network bursts over a 5-min period. Indeed, the network burst frequency of both rescue lines fell within similar ranges (Figure 2C). While we did not observe changes in the network burst frequency for the frameshift variant, the neurons carrying the gain-of-function missense variant (Pat_V1393M) consistently showed a reduced network burst frequency throughout development (Figure 2C).

To further refine our analysis, we focused on parameters that varied in patient networks but not in rescue networks (Figure 2B). This approach identified three key parameters: inter-burst interval (IBI), the standard deviation (SD) of the mean inter-spike interval (ISI) within bursts, and the SD of burst percentage. In isogenic control networks, the IBI was either stable or decreased over development (Figure 2C). While Pat_ex19fs did not show significant differences, Pat_V1393M displayed longer IBI at DIV42. For both the mean ISI within burst, reflecting the compactness of spikes within a burst, and the burst percentage, representing the percentage of spikes that occur within bursts, Pat_ex19fs and Pat_V1393M showed opposite trends compared to their isogenic controls (Figure 2C). Pat_ex19fs exhibited a higher mean ISI within burst and a lower burst percentage than Rescue_ex19, whereas Pat_V1393M showed the opposite trend compared to Rescue_V1393M. Notably, these parameters also varied within the rescue lines. This suggests that rescuing a variant does not necessarily restore all network parameters to control levels.

We then compared both full loss-of-function variants and both missense variants to each other and the isogenic controls (Figure S1C). Interestingly, Pat_ex5del and Pat_ex19fs exhibited very similar network features, and both showed an increased mean ISI within burst and lower burst percentage compared to Rescue_ex19. In contrast, Pat_V1393M and Pat_E668A showed more distinct features, particularly in network burst frequency trajectories and the IBI, yet demonstrated comparable mean ISI within burst and burst percentage. This suggests that these two parameters are particularly informative for assessing variant-specific effects relative to the corresponding isogenic control.

In summary, our results suggest that full loss-of-function alleles induce subtle alterations in developmental network activity, whereas missense variants have a more pronounced impact on developmental trajectories. Notably, while network fingerprints remain relatively similar in cases of dystonia co-occurring with ataxia (Pat_ex19fs), the presence of migraine or epilepsy seems to be associated with significant shifts in the network fingerprint (Pat_E668A and Pat_V1393M).

### Cav2.1 gain-of-function leads to fragmented synchronization in glutamatergic neuronal networks

**Figure 3:**
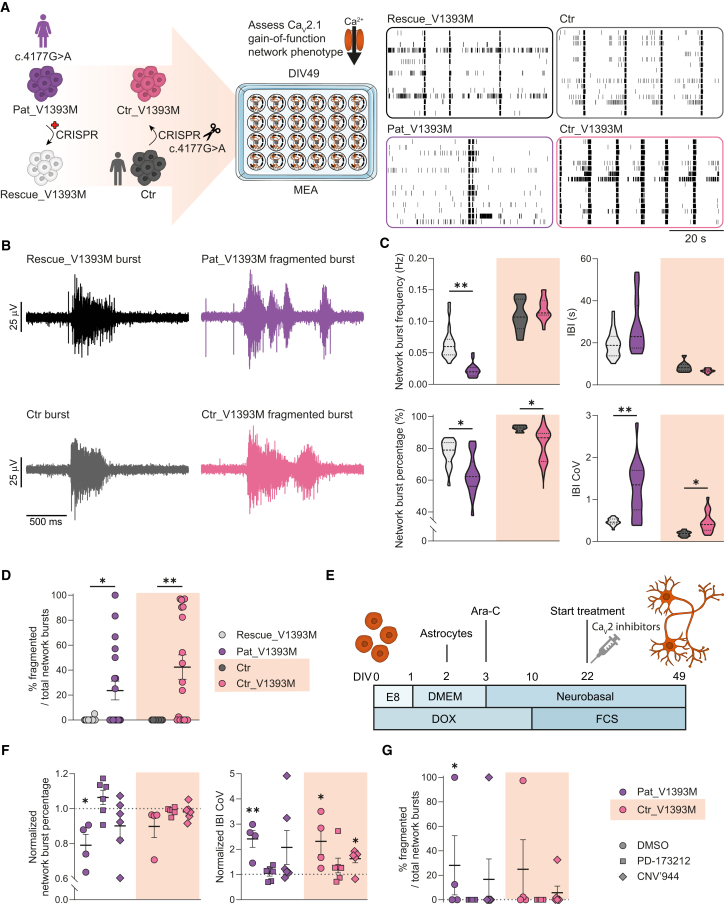
Specific network parameters of p.(V1393M) neuronal networks are rescued by Cav2 inhibitors (A) Schematic overview of the two isogenic p.(V1393M) pairs that were differentiated toward glutamatergic neurons and recorded on micro-electrode array (MEA) including representative rasterplots (60 s) of network activity of the indicated neuronal networks at days *in vitro* (DIV)49. (B) Representative electrode bursts (2 s) of Rescue_V1393M and Ctr neuronal networks showing a burst, and Pat_V1393M and Ctr_V1393M neuronal networks showing fragmented synchronization. (C) Quantification of network activity parameters at DIV49 for isogenic control and p.(V1393M) lines, including network burst frequency, inter-burst interval (IBI), network burst percentage, and IBI coefficient of variation (CoV). *n =* 20/3 for Rescue_V1393M, *n =* 19/3 for Pat_V1393M, *n =* 9/3 for Ctr, and *n =* 19/3 for Ctr_V1393M. Dashed line represents the median, dotted line represents the quartiles. ^∗^*p* < 0.05, ^∗∗^*p* < 0.01, Kruskal-Wallis test with Dunn’s test for multiple comparisons. (D) Quantification of the percentage fragmented over total network bursts. *n =* 20/3 for Rescue_V1393M, *n =* 19/3 for Pat_V1393M, *n =* 9/3 for Ctr, and *n =* 19/3 for Ctr_V1393M. Data are represented as mean ± standard error of the mean (SEM). ^∗^*p* < 0.05, ^∗∗^*p* < 0.01, Kruskal-Wallis test with Dunn’s test for multiple comparisons. (E) Schematic overview of treatment strategy of p.(V1393M) networks with Cav2 inhibitors. Dimethyl sulfoxide (DMSO) was used as a vehicle. (F) Quantification of normalized network parameters including network burst percentage and IBI CoV upon treatment with vehicle (DMSO; 0.05%) or Cav2 inhibitors, PD-173212 (500 nM) or CNV’944 (30 μM). Parameters were normalized against the respective control values. *n =* 4/2 for Pat_V1393M + DMSO, *n =* 6/2 for Pat_V1393M + PD-173212, *n =* 6/2 for Pat_V1393M + CNV’944, *n =* 4/2 for Ctr_V1393M + DMSO, *n =* 6/2 for Ctr_V1393M + PD-173212, and *n =* 6/2 for Ctr_V1393M + CNV’944. Data are represented as mean ± SEM. ^∗^*p* < 0.05, *^∗∗^p* < 0.01, Kruskal-Wallis test with Dunn’s test for multiple comparisons. (G) Quantification of the percentage fragmented over total network bursts upon treatment with vehicle (DMSO; 0.05%) or Cav2 inhibitors, PD-173212 (500 nM) or CNV’944 (30 μM). *n =* 4/2 for Pat_V1393M + DMSO, *n =* 6/2 for Pat_V1393M + PD-173212, *n =* 6/2 for Pat_V1393M + CNV’944, *n =* 4/2 for Ctr_V1393M + DMSO, *n =* 6/2 for Ctr_V1393M + PD-173212, and *n =* 6/2 for Ctr_V1393M + CNV’944. Data represented as mean ± SEM. ^∗^*p* < 0.05, Kruskal-Wallis test with Dunn’s test for multiple comparisons. See also Table S2.

We previously characterized the network fingerprint of *CACNA1A* haploinsufficient networks (Hommersom et al., 2024). To further investigate the effects of a missense variant, we introduced the predicted gain-of-function p.(V1393M) variant into a control cell line. We focused on DIV49 (Figure 3A), the time point at which the most pronounced differences between isogenic pairs were observed (Figure 2C). By applying the same PCA-based approaches as before (Table S2), we found that p.(V1393M) networks (Pat_V1393M and Ctr_V1393M) exhibited fragmented bursts, a feature absent in control networks (control and Rescue_V1393M) (Figures 3A, 3B, and 3D). This is interesting as fragmented bursts have been linked to increased calcium availability in the presynapse (Pradeepan et al., 2024), and we previously classified fragmented bursts as seizurogenic activity in control networks upon treatment with proconvulsive compounds (van Hugte et al., 2023). As in previous analyses, we found that the network burst frequency remained stable across all conditions, whereas network burst percentage, IBI, and IBI coefficient of variation (CoV) were variable in p.(V1393M) but not in the control networks. Compared to their respective isogenic controls, Pat_V1393M networks showed a decrease in network burst frequency (Figure 3C), suggesting that human genetic context influences the expressed network fingerprint. While IBI itself was not significantly altered, both IBI CoV and network burst percentage were increased in Pat_V1393M and Ctr_V1393M networks compared to their isogenic controls, indicating that these parameters are specifically modulated by the p.(V1393M) variant.

Since the p.(V1393M) variant has been shown to result in gain-of-function of Ca_V_2.1 (Jiang et al., 2019), we explored whether these network alterations could be rescued by treating the networks with Ca_V_2 inhibitors, PD-173212 (Okada et al., 2021), and CNV’944 (Figure 3E). PD-173212 treatment restored both IBI CoV and network burst percentage to control levels (Figure 3F), while also eliminating fragmented bursts in p.(V1393M) neurons (Figure 3G). CNV’944 treatment reduced the percentage of fragmented bursts (Figure 3G), though it did not fully restore IBI CoV in Ctr_V1393M neurons (Figure 3F).

Taken together, these findings demonstrate that network burst fragmentation, IBI CoV, and network burst percentage are specifically modulated by the p.(V1393M) missense variant in *CACNA1A*, independent of the genetic background. Furthermore, these parameters may be linked to clinical phenotypes such as chronic ataxia and epilepsy (van Hugte et al., 2023).

### Cav2.1 gain-of-function leads to an early increased inhibitory drive in GABAergic neurons

**Figure 4:**
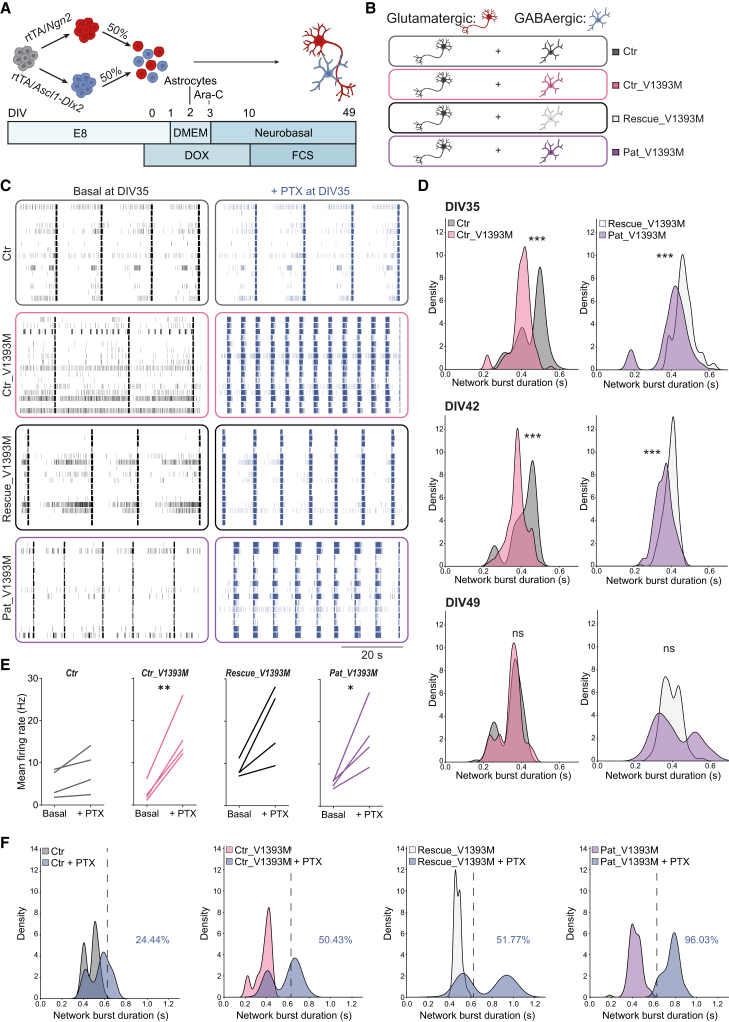
Cav2.1 gain-of-function leads to an early increased inhibitory drive in iPSC-derived GABAergic neurons (A) Schematic representation of the differentiation protocol from iPSCs to glutamatergic/GABAergic co-cultures. (B) Schematic overview of co-cultured conditions used in this study. (C) Representative rasterplots (60 s) of network activity of the indicated neuronal networks at days *in vitro* (DIV)35 before (basal) and after 5 min treatment with 100 μM picrotoxin (PTX). (D) Density plots of network burst durations of Ctr glutamatergic neurons cultured with Ctr, Ctr_V1393M, Rescue_V1393M, or Patient_V1393M GABAergic neurons at DIV35, 42, and 49. *n* = 12/2 for Ctr, *n* = 12/2 for Ctr_V1393M, *n* = 12/2 for Rescue_V1393M, and *n* = 12/2 for Pat_V1393M. ^∗∗∗^*p* < 0.001, Wilcoxon rank-sum test with continuity correction. (E) Quantification of mean firing rate at DIV35 at baseline and after 5 min treatment with 100 μM PTX. *n =* 4/2 for Ctr, *n =* 4/2 for Ctr_V1393M, *n =* 4/2 for Rescue_V1393M, and *n =* 4/2 for Pat_V1393M. ^∗^*p* < 0.05, *^∗∗^p* < 0.01, paired *t* test. (F) Density plots of network burst durations of Ctr glutamatergic neurons cultured with Ctr, Ctr_V1393M, Rescue_V1393M, or Patient_V1393M GABAergic neurons at baseline and after 5 min treatment with 100 μM PTX at DIV35. *n* = 4/2 for Ctr, *n* = 4/2 for Ctr_V1393M, *n* = 4/2 for Rescue_V1393M, and *n* = 4/2 for Pat_V1393M. The percentage indicates the percentage of events after treatment with PTX exceeding 0.625 s, the threshold above which fewer than 2.5% of network burst durations occurred under basal conditions across all groups. See also Figure S2.

CACNA1A is not only essential for glutamatergic neurotransmission but also plays a prominent role in GABAergic neurons (Rossignol et al., 2013; Lupien-Meilleur et al., 2021; Singh et al., 2023), potentially contributing to the coordination between excitation (E) and inhibition (I). To investigate whether *CACNA1A* variants in GABAergic neurons contribute to shifts in E/I coordination, we differentiated Ctr, Ctr_V1393M, Rescue_V1393M, and Pat_V1393M toward GABAergic neurons, via overexpression of *Ascl1* and *Dlx2* (Figure 4A). This recently published protocol efficiently yields pure GABAergic neurons (van Voorst et al., 2025). We then co-cultured these GABAergic neurons with glutamatergic neurons at a 1:1 ratio, resulting in functional E/I networks with an approximate 60:40 glutamatergic-to-GABAergic composition, determined by immunocytochemistry and flow cytometry (Figures S2A–S2C). Within these co-cultures, we consistently observed the formation of functional inhibitory synapses, as evidenced by the colocalization of presynaptic VGAT and postsynaptic gephyrin (Figures S2D and S2E).

To specifically assess the effect of the p.(V1393M) variant on GABAergic neuronal output while maintaining a consistent glutamatergic population, we co-cultured Ctr glutamatergic neurons with GABAergic neurons from Ctr, Ctr_V1393M, Rescue_V1393M, and Pat_V1393M lines (Figure 4B). We then recorded spontaneous network activity on MEAs at DIV35, DIV42, and DIV49 (Figures 4C and 4D). To better capture the heterogeneity in network burst durations and avoid over-reliance on mean values, we analyzed the full distribution of individual network burst durations (Figure 4D). Our rationale was that not all neurons undergo the GABA shift simultaneously, and this cellular heterogeneity can influence network-level readouts in ways that average-based comparisons may obscure. Consistent with our previous findings (Mossink et al., 2022), network burst duration decreased over development of the control networks (Figure 4D), a characteristic parameter for functional inhibition by GABAergic neurons (Mossink et al., 2022; Wang et al., 2023). At DIV35 and DIV42, we observed a clear shift in the distribution toward shorter network burst duration with p.(V1393M) GABAergic neurons (Figure 4D), an effect that disappeared by DIV49. This suggests that p.(V1393M) GABAergic neurons exert an increased inhibitory drive during early development. To further confirm this, we treated the networks with picrotoxin (PTX) at DIV35 to assess how the network responded to acute removal of inhibitory control (Figure 4C) (Mossink et al., 2022). As expected, PTX treatment did not alter the mean firing rate in control networks at DIV35 (Figure 4E), a developmental stage at which the hyperpolarizing shift of the GABA reversal potential has not yet occurred in all neurons of the network (Mossink et al., 2022). However, in both p.(V1393M) networks, the mean firing rate increased upon treatment with PTX (Figure 4E). To assess responses to PTX more precisely, we quantified the proportion of prolonged network bursts exceeding 0.625 s, a threshold above which fewer than 2.5% of events occurred under basal conditions across all groups (Figure 4F). Following PTX treatment, a higher proportion of long-duration network bursts was observed in p.(V1393M) networks compared to their isogenic controls. These findings support the conclusion that the p.(V1393M) variant enhances GABAergic output during early network development.

Taken together, these results suggest that the *CACNA1A* p.(V1393M) variant induces a shift in E/I coordination, characterized by increased inhibition during early neuronal network development. It is worth highlighting that the patient carrying the p.(V1393M) missense variant exhibits the earliest and most pronounced symptoms among the individuals we investigated. This indicates that MEA-based neuronal networks provide a powerful platform for establishing genotype-phenotype correlations and assessing disease-related network dysfunction.

### VUS in *CACNA1A* alter neuronal network activity but cannot be linked to a clinical phenotype when mimicked by CRISPR-Cas9

**Figure 5:**
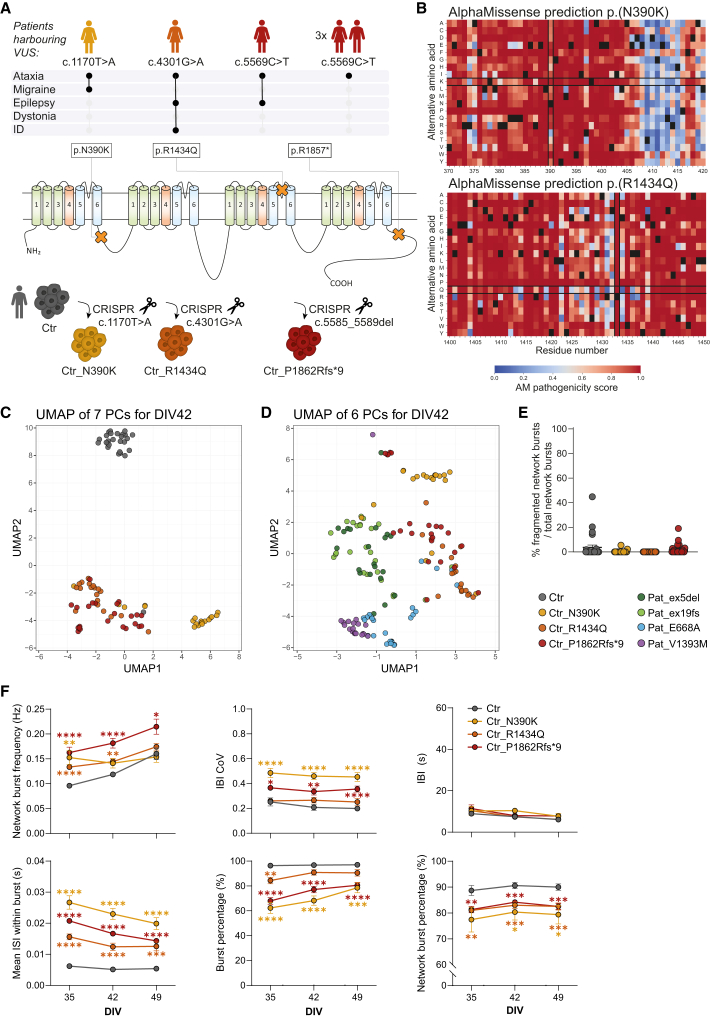
Clustering of glutamatergic neuronal networks carrying introduced *CACNA1A* variants of uncertain significance based on their network activity over late development (A) Schematic overview of the clinical phenotype of *CACNA1A* patients resembled in this study with their genetic variants (NM_00127221). These variants were mimicked in a control induced pluripotent stem cell (iPSC) line with CRISPR-Cas9 to generate isogenic lines. Genetic variants are also represented as orange crosses in a schematic representation of Cav2.1. Transmembrane domains are shown in green, voltage-sensitive domains in red, and pore-forming domains in blue. (B) Pathogenicity scores predicted by AlphaMissense for missense variants. (C and D) Uniform manifold approximation and projection (UMAP) analysis on seven principal components (PCs) obtained from network activity parameters at days *in vitro* (DIV)42 for all iPSC-derived networks carrying variants of uncertain significance (VUS) compared to the isogenic control (C) and compared to all patient-derived networks indicated in Figure 1A (D). *n =* 21/3 for Pat_ex5del, *n =* 25/4 for Pat_ex19fs, *n =* 27/4 for Pat_E668A, *n =* 19/3 for Pat_V1393M, *n =* 27/5 for Ctr, *n =* 20/4 for Ctr_N390K, *n =* 19/3 for Ctr_R1434Q, and *n =* 23/5 for Ctr_P1862Rfs^∗^9. (E) Quantification of the percentage fragmented over total network bursts at DIV49. *n =* 27/5 for Ctr, *n =* 20/4 for Ctr_N390K, *n =* 17/3 for Ctr_R1434Q, and *n =* 23/5 for Ctr_P1862Rfs^∗^9. Data are represented as mean ± standard error of the mean (SEM). Kruskal-Wallis test with Dunn’s test for multiple comparisons. (F) Network activity parameters over DIV35, 42, and 49 for the different isogenic lines, including network burst frequency, inter-burst interval (IBI) coefficient of variation (CoV), IBI, mean inter-spike interval (ISI) within burst, burst percentage, and network burst percentage. *n =* 27/5 for Ctr, *n =* 20/4 for Ctr_N390K, *n = 19/3* for Ctr_R1434Q, and *n = 23/5* for Ctr_P1862Rfs^∗^9. Data are represented as mean ± SEM. ^∗^*p* < 0.05, ^∗∗^*p* < 0.01, ^∗∗∗^*p* < 0.001, ^∗∗∗∗^*p* < 0.0001, two-way ANOVA with mixed-effects model if there are missing values, followed by Šídák’s test for multiple comparisons.

To uncover whether we could use changes in network developmental trajectories for different variant types and clinical phenotypes to classify VUS, we mimicked three variants in the Ctr iPSC line by genome editing. We mimicked a p.(N390K) missense variant from a patient presenting ataxia and migraine, a p.(R1434Q) missense variant from a patient with ataxia, epilepsy, and intellectual disability (ID), and a p.(R1857^∗^) nonsense variant in mutually exclusive exon 37A from a patient who presented with ataxia and epilepsy (Figure 5A). In literature, three other patients have been described with the latter variant, presenting isolated episodic ataxia (Figure 5A) (Graves et al., 2008; Sintas et al., 2017). We used CRISPR-Cas9 to introduce the variants into Ctr, resulting in an exact mimic of p.(N390K) and p.(R1434Q) missense variants. These missense variants are classified as likely pathogenic by AlphaMissense (Figures 5B; Table 1) (Cheng et al., 2023). The p.(R1857^∗^) nonsense variant was mimicked through deletion of five base pairs in exon 37A, predicting a p.(P1862Rfs^∗^9) frameshift variant. To revert to a diagnostic framework with a simplified model, we differentiated these iPSCs into glutamatergic-only neuronal networks. We recorded their neuronal network activity over development and investigated how neuronal network activity compared to the Ctr and patient-derived networks in UMAP space (Figures 5C and 5D). Ctr and Ctr-derived mutant networks clustered distinctly, with each Ctr-derived mutant network forming a separate cluster. This suggests that introduction of these variants altered the control network function, all in a variant-specific manner. Ctr_R1434Q and Ctr_P1862Rfs^∗^9 networks clustered closer to each other, suggesting some similarity in neurophysiological changes. These clusters did not overlap with the previously identified clusters (Figure 5D), preventing to link these variants to patient-derived networks based on their full network functional profile. Because epilepsy was reported in two of the patients harbouring VUS [p.(R1434Q) and p.(R1857^∗^)], we first asked whether any of the recorded networks showed an increase in the percentage of fragmented network bursts. None of the networks demonstrated a change in the percentage of fragmented network bursts (Figure 5E), suggesting that these variants cannot fully be linked to seizurogenic activity. The same *CACNA1A* variant can lead to diverse clinical phenotypes depending on the genetic background (Lipman et al., 2022), as evidenced by p.(R1857^∗^) manifesting as either isolated ataxia or a combination of ataxia and epilepsy. Therefore, introduction of variants p.(R1434Q) and p.(R1857^∗^) in Ctr may not be sufficient to cause seizurogenic activity.

**Table 1:** AlphaMissense predictions of *CACNA1A* missense variants of uncertain significance

Genetic variant	Coding variant	Protein variant	Pathogenicity score	Class	pLDDT score of original amino acid
Chr19: g.13334406A>T	c.1170T>A	p.(N390K)	0.975	likely pathogenic	70.54
Chr19: g.13259654C>T	c.4301G>A	p.(R1434Q)	0.997	likely pathogenic	88.45

Genetic variants are listed in GRCh38; coding variants are listed in NM_001127221.1.

pLDDT, predicted local distance difference test.

We then investigated whether these networks resembled any developmental trajectories as characterized for the patient-derived neuronal networks. Notably, network burst frequency was generally higher in Ctr than in the rescue networks (Figure 2, Figure 5C and 5F). In addition, all introduced variants led to higher network burst frequencies compared to Ctr at DIV35. In terms of developmental trajectories, Ctr_R1434Q and Ctr_P1862Rfs^∗^9 resembled Ctr and Rescue_V1393M, whereas Ctr_N390K resembled the developmental trajectory of Pat_E668A (Figure 2, Figure 5C and 5F). Interestingly, these variants clinically both lead to ataxia combined with migraine, indicating that this developmental trajectory might be coupled to migraine as a clinical phenotype. Both Ctr_N390K and Ctr_P1862Rfs^∗^9 showed a consistent elevated IBI CoV compared to Ctr over development, as well as a decreased network burst percentage (Figure 5F). The IBI remained unaltered, and we observed a higher mean ISI within burst and a lower burst percentage, which could be indicative of a loss-of-function effect of the variants.

Together, these results show functional changes in neuronal networks carrying *CACNA1A* VUS, supporting classification as likely pathogenic, as indicated for the missense variants by AlphaMissense. However, careful interpretation is required. By introducing these variants into a control line, it did not allow us to directly link functional changes to specific clinical phenotypes, and neurophysiological changes may vary depending on the genetic background.

## Discussion

MEA-based neuronal networks have been widely used to investigate genotype-phenotype correlations across various neurological disorders, including autism spectrum disorder, epilepsy, and Fragile X syndrome (Nageshappa et al., 2016; Deneault et al., 2019; Flaherty et al., 2019; Frega et al., 2019; Klein Gunnewiek et al., 2020; Tidball et al., 2020; Utami et al., 2020; Negraes et al., 2021; Que et al., 2021; Simkin et al., 2021; Trujillo et al., 2021; Linda et al., 2022; Mossink et al., 2022; Wang et al., 2022; van Hugte et al., 2023). These studies, using patient iPSC-derived and CRISPR-engineered neuronal networks, have demonstrated how disease-associated genetic variants can lead to alterations in network excitability, burst dynamics, and synchronization. In this study, we extend these approaches by performing a comprehensive characterization of the full network neurophysiological profile in *CACNA1A*-related disorders, integrating multiple electrophysiological parameters to derive network-level fingerprints. This systematic analysis allowed us to relate specific variant types and clinical phenotypes to distinct patterns of network activity, thereby contributing a more quantitative framework for interpreting functional consequences of *CACNA1A* variants. We showed that *null* alleles induced subtle changes in glutamatergic network activity, while missense variants exerted a more pronounced effect on network function. While network fingerprints remained relatively similar in case of dystonia co-occurring with ataxia, the network fingerprint appeared to be significantly different when clinical features such as migraine or epilepsy were present. We furthermore showed functional changes in neuronal networks when VUS were introduced in *CACNA1A*, supporting their classification as likely pathogenic. However, we could not relate these functional changes to specific clinical phenotypes as the network functional profile did not overlap with those of patient-derived networks and because the variants were introduced in a control line. CRISPR-Cas9 enables the introduction or correction of variants in iPSCs with an identical genetic background, providing a powerful approach to isolate the specific effects of a given variant. However, a variant’s effect on cellular behaviour may be influenced by the broader genetic context, meaning it might not consistently manifest a disease-relevant effect across different backgrounds. This challenge is particularly evident when control iPSC lines are used as donors for introducing genetic variants, as they may lack the patient’s unique genomic composition, potentially missing gene-gene interactions that contribute to disease pathogenesis. This limitation is particularly pronounced in the study of voltage-gated ion channels, where genetic modifier alleles can influence phenotypic variability through compensatory mechanisms. For instance, the broad phenotypic spectrum observed in *CACNA1A*-related disorders suggests the involvement of genetic modifiers. A study identified *UBR4* and *SLC1A3* as potential genetic modifiers that may exert a synergistic effect on *CACNA1A* variants, influencing disease severity and presentation (Choi et al., 2017). Furthermore, certain variants in *CACNA1A* were associated with earlier onset of seizures in individuals with Dravet syndrome, suggesting a modifying effect of these *CACNA1A* variants on the epileptic phenotype (Ohmori et al., 2013). However, whether a similar correlation exists for symptom onset in *CACNA1A*-related disorders remains to be determined. To address these complexities, we generally recommend the use of patient-derived cells in combination with multiple control lines to establish a well-characterized reference framework. Ideally, cells should be sourced from families in which all members carry the same variant but exhibit either phenotypic variability or a consistent phenotype. This approach allows for definitive conclusions about genotype-phenotype correlations while minimizing the influence of genetic background. When using CRISPR-Cas9, we suggest examining the variant of interest across multiple control genetic backgrounds to account for potential background-specific effects. Additionally, introducing known benign variants into the same iPSC lines can serve as essential controls to distinguish variant-specific effects from those arising due to genetic background variability or the editing process itself.

We here report a clear network fingerprint related to gain-of-function of Ca_V_2.1. We identified certain network parameters, such as increased IBI CoV and fragmented network bursts, that are specifically modulated by the p.(V1393M) variant. Notably, the observation that this gain-of-function variant leads to network burst fragmentation is particularly interesting, as fragmentation has been linked to asynchronous neurotransmitter release driven by increased presynaptic calcium availability (Doorn et al., 2024; Pradeepan et al., 2024). This asynchronous release can in turn prolong excitation and enhance short-term synaptic depression (Doorn et al., 2024). Indeed, other gain-of-function variants have also been associated with enhanced short-term synaptic depression (Tottene et al., 2009; Di Guilmi et al., 2014), suggesting that network burst fragmentation may be a common feature of Ca_V_2.1 gain-of-function.

The p.(V1393M) variant is recurrently observed among individuals with *CACNA1A*-related disorders (Lipman et al., 2022). Although the specific clinical phenotypes associated with this variant varied slightly among patients, ataxia and epilepsy are core features (Hamdan et al., 2017; Travaglini et al., 2017; Jiang et al., 2019; Zhang et al., 2020; Le Roux et al., 2021; Lipman et al., 2022; Niu et al., 2022; Kessi et al., 2023). This suggests that ataxia and epilepsy are a primary manifestation of the variant, independent of the patient’s genetic background. This enabled us to correlate specific network parameters to the consistent clinical features associated with this variant, even when the variant was introduced into a control iPSC line. The fact that we could not replicate a seizurogenic network fingerprint for one of the VUS, p.(R1857^∗^), may be due to the fact that this variant does not lead to epilepsy in different genetic backgrounds. Three other patients have been reported in literature with this variant, all of them showing classic episodic ataxia type 2 (Graves et al., 2008; Sintas et al., 2017). Therefore, mimicking this variant in a control line may not lead to seizurogenic activity. To determine whether the variant is the underlying cause of epilepsy in our proband, it would be crucial to use patient-derived iPSCs and include a family member as donor to control for potential confounding genetic factors.

Another possibility is that the chosen model may not fully capture variant-related effects. The effects of the variant could manifest in other cell types than the iPSC-derived glutamatergic neurons being studied. Since the variant is located in exon 37A—a mutually exclusive exon that undergoes a developmental switch—its expression dynamics may influence neurophysiological outcomes. Early in development, exon 37B is predominantly expressed, while expression of exon 37A gradually increases postnatally (Vigues et al., 2002; Chaudhuri, et al. 2004, 2005). If exon 37A is not sufficiently expressed in the current model, the expected fingerprint may not be detectable. In the adult brain, both isoforms are generally expressed at similar levels across most regions (Bourinet et al., 1999; Soong et al., 2002; Vigues et al., 2002; Chaudhuri et al., 2005); however, at the cellular level, distinct expression patterns emerge. Parvalbumin-positive interneurons in particular seem to rely exclusively on exon 37A (Huntley et al., 2020), suggesting that a more comprehensive model—including both glutamatergic and GABAergic neurons—may be necessary to fully capture the variant’s effect.

We showed that a *CACNA1A* gain-of-function variant exerted effects on GABAergic neuronal functioning specifically, by co-culturing control glutamatergic neurons with p.(V1393M) GABAergic neurons. We demonstrated that p.(V1393M) GABAergic neurons showed an increased inhibitory drive in early development of the network. At this moment of neuronal network development, control networks may not have undergone the GABA shift (Mossink et al., 2022)—an increase in the KCC2:NKCC1 chloride cotransporter expression through which the chloride reversal potential hyperpolarizes (Ben-Ari et al., 2007). It remains to be determined whether the observed increase in inhibitory drive represents an early GABA shift or results from enhanced GABA release at the synapse. Interestingly, loss of Ca_V_2.1 function compromised GABA release from parvalbumin-positive interneurons specifically, whereas signaling was preserved from somatostatin-positive interneurons (Rossignol et al., 2013). Because the p.(V1393M) variant has been shown to lead to gain-of-function, the increased inhibitory drive may also stem from increased GABA release. Although we have not characterized the specific interneuron subtypes within our glutamatergic/GABAergic networks, we demonstrate an increased inhibitory drive that is independent of genetic background. Therefore, we propose that these networks serve as a valuable platform for exploring genotype-phenotype correlations and their impact on neurophysiological activity in *CACNA1A*-related disorders.

Our findings further underscore the potential of specific network metrics, such as mean ISI within burst, burst percentage, and fragmented network bursts, as biomarkers for guiding variant-specific therapeutic strategies. For example, networks carrying variants like p.(V1393M), which exhibit fragmented bursting, may benefit from treatments targeting presynaptic calcium handling. In contrast, *null* alleles, with more subtle network effects in glutamatergic networks, may require modelling in glutamatergic/GABAergic co-cultures and treatments targeting the inhibitory circuit. These observations highlight the need for stratified treatment strategies based on the specific functional fingerprint of each variant.

We also demonstrate that genetic background can modulate these fingerprints, which may complicate the interpretation and translational relevance of functional findings. While some variant effects (e.g., p.(V1393M)-induced inhibitory drive) appear to be independent of genetic background, others, particularly VUS introduced into control lines, did not recapitulate expected phenotypes. This discrepancy may reflect the absence of disease-relevant modifier alleles in unrelated control lines. These observations raise the question whether functional studies should rely more heavily on patient-derived iPSC models, particularly for variants where the clinical presentation is variable or unclear. We propose that, where resources allow, validating findings in patient-derived cells provides a more reliable framework for understanding disease mechanisms. This approach may be especially valuable in the context of precision medicine, where both variant-specific and background-sensitive phenotypes could inform tailored therapeutic strategies.

### Limitations of the study

Although we used multiple patient-derived neuronal lines, including some with CRISPR-Cas9-corrected/mimicking variants, the statistical power of this study is not sufficient to establish definitive genotype-phenotype correlations. Instead, our findings should be viewed as a proof of concept, highlighting both the strengths and limitations of iPSC-based neuronal modelling. One key limitation is the challenge of interpreting CRISPR-engineered VUS. While CRISPR-Cas9 remains a standard tool in the field, these engineered lines lack direct clinical data, making it difficult to contextualize their functional impact. Unlike patient-derived lines, CRISPR-edited lines do not inherently reflect the complex genetic and epigenetic background of an affected individual, which may influence neuronal network activity. This underscores the need for complementary approaches, such as functional assays in patient-derived cells and longitudinal clinical studies, to improve variant classification and our understanding of *CACNA1A*-related disorders.

## Methods

### Human iPSC lines

In this study, we used a total of eleven iPSC lines, derived from five distinct individuals (supplemental methods, Figure S3). Informed consent was obtained from participants to collect and process their data. The institutional ethical review committee CMO Radboudumc, Nijmegen, the Netherlands, has given approval to conduct this research (CMO Radboudumc dossier numbers: 2017–3827, 2019–5250).

### Generation of rtTA-*Ngn2*- or rtTA-*Ascl1/Dlx2*-positive iPSCs and neuronal differentiation

To differentiate iPSCs into glutamatergic neurons, rtTA-*Ngn2*-positive iPSCs were generated according to a previously published protocol (supplemental methods) (Zhang et al., 2013; Frega et al., 2017). In order to differentiate iPSCs into GABAergic neurons, rtTA-*Ascl1*/*Dlx2*-positive iPSCs were generated according to a recently published protocol (supplemental methods) (Yang et al., 2017; van Voorst et al., 2025). At 80%–90% confluency (DIV0), single cells were generated from rtTA-*Ngn2*-positive or rtTA-*Ascl1*-*Dlx2*-positive iPSCs for neuronal differentiation (supplemental methods). The cells were plated at a final density of approximately 600 cells/mm^2^. For glutamatergic and GABAergic co-cultures, cells were plated in a 50:50 ratio.

### Micro-electrode array recordings and analysis

Spontaneous neuronal network activity was recorded from neuronal networks grown on 48-well Cytoview MEAs (Axion BioSystems, Atlanta, GA, USA) for 5 min in a Maestro Pro MEA system (Axion BioSystems) equipped with AxIS Navigator software (Axion BioSystems) when temperature and CO_2_ were stable at 37°C and 5%, respectively. Within each well, 16 electrodes are embedded at the bottom in a 4 × 4 grid (diameter 50 μm, spacing 350 μm). The sampling frequency was set at 12.5 kHz. For spike detection, an adaptive threshold of ±6 standard deviations of the noise was applied, creating .spk files. Time-stamps per electrode were obtained from these raw .spk files using AxionFileLoader toolbox in MATLAB. Electrode and network bursts were detected using modified functions from the meaRtools R package (Gelfman et al., 2018). For electrode burst detection, we used the maximum ISI algorithm, setting maximum ISI to 0.1 s. Electrode bursts were restricted to be constituted by 5 spikes at least and to be longer than 0.05 s. Consecutive electrode bursts separated by less than 0.05 s were merged. For network bursts detection, time-stamps were binned in 2 ms intervals, and a Gaussian filter with a 70 ms window size was applied to the binned data. The smoothed signals from individual electrodes were standardized, combined, and then smoothed again using the same Gaussian filter. Network burst intervals were identified by thresholding the combined signal with the Otsu global thresholding algorithm, requiring the involvement of at least 25% of the electrodes and a duration of more than 0.1 s. In addition, we imposed a restriction on network bursts, requiring a minimum firing rate of 12 Hz in at least 25% of the actively participating electrodes. We identified a population of particularly short network bursts (<0.32 s) that followed “main network bursts” (>0.32 s) with no more than a 1.1-s gap (Figure 3B). These were termed “fragmented network bursts” and were analyzed separately. Various average and variability-related variables were extracted from electrode bursts, network bursts, and fragmented network bursts. These variables are detailed in Table S3.

To evaluate whether the different *CACNA1A* variants induced effects relative to the control network activity, we performed PCA on scaled parameters for DIV35, DIV42, and DIV49 using the prcomp function from the stats R package. We then applied UMAP to the PCs that accounted for more than 2.5% of the variance in the data. The number of PCs considered is indicated in the corresponding figures.

### Single-cell electrophysiology

Single-cell recordings in whole-cell patch-clamp configuration were performed at DIV42 as previously described (Hommersom et al., 2024) and further described in the supplemental methods. We performed PCA on scaled parameters using the prcomp function from the stats R package. We then applied UMAP to the PCs that accounted for more than 5% of the variance in the data.

### Compounds

All compounds were prepared into concentrated stocks in dimethyl sulfoxide (DMSO) and stored at −20°C, unless mentioned otherwise. Glutamatergic-only cultures were treated over development with Cav2 inhibitors, PD-173212 (Sigma-Aldrich, #SML3506) and CNV2197944 (CNV’944; provided by the CACNA1A Foundation; stored at 4°C). Upon treatment, these compounds were first diluted 1:20 in Dulbecco’s phosphate-buffered saline with calcium and magnesium (Gibco, #14040117). Treatment started at DIV22 by diluting the compound 1:100 into the full volume of medium of the well. Every time the medium was refreshed afterward, the compound was diluted 1:100 into half of the volume of the well, resulting in maximum final concentrations of 500 nM for PD-173212 and 30 μM for CNV’944. We added DMSO as a vehicle in the same way, resulting in a maximum final DMSO percentage of 0.05%. We assessed network activity at DIV49.

At DIV34, we collected 50% pre-conditioned medium from glutamatergic/GABAergic co-cultures, while refreshing 50% of the medium. At DIV35, we assessed the basal network activity of these co-cultures for 5 min. After this recording, co-cultures were treated with 100 μM PTX (in 96% ethanol; Tocris, #1128) or 0.1% ethanol. We immediately assessed spontaneous network activity without GABAergic signaling for 20 min. Consecutively, PTX and vehicle were washed out by three gentle washes with plain Neurobasal medium (Gibco, #21103049), and medium was fully refreshed by combining the pre-conditioned medium in a 1:1 ratio with freshly prepared Neurobasal medium with supplements (supplemental methods).

### Statistics

Statistical analysis was performed using GraphPad PRISM 10.1.2 (GraphPad Software, Inc., CA, USA). Each figure legend indicates how data are visualized and what tests were performed. In all figures, *p* values are indicated as follows: < 0.05 (^∗^), < 0.01 (^∗∗^), < 0.001 (^∗∗∗^), < 0.0001 (^∗∗∗∗^). In each figure legend, *n =* (*a*)/(*b*) indicates the number of MEA wells or patched cells (*a*) per independent differentiation (*b*). We provide a summary of every statistical test performed per figure panel in Table S4.

## Resource availability

### Lead contact

Requests for further information and resources should be directed to and will be fulfilled by the lead contact, Hans van Bokhoven (hansvanbokhoven@radboudumc.nl).

### Materials availability

This study did not generate new unique reagents apart from iPSC lines.

### Data and code availability

All data reported in this paper will be shared by the lead contact upon request. All original code has been deposited at GitHub and is publicly available at https://github.com/nadifkasri-lab/hommersom_2025_cacna1a_variants as of the date of publication. Any additional information required to reanalyze the data reported in this paper is available from the lead contact upon request.

## Acknowledgments

This research was made possible through the CACNA1A Foundation, with samples available from the COMBINEDBrain Biorepository. We wish to thank the Radboudumc Stem Cell Technology Center (https://www.radboudumc.nl/en/research/radboud-technology-centers/stem-cells) for genome editing and characterizing the Rescue_V1393M iPSC line. This work was supported by a grant from the 10.13039/501100006209Radboud University Medical Center and Donders Institute for Brain, Cognition, and Behaviour (B.P.C.v.d.W. and H.v.B.) and a grant from the CACNA1A Foundation (B.P.C.v.d.W. and M.P.H.). N.N.K. was supported by the BRAINMODEL ZonMw PSIDER program 10250022110003 and 10.13039/100014370SFARI grant 890042.

## Author contributions

B.P.C.v.d.W., N.N.K., and H.v.B. conceptualized and supervised the study. M.P.H. wrote the original draft of this manuscript and was responsible for the visualization of the data. B.P.C.v.d.W., N.N.K., and H.v.B. reviewed and edited the manuscript. M.P.H. and E.C. performed CRISPR-Cas9 experiments to generate iPSC models. L.D. performed experiments for the validation of iPSC lines. M.B. and E.v.B. performed MEA experiments together with M.P.H. S.P. created the MATLAB and R codes for analysis of the MEA data and provided support when these were executed by M.P.H. M.P.H. performed single-cell patch-clamp experiments and performed subsequent analyses.

## Declaration of interests

The authors declare no competing interests.

## Declaration of generative AI and AI-assisted technologies in the writing process

During the preparation of this work, the authors used ChatGPT to improve language and readability. After using this tool/service, the authors reviewed and edited the content as needed and take full responsibility for the content of the publication.

## Supplemental information

Document S1. Figures S1–S3, Tables S1–S3, S5, and S6, and supplemental methods

Table S4. Statistical data per figure panel

Document S2. Article plus supplemental information
