# Supplementary material for: Human neuronal networks on micro-electrode arrays as a tool to assess genotype-phenotype correlation in CACNA1A-related disorders
Source: Stem Cell Reports. 2026 Jan 22;21(2):102783. doi: 10.1016/j.stemcr.2025.102783 (PMC12903092; doi:10.1016/j.stemcr.2025.102783)
Supplement: Document S1. Figures S1–S3, Tables S1–S3, S5, and S6, and supplemental methods [file mmc1.pdf]

Stem Cell Reports, Volume *21*

Supplemental Information

Human neuronal networks on micro-electrode arrays as a tool to assess genotype-phenotype correlation in *CACNA1A*-related disorders

Marina P. Hommersom, Sofía Puvogel, Nicky Scheefhals, Eleonora Carpentiero, Marga Bouma, Ellen van Beusekom, Lieke Dillen, Bart P.C. van de Warrenburg, Nael Nadif Kasri, and Hans van Bokhoven

Supplemental Information

Supplemental Figures

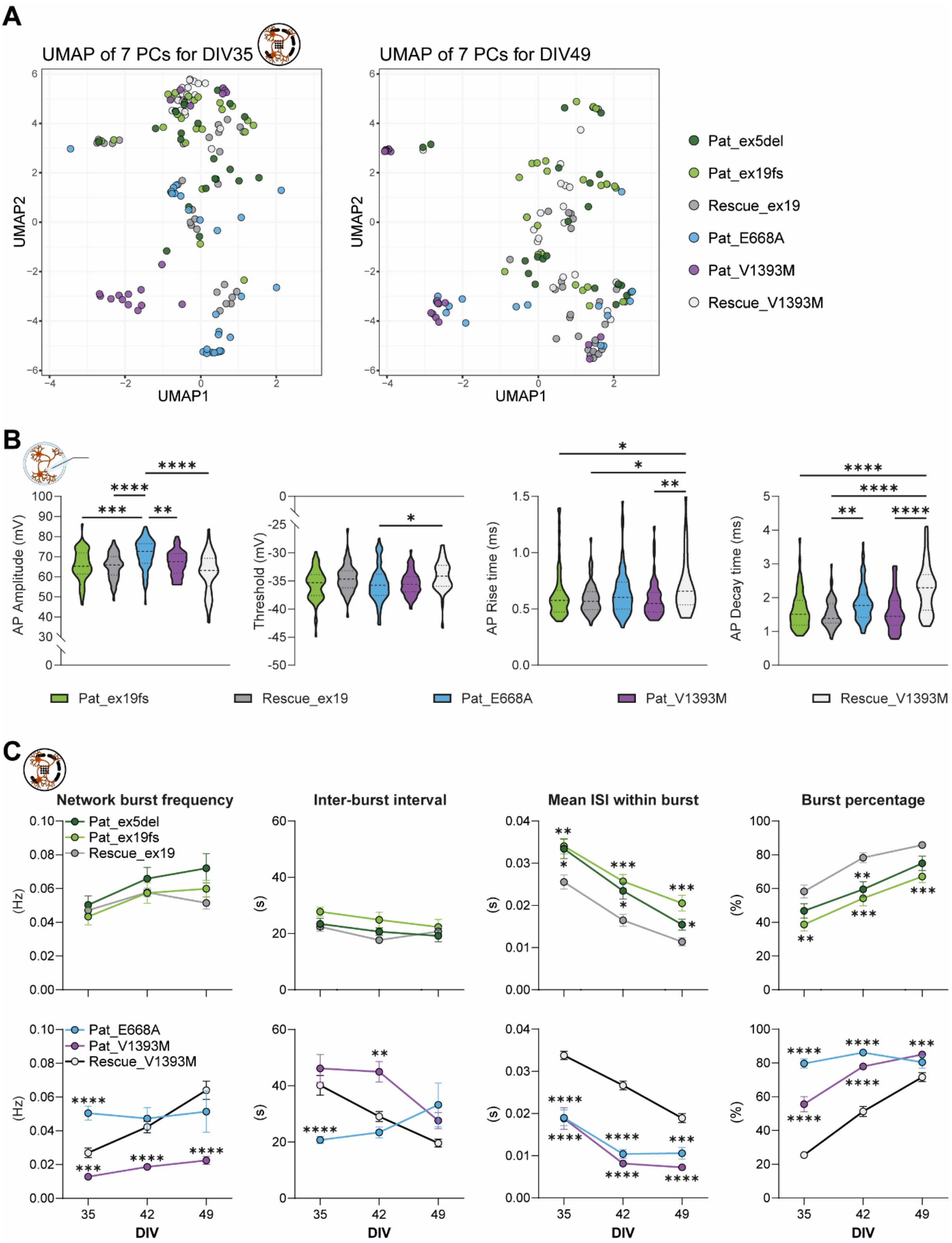

Figure S1 Quantification of single-cell and network electrophysiological parameters of patient-derived glutamatergic neurons, related to Figure 1 and 2. (**A**) Uniform manifold approximation and projection (UMAP) analysis on seven principal components (PCs) obtained from network activity parameters at days *in vitro* (DIV)35, and 49 for all iPSC-derived networks indicated in Figure 1A. (**B**) Quantification of single-cell electrophysiological properties including action potential (AP) amplitude, threshold, rise time and decay time at DIV42. *n = 68/8* for Pat_ex19fs, *n = 63/7* for Rescue_ex19, *n = 73/5* for Pat_E668A, *n = 57/4* for Pat_V1393M, and *n = 41/2* for Rescue_V1393M. Dashed line

represents the median, dotted line represents the quartiles. *P < *0.05, ***P < *0.01* ***P < 0.001, ****P < 0.0001. Kruskal-Wallis test with Dunn's test for multiple comparisons. (**C**) Network activity parameters over days *in vitro* (DIV)35, 42, and 49 for the patient and isogenic rescue lines, including network burst frequency, inter-burst interval, mean inter spike interval (ISI) within burst, and burst percentage. *n = 21/3* for Pat_ex5del, *n = 25/4* for Pat_ex19fs, *n = 29/4* for Rescue_ex19, *n = 27/4* for Pat_E668A, *n = 19/3* for Pat_V1393M, and *n = 20/2* for Rescue_V1393M. Data represented as mean ± standard error of the mean (SEM). *P < 0.05, **P < 0.01, ***P < 0.001, ****P < 0.0001. Two-way ANOVA with mixed-effects model if there are missing values, followed by Šídák's test for multiple comparisons.

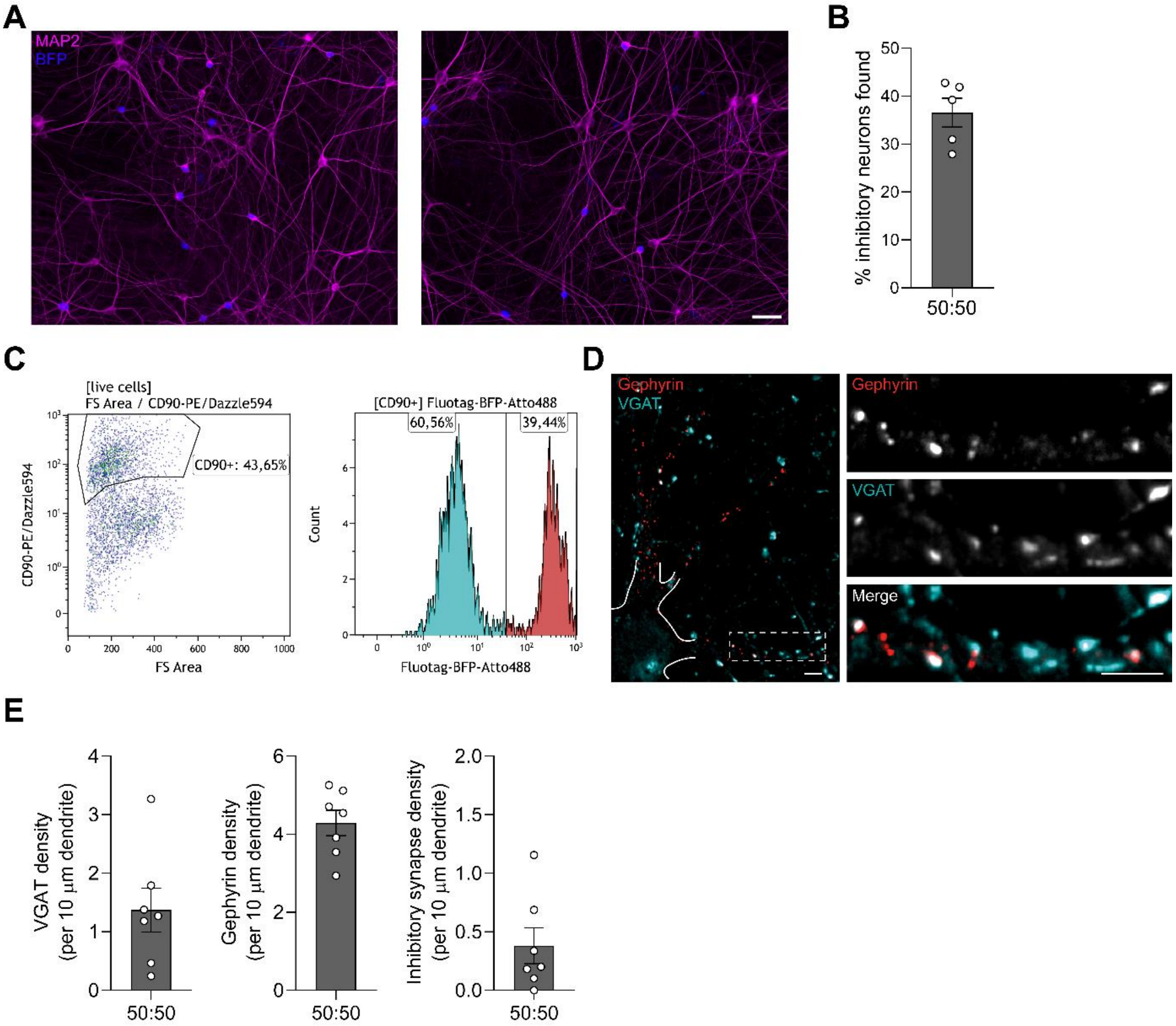

Figure S2 Characterization of glutamatergic/GABAergic co-cultures, related to Figure 4. (**A**) Representative images of 50:50 Ctr:Ctr glutamatergic/GABAergic co-cultures at days *in vitro* (DIV)49 (scale bar = 50 µm). Neurons are stained for microtubule-associated protein 2 (MAP2, in magenta) and GABAergic neurons are show blue fluorescent protein (BFP, in blue)-positive nuclei. (**B**) Quantification of the percentage of GABAergic neurons (BFP-positive nuclei) in 50:50 Ctr:Ctr glutamatergic/GABAergic co-cultures at DIV49. *n = 5/2*. Data represent means ± standard error of the mean (SEM), with individual datapoints representing the mean percentage per coverslip. (**C**) Quantification of the percentage of GABAergic neurons in 50:50 Ctr:Ctr glutamatergic/GABAergic co-cultures at DIV49 by flow cytometry. Cells were first gated for $CD90^+$ events to select neurons and exclude astrocytes prior to the gating of neurons positive for BFP (GABAergic neurons). (**D**) Representative image of 50:50 Ctr:Ctr glutamatergic/GABAergic co-cultures stained with Gephyrin and VGAT to label GABAergic synapses (scale bar = 5 µm). (**E**) Quantification of the number of VGAT, Gephyrin and co-localized VGAT/Gephyrin puncta per 10 µm dendritic length. *n = 7.* Data represent means ± SEM, with individual datapoints representing the mean puncta density per neuron.

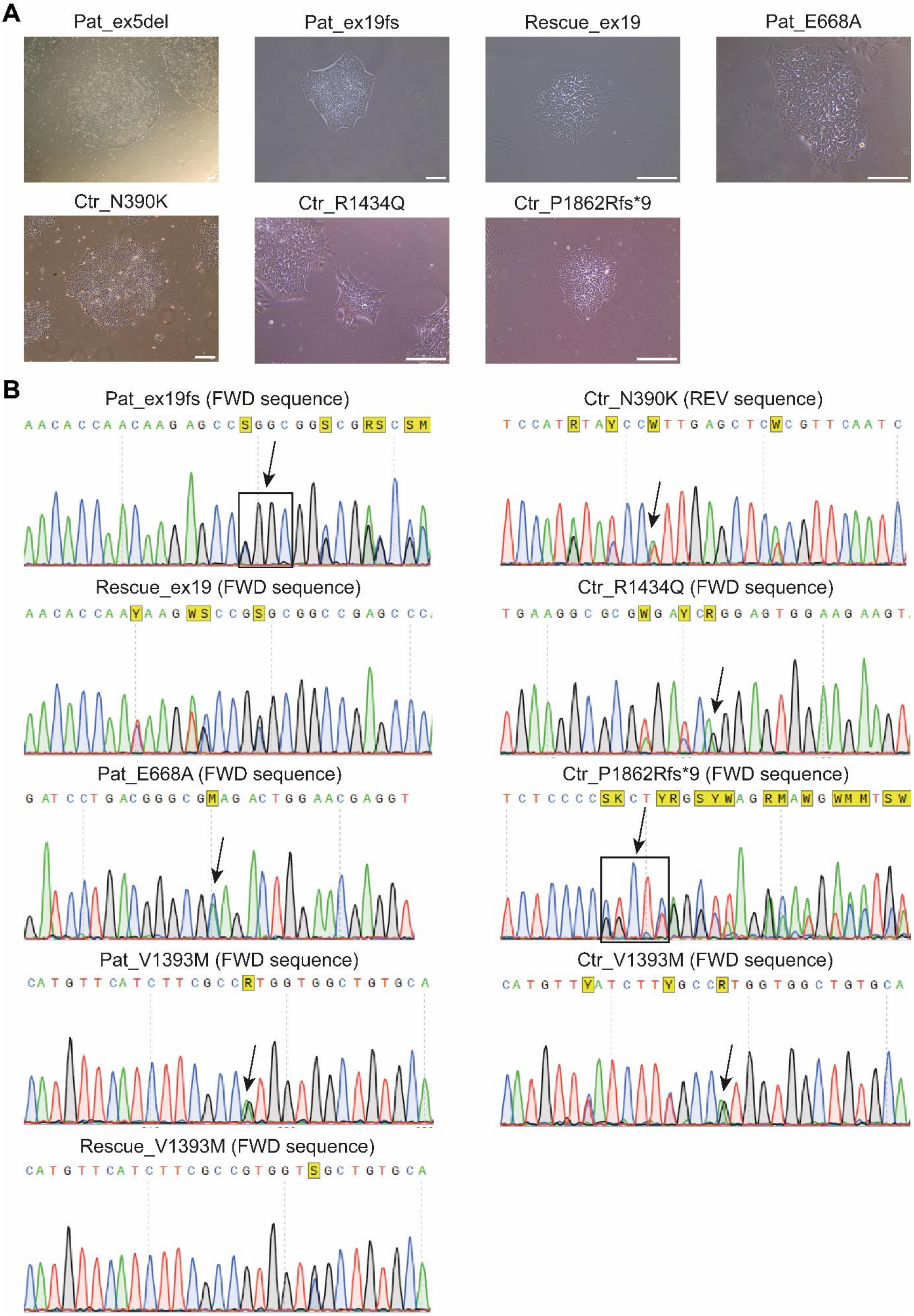
A
Pat_ex5del
Pat_ex19fs
Rescue_ex19
Pat_E668A
Ctr_N390K
Ctr_R1434Q
Ctr_P1862Rfs*9
B
Pat_ex19fs (FWD sequence)
Ctr_N390K (REV sequence)
Rescue_ex19 (FWD sequence)
Ctr_R1434Q (FWD sequence)
Pat_E668A (FWD sequence)
Ctr_P1862Rfs*9 (FWD sequence)
Pat_V1393M (FWD sequence)
Ctr_V1393M (FWD sequence)
Rescue_V1393M (FWD sequence)

Figure S3 Characterization of the included cell lines in this study, related to Figure 1 and 5. (**A**) Induced pluripotent stem cell (iPSC) colonies all showed characteristic morphology. Scale bar = 200 µm. (**B**) Sanger sequences of cell lines used in this study. Arrows indicate the patient or CRISPR-induced variants. In CRISPR lines, silent variants were introduced to disturb guide RNA recognition. Above each sequence, it is indicated whether the forward (FWD) or reverse (REV) sequence is shown.

Supplemental Tables

Table S1 Top 10 parameters contributing to each PC related to Figure 2.

PC	Top 10 Parameter Rescues	PC	Top 10 Parameter Patients
PC1	Burst_Percentage_Avg	PC1	Firing_Rate_in_NB_Avg
	Random_Spikes_Percentage_Avg		total_spikes_in_all_NBs_d_nNB
	Mean_ISI_within_Burst_Avg_sec		Spike_Frequency_per_Electrode
	Number_of_Bursting_Electrodes		Mean_ISI_within_Burst_Avg_sec
	Number_of_spikes		Mean_ISI_within_Burst_Std_sec
	Mean_Firing_Rate_Hz		Number_of_Spikes_per_Burst_Avg
	Spike_Frequency_per_Electrode		Burst_Percentage_Avg
	Firing_Rate_in_NB_Avg		Random_Spikes_Percentage_Avg
	Number_of_Spikes_per_Burst_Avg		Burst_Percentage_Std
	total_spikes_in_all_NBs_d_nNB		hf_Spike_Frequency_per_Electrode
PC2	total_spikes_in_all_hf_NBs_d_nNB	PC2	hf_Network_Burst_Duration_Avg_sec
	hf_Network_Burst_Frequency		Inter_Burst_Interval_Avg_sec
	Number_of_hf_Network_Bursts		Number_of_hf_Network_Bursts
	hf_Network_Burst_Duration_Avg_sec		hf_Network_Burst_Frequency
	hf_Firing_Rate_in_NB_Avg		Network_Burst_Duration_Std_sec
	hf_Spike_Frequency_per_Electrode		total_spikes_in_all_hf_NBs_d_nNB
	hf_Network_Burst_Percentage		Burst_Frequency_Avg_Hz
	Firing_Rate_in_NB_std		hf_Spike_Frequency_per_Electrode
	hf_Network_Burst_Duration_Std_sec		hf_Firing_Rate_in_NB_Avg
	hf_Firing_Rate_in_NB_std		hf_Network_Burst_Percentage
PC38	hf_Network_Burst_Frequency	PC38	Number_of_hf_Network_Bursts
	Number_of_hf_Network_Bursts		hf_Network_Burst_Frequency
	Random_Spikes_Percentage_Avg		Burst_Percentage_Avg
	Burst_Percentage_Avg		Random_Spikes_Percentage_Avg
	Number_of_Network_Bursts		Network_Burst_Frequency
	Network_Burst_Frequency		Number_of_Network_Bursts
	hf_Spike_Frequency_per_Electrode		hf_Spike_Frequency_per_Electrode
	hf_Firing_Rate_in_NB_Avg		hf_Firing_Rate_in_NB_Avg
	total_spikes_in_all_hf_NBs_d_nNB		hf_Network_Burst_Percentage
	Firing_Rate_in_NB_Avg		Firing_Rate_in_NB_Avg
PC39	Random_Spikes_Percentage_Avg	PC39	Burst_Percentage_Avg
	Burst_Percentage_Avg		Random_Spikes_Percentage_Avg
	hf_Network_Burst_Frequency		Number_of_hf_Network_Bursts
	Number_of_hf_Network_Bursts		hf_Network_Burst_Frequency
	Number_of_Network_Bursts		Network_Burst_Frequency
	Network_Burst_Frequency		Number_of_Network_Bursts
	Firing_Rate_in_NB_Avg		hf_Spike_Frequency_per_Electrode
	Spike_Frequency_per_Electrode		hf_Firing_Rate_in_NB_Avg
	hf_Firing_Rate_in_NB_Avg		Number_of_Bursts
	hf_Spike_Frequency_per_Electrode		total_spikes_in_all_NBs_d_nNB

Table S2 Top 10 parameters contributing to each PC related to Figure 3.

PC	Top 10 Parameter controls	PC	Top 10 Parameter p.(V1393M)
PC1	Spike_Frequency_per_Electrode	PC1	Mean_Firing_Rate_Hz
	Firing_Rate_in_NB_Avg		Number_of_spikes
	Mean_Firing_Rate_Hz		Burst_Frequency_Avg_Hz
	Number_of_spikes		Number_of_Bursts
	Mean_ISI_within_Burst_Avg_sec		Number_of_Spikes_per_Burst_Avg
	total_spikes_in_all_NBs_d_nNB		Burst_Percentage_Avg
	Number_of_Spikes_per_Burst_Avg		Random_Spikes_Percentage_Avg
	Burst_Percentage_Avg		total_spikes_in_all_NBs_d_nNB
	Random_Spikes_Percentage_Avg		Mean_ISI_within_Burst_Avg_sec
	Burst_Percentage_Std		Inter_Burst_Interval_Avg_sec
PC2	hf_Firing_Rate_in_NB_std	PC2	Network_Burst_Percentage
	Number_of_hf_Network_Bursts		hf_Network_Burst_Percentage
	hf_Network_Burst_Frequency		IBI_Coefficient_of_Variation_Avg
	hf_Spike_Frequency_per_Electrode		hf_Network_Burst_Duration_Avg_sec
	total_spikes_in_all_hf_NBs_d_nNB		Firing_Rate_in_NB_std
	hf_Network_Burst_Percentage		hf_Firing_Rate_in_NB_Avg
	hf_Network_Burst_Duration_Avg_sec		hf_Spike_Frequency_per_Electrode
	hf_Network_Burst_Duration_Std_sec		hf_Firing_Rate_in_NB_std
	hf_Firing_Rate_in_NB_Avg		total_spikes_in_all_hf_NBs_d_nNB
	Firing_Rate_in_NB_std		IBI_Coefficient_of_Variation_Std
PC28	Number_of_spikes	PC37	Number_of_Network_Bursts
	Mean_Firing_Rate_Hz		Network_Burst_Frequency
	Firing_Rate_in_NB_Avg		hf_Network_Burst_Frequency
	Spike_Frequency_per_Electrode		Number_of_hf_Network_Bursts
	Number_of_Bursts		Burst_Percentage_Avg
	total_spikes_in_all_NBs_d_nNB		Random_Spikes_Percentage_Avg
	Number_of_Spikes_per_Burst_Avg		Mean_Firing_Rate_Hz
	NBI_Avg_sec		Number_of_spikes
	Inter_Burst_Interval_Avg_sec		Number_of_Bursts
	Mean_ISI_within_Burst_Avg_sec		Burst_Frequency_Avg_Hz
PC29	Firing_Rate_in_NB_Avg	PC38	Random_Spikes_Percentage_Avg
	Spike_Frequency_per_Electrode		Burst_Percentage_Avg
	Mean_Firing_Rate_Hz		Number_of_Network_Bursts
	Number_of_spikes		Network_Burst_Frequency
	Burst_Frequency_Avg_Hz		Number_of_hf_Network_Bursts
	Number_of_Bursts		hf_Network_Burst_Frequency
	hf_Firing_Rate_in_NB_std		Number_of_spikes
	Number_of_Network_Bursts		Mean_Firing_Rate_Hz
	Number_of_Spikes_per_Burst_Avg		Number_of_Bursts
	Network_Burst_Frequency		Burst_Frequency_Avg_Hz

Table S3 Description of extracted MEA parameters.

Metric type	Parameter	Unit	Description
Single spikes	Number of spikes		Total number of spikes over the duration of the analysis.
	Mean firing rate	Hz	Total number of spikes divided by the duration of the analysis.
Electrode bursts	Number of bursts		Total number of single-electrode bursts over the duration of the analysis. For a well, the total number of electrode bursts across all electrodes in a well is reported.
	Number of bursting electrodes		Total number of electrodes within the well with bursts/minute greater than the burst electrode criterion.
	Burst duration (Avg)	s	Average time from the first spike to last spike in a single-electrode burst. For a well, the average across electrode averages is reported.
	Burst duration (Std)	s	The standard deviation across electrode burst durations.
	Number of spikes per burst (Avg)		Average number of spikes in a single-electrode burst. For an electrode, the average across bursts is reported. For a well, the average across electrode averages is reported.
	Number of spikes per burst (Std)		The standard deviation across electrode numbers of spikes per burst.
	Mean ISI within burst (Avg)	s	Mean inter-spike interval, time between spikes, for spikes in a single-electrode burst. For a well, the average across electrode averages is reported.
	Mean ISI within burst (Std)	s	The standard deviation across electrode mean ISIs within burst
	Inter-burst interval (Avg)	s	Average time between the start of single-electrode bursts. For a well, the average across electrode averages is reported.
	Inter-burst interval (Std)	s	The standard deviation across electrode IBIs
	IBI Coefficient of Variation (Avg)		The coefficient of variation (standard deviation/mean) of the inter-burst interval, the time between single-electrode bursts. This is a measure of single-electrode burst regularity. For a well, the average across electrode IBI CoVs is reported.
	IBI Coefficient of Variation (Std)		The standard deviation across electrode IBI CoVs
	Burst frequency (Avg)	Hz	Total number of single-electrode bursts divided by the duration of the analysis, in Hz. For a well, the average across electrode burst frequencies is reported.
	Burst frequency (Std)	Hz	The standard deviation across electrode burst frequencies.
	Burst percentage (Avg)	%	The number of spikes in single-electrode bursts divided by the total number of spikes, multiplied by 100. For a well, the average across electrode burst percentages is reported.
	Burst percentage (Std)	%	The standard deviation across electrode burst percentages.
	Random spikes percentage(Avg)	%	The number of spikes not in single-electrode bursts divided by the total number of spikes, multiplied by 100.
Network bursts	Number of network bursts		Total number of network bursts over the duration of the analysis.
	Network burst frequency	Hz	Total number of network bursts divided by the duration of the analysis.

	Spike frequency per electrode	Hz	Total number of spikes detected in all network bursts divided by the durations of all network bursts. This represents the average firing activity within a network burst.
	Total spikes in all NBs d nNB		Total number of spikes detected in all network bursts divided by the number of network bursts. This represents the average number of spikes per network burst.
	Network burst percentage	%	The number of spikes in network bursts divided by the total number of spikes, multiplied by 100.
	Network burst duration (Avg)	s	Average time from the first spike to last spike in a network burst.
	Network burst duration (Std)	s	The standard deviation across all network burst durations.
	Firing rate in NB (Avg)	Hz	Average number of spikes occurring within a network burst divided by the duration of that network burst. This represents the average firing activity within a network burst.
	Firing rate in NB (Std)	Hz	The standard deviation across firing rates across network bursts. This represents the variability in the firing rate within network bursts.
	Network inter-burst interval (Avg)	s	Average time interval between consecutive network bursts.
	Network inter-burst interval (Std)	s	The standard deviation across all network inter-burst intervals. This represents the variability in time intervals between consecutive network bursts.
Fragments / network bursts	Number of hf network bursts		Total number of high frequency network bursts over the duration of the analysis.
	hf Network burst frequency	Hz	Total number of high frequency network bursts divided by the duration of the analysis.
	hf Spike frequency per electrode	Hz	Total number of spikes detected in all high frequency network bursts divided by the durations of all high frequency network bursts. This represents the average firing activity within a high frequency network burst.
	Total spikes in all hf NBs d nNB		Total number of spikes detected in all high frequency network bursts divided by the number of high frequency network bursts. This represents the average number of spikes per high frequency network burst.
	hf Network burst percentage	%	The number of spikes in high frequency network bursts divided by the total number of spikes, multiplied by 100.
	hf Network burst duration (Avg)	s	Average time from the first spike to last spike in a high frequency network burst.
	hf Network burst duration (Std)	s	The standard deviation across all high frequency network burst durations.
	hf Firing rate in NB (Avg)	Hz	Average number of spikes occurring within a high frequency network burst divided by the duration of that high frequency network burst. This represents the average firing activity within a high frequency network burst.
	hf Firing rate in NB (Std)	Hz	The standard deviation across firing rates across high frequency network bursts. This represents the variability in the firing rate within high frequency network bursts.

Table S5 CRISPR/Cas9 strategies and sequences used in this study, related to Figure 1 and 5.

Generated clones	Plasmid-based or RNP complex-based protocol	Forward sequence (5'-3')	Reverse sequence (5'-3')	HDR template (5'-3')	*CACNA1A* variant
Rescue_Ex19	RNP complex-based	**AACACCAACAAGAGCCCGGC**	N/A	C*G*GGCCCGATCGTGGTAGCGGGCCTGTTTCCTGAGGAAGTCCTCGGCGCGCTGCTGGCCGAGGCGCTGGTCCACGGTGGGCTCGGCCGCGCGGGACTTATTGGTGTTGTTGTTGCGGTTCTCCTG*C*G	c.2518_2519 insCGGC corrected
Rescue_V1393M	RNP complex-based	**CATGTTCATCTTCGCCATGG**	N/A	A*A*AGTGGAGACCCACCGACAATCTTTCTCAAACTCTTTGGACTCGTCAGTGCAGTGGAAGAATTTCCCCTTGAAGAGCTGCACAGCGACCACGGCGAAGATGAACATGAATAGCATGTAGACGAT*G*A	c.4177G>A corrected
Ctr_V1393M	RNP complex-based	**ATTCATGTTCATCTTCGCCG**	N/A	G*T*GGAGACCCACCGACAATCTTTCTCAAACTCTTTGGACTCGTCAGTGCAGTGGAAGAATTTCCCCTTGAAGAGCTGCACAGCCACCATGGCAAAGATAAACATGAATAGCATGTAGACGATGAG*G*A	c.4177G>A
Ctr_N390K	Plasmid-based	ccacg**ACGTGAGCTCAATGGGTACA**	aaac**TGTACCCATTGAGCTCACGT**c	C*A*TGACTCTCTTTGTACTCCGTGGCCTGGGATCTCCATCCCTGGGCCCCAGGATGAAAGGGCCTCACCTGCTTTTGAGATCCACTCCATATATCCTTTGAGCTCTCGTTCAATCTGTTGTTGCCG*C*C	c.1170T>A
Ctr_R1434Q	RNP complex-based	**TGAGGTGAAGGCGCGAGACC**	N/A	G*G*CCAGCCTTCTCCCGTGGACACGGTGAAGAGGGTCAGCAGAGCCCACAGCACATTGTCGTAATGGAATTCATACTTCTTCCACTCCTGATCACGCGCCTTCACCTCATTCTTCTCGTAGAGGAG*G*T	c.4301G>A
Ctr_P1862Rfs*9	Plasmid-based	cacc**GTTTCTTGCCTAAGCCGAGAG**	aaac**CTCTCGGCTTAGGCAAGAAAC**	N/A	c.5585_5589 del

Coding variants are listed in NM_001127221.1. Abbreviations: HDR, homology directed repair; N/A, not applicable; RNP, ribonucleoprotein

Table S6 PCR primers used in this study.

PCR primer target	Forward (5'-3')	Reverse (5'-3')
CACNA1A exon 8	GGCACAGTTGTCGGGAAATG	AACGGAGGTACAGGGATGTG
CACNA1A exon 19	TCCATCCAAGCTACAGTGCC	ATGACGTCGATGCTCCCC
CACNA1A exon 26	GTGTTGGGCTCCTGTATCAC	GGGAATGTGCTGGAAAGTGG
CACNA1A exon 27	ACCACTCTTCCTTTCCCTCC	AGCTCTCAGGCCCTTTATCC
CACNA1A exon 37	CGACTGACATCCTACACCCC	AAGAACCCCAAGCCACACTC

Supplemental Methods

Patient information and human iPSC line generation

Pat_ex5del originated from a 47-year old male, who presented symptoms of episodic ataxia type 2, with a heterozygous deletion (NM_001127221.1: c.(631_632)_(784_785)del) in exon 5 of *CACNA1A*. This line was reprogrammed from peripheral blood mononuclear cells (PBMCs) by episomal vectors expressing SOX2, KLF4, MYC, LIN28 and OCT3/4 by the LUMC human iPSC Hotel (https://www.lumc.nl/research/facilities/hipsc-core-facility/).

Pat_ex19fs originated from a 67-year old female who presented with late-onset, chronic ataxia and dystonia, carrying a heterozygous frameshift variant (NM_001127221.1: c.2518_2519insCGGC) in exon 19 of *CACNA1A*. Pat_E668A originated from a 27-year old male who presented a mild, barely progressive ataxia and migraine, and carried a heterozygous missense variant (NM_001127221.1: c.2003A>C) in exon 16 of *CACNA1A*. Both these iPSC lines were reprogrammed from PBMCs with episomal vectors expressing SOX2, KLF4, MYC, LIN28 and OCT3/4 by the Radboudumc Stem Cell Technology Center (SCTC) (https://www.radboudumc.nl/en/research/radboud-technology-centers/stem-cells).

Pat_V1393M originated from a 6-year old female with congenital chronic ataxia and early-onset epileptic encephalopathy with refractory seizures. She carried a heterozygous missense variant (NM_001127221.1: c.4177G>A) in exon 26 of *CACNA1A*. This cell line was reprogrammed from erythroblasts using Sendai virus using the Cytotune reprogramming kit and was made available through the CACNA1A Foundation and the COMBINEDBrain Biorepository. The control cell line, Ctr, (UCSFi001-A, obtained from the Coriell Institute (GM25256, RRID: CVCL_Y803)) was reprogrammed from skin fibroblasts of a 30-year old healthy male.

CRISPR/Cas9 editing of *CACNA1A*

Generation of isogenic cell lines was performed with two different CRISPR/Cas9 strategies: plasmid-based and ribonucleoprotein (RNP) complex-based. Ctr_N390K and Ctr_P1862Pfs*9 iPSCs were generated with the plasmid-based protocol, whereas Rescue_ex19, Rescue_V1393M, Ctr_V1393M and Ctr_R1434Q iPSCs were generated with the RNP complex-based protocol (Table S5). The single guide (sg)RNAs and HDR templates were designed with Benchling (https://www.benchling.com/) (Table S5).

For the plasmid-based protocol, as previously described (Hommersom, et al. 2022), sgRNAs were cloned into the pSpCas9(BB)-2A-Puro (PX459) V2.0 plasmid (a gift from Feng Zhang (RRID:Addgene_62988)) (Ran, et al. 2013). Nucleofection of 800,000 cells with 5 µg plasmid, and if applicable, 4 µM HDR template (Ultramer DNA oligo, IDT) was performed with the 4D-Nucleofector™ System (program CA-137, Lonza). Cells were seeded into a recombinant human laminin LN521 (5 µg/mL; BioLamina, #LN521)-coated 6-well plate in Essential 8™ Flex Basal Medium (Gibco,

#A2858501) supplemented with primocin (0.1 µg/ml; Invivogen, #ant-pm-2) and Revitacell (Gibco, #A2644501). 24 h after nucleofection, the cells were selected with puromycin (0.5 µg/ml; Sigma-Aldrich, #P9620) for 24 h. Surviving colonies were picked and individually transferred into a 96-well plate.

For the RNP complex-based protocol, sgRNAs were ordered as Alt-R crRNAs (IDT), which were annealed to Alt-R tracrRNAs (IDT, #1072533) in nuclease-free duplex buffer (IDT, #11-01-03-01) at 95 °C for 5 min. The resultant sgRNAs were incubated with Alt-R S.p. Cas9 Nuclease V3 (IDT, #1081059) for 20 min at room temperature. Nucleofection of 200,000 or 300,000 cells with 4 µM RNP complex and 4 µM HDR template was performed with the 4D-Nucleofector™ System (program CA-137, Lonza). Cells were seeded into a recombinant human laminin 521-coated 12- or 24-well plate in the presence of Revitacell. 96 h after nucleofection, half of the cells were plated as single cells onto a 6-well plate and half was taken for DNA isolation and Sanger sequencing to check for HDR efficiency. Colonies derived from the single cells were picked and individually transferred into a 96-well plate.

To identify edited clones, DNA was isolated using Proteinase K (1 mg/mL, Thermo Scientific) and amplified by PCR (Table S6). PCR products were purified with Exonuclease I (1.5 U/µL, Thermo Scientific) and FastAP Thermosensitive Alkaline Phosphatase (0.3 U/µL, Thermo Scientific), and sequenced by Sanger sequencing (Figure S3).

Characterization of iPSC lines

Morphology of the induced pluripotent stem cell (iPSC) lines was assessed by bright-field microscopy (Figure S3). Images were taken with an Invitrogen™ EVOS™ XL Core cell imaging system with 4x, 10x and 20x EVOS objectives. DNA isolation for rtTA-*Ngn2*-positive or rtTA-*Ascl1/Dlx2*-positive iPSCs (see below) was done via the QIAamp DNA Mini Kit (Qiagen, #51306). For Rescue_ex19, Ctr_N390K, Ctr_R1434Q, and Ctr_P1862Pfs*9 iPSC lines, off-target analysis was performed by sequencing the top 3 off-target sites of each sgRNA predicted by both Benchling and CRISPOR.(Concordet and Haeussler 2018) These iPSC lines, as well as Pat_ex5del, Pat_ex19fs and Pat_E668A lines were validated for pluripotency markers and trilineage differentiation potential (STEMdiff™ Trilineage Differentiation Kit, STEMCELL Technologies, #05230) by immunocytochemistry after all experiments were performed, within 12 passages after thawing the cell lines. Short tandem repeat (STR) analysis of 16 loci was performed using the AmpFLSTR identifiler PCR amplification kit (Life Technologies). Lastly, copy number variation (CNV)/whole exome sequencing (WES) analysis was carried out for all these cell lines to check for major karyotype abnormalities, after all experiments were performed to ensure genomic integrity throughout the experiment. CNV/WES revealed a 60-65% trisomy 8 in rtTA-*Ngn2*-positive Pat_E668A cells and a chr.7p gain in rtTA-*Ngn2*-positive Pat_ex5del cells. For Pat_V1393M, Rescue_V1393M, and Ctr_V1393M rtTA-*Ngn2*-positive or rtTA-*Ascl1/Dlx2*-positive iPSCs, genomic stability was assessed before experimentation by detection of recurrent genetic abnormalities using the iCS-digital™ PSC test, provided as a service by Stem Genomics (https://www.stemgenomics.com/), which identified a chr.12p gain in the rtTA-*Ngn2*-positive Pat_V1393M cell line. The absence of mycoplasma was tested regularly by the MycoAlert™ PLUS mycoplasma detection kit (Lonza, # LT07-710). All data is available upon request.

Generation of rtTA-*Ngn2*-positive iPSCs

All cell lines in this study were transduced with lentiviral vectors to integrate rtTA (pLV-EF1α>Tet3G:IRES:Neo) and *Ngn2* (pLV[TetOn]-Puro-TRE3G>mNeurog2([NM_009718.3])) transgenes into their genome. 48 h after transduction with both vectors, cells were selected with G418 (25 µg/ml; Sigma- Aldrich, #G8168) and puromycin (0.5 µg/ml), of which concentrations increased over time. Colonies that survived the selection process were cultured in Essential 8™ Flex Basal medium, supplemented with primocin, G418 (50 µg/ml) and puromycin (0.5 µg/ml) on Vitronectin-N (VTN-N; Gibco, #A14700) or Geltrex (Gibco, #A1413302)-coated plates at 37°C/5% CO_2. Cells were passaged with ReLeSR (STEMCELL Technologies, #100-0483), 1-2 times per week when they reached 80-90% confluency and cryopreserved using PSC cryomedium (Gibco, #A2644601).

Generation of rtTA-*Ascl1/Dlx2*-positive iPSCs

24-well plates were precoated with recombinant human laminin LN521. Single cells were generated with TrypLE™ Express (Gibco, #12604021) and 50,000 cells were plated in Essential 8™ Basal medium (Gibco, #A1517001), supplemented with primocin and RevitaCell into one well of a 24-well plate. 24 h after plating, iPSCs were refreshed with Essential 8™ Basal medium supplemented with primocin, and transfected with a piggybac vector PB-*Ascl1-Dlx2* (van Voorst, et al. 2025) and a transposase using Lipofectamine Stem Transfection Reagent (Invitrogen, #STEM00003) in Opti-MEM (Gibco, #31985062). 24 h after transfection medium was refreshed and selection with puromycin was started (0.5 µg/ml), which was increased over time. The iPSCs that survived the selection process were cultured in Essential 8™ Flex Basal medium, supplemented with primocin, and treated with puromycin (0.5 µg/ml) for 24 h during every second or third split.

Neuronal differentiation

Human iPSCs were differentiated into glutamatergic neurons by doxycycline-inducible *Ngn2* overexpression (Zhang, et al. 2013, Frega, et al. 2017) or into GABAergic cortical neurons by doxycycline-inducible *Ascl1* and *Dlx2* overexpression (Yang, et al. 2017, van Voorst, et al. 2025). After at least 2 and maximal 10 passages after thawing, single cells [days *in vitro* (DIV0)] were generated from rtTA-*Ngn2*-positive or rtTA-*Ascl1/Dlx2*-positive iPSCs for neuronal differentiation by incubating with TrypLE™ Express at 37°C/5% CO_2. The iPSCs were resuspended in Essential 8™ Basal medium, supplemented with RevitaCell, primocin and doxycycline (4 µg/mL; Sigma Aldrich, #D9891). The plates were pre-coated with poly-L-ornithine hydrobromide (50 µg/mL; Sigma-Aldrich, #P3655) in borate buffer (50 mM) for 3 h at 37°C/5% CO_2, followed by overnight incubation with human recombinant laminin LN521 at 4°C. At DIV1, culture medium was changed to DMEM/F12 medium (Gibco, #11320074), supplemented with MEM non-essential amino acid solution (Sigma-Aldrich, #M7145), N2 (Gibco, #17502048), recombinant human BDNF (10 ng/mL; PromoCell, #C-6621), NT3 (10 ng/mL; PromoCell, #C-66425), doxycycline and mouse laminin from Engelbreth-Holm-Swarm sarcoma (0.2 µg/mL; Sigma-Aldrich, #L2020). To support neuronal maturation and viability, rat embryonic astrocytes were added to the neuronal culture in a 1:1 ratio/well at DIV2. At DIV3, the medium was changed to Neurobasal medium (Gibco, #21103049) supplemented with B-27 (20 µg/mL; Gibco, #17504001), primocin, GlutaMAX (10 µg/mL; Gibco, #35050038), BDNF, NT3 and doxycycline. Furthermore, cytosine β-D-arabinofuranoside hydrochloride (Ara-C) (2 µM; Sigma-Aldrich, #C6645) was added once at DIV3, to remove any proliferating cells from the culture. From DIV6 to DIV9, half of the medium was refreshed every other day with fresh neurobasal medium supplemented with B-27, primocin, GlutaMAX, BDNF, NT3 and doxycycline. From DIV10 onwards till the end of the neuronal culture, every other day half of the medium was refreshed with neurobasal medium supplemented with B-27, primocin, GlutaMAX, BDNF, NT3 and additional 2.5% fetal bovine serum (FBS; Sigma-Aldrich, #F7524), to support astrocyte viability. Throughout the entire differentiation process, the cultures were incubated at 37°C/5% CO_2.

Single-cell electrophysiology

Coverslips with DIV42 neurons were placed in a recording chamber on the stage of an Olympus BX51WI upright microscope (Olympus Life Science), equipped with infrared differential interference contrast optics, an Olympus LUMPlanFL N 60x water-immersion objective (Olympus Life Science), and a kappa MXC 200 camera system (Kappa optronics GmbH) for visualization. The recording chamber was continuously perfused with oxygenated (95% O_2/5% CO_2) artificial cerebrospinal fluid (aCSF) at 32°C containing (in mM): 124 NaCl, 1.25 NaH_2PO_4, 3 KCl, 26 $NaHCO_3$, 11 Glucose, 2 $CaCl_2$, 1 $MgCl_2$. Patch pipettes (ID 0.86 mm, OD1.05 mm, resistance 6–8 MΩ) were pulled from borosilicate glass with filament and fire-polished ends (Science Products GmbH) using the Narishige PC-10 micropipette puller. These pipettes were filled with a potassium-based intracellular solution containing (in mM): 130 K-Gluconate, 5 KCl, 10 HEPES, 2.5 $MgCl_2$, 4 Na_2-ATP, 0.4 Na_3-GTP, 10 Na-phosphocreatine, 0.6 EGTA (with pH adjusted to 7.25 and osmolarity to 290 mOsmol). We acquired

recordings using a Digidata 1140A digitizer and a Multiclamp 700B amplifier (Molecular Devices), with a sampling rate set at 20 kHz and a lowpass 1kHz filter during recording. We did not correct for liquid junction potential. Recordings were omitted from analysis if series resistance was above 25 MΩ or when the recording reached below a 10:1 ratio of membrane resistance to series resistance. Analysis of passive membrane properties was conducted in voltage clamp mode at a holding potential of -60 mV. Resting membrane potential (RMP), was determined in current clamp mode directly after reaching whole-cell configuration. Consequently, active intrinsic properties were measured with a stepwise current injection protocol ranging from -30 pA to +70 pA. Intrinsic properties were analysed with the Action Potential Search algorithm of Clampfit 11.2 (Molecular devices). We assessed the properties of every first elicited action potential. Where applicable, measurements were taken relative to the threshold of each action potential. Rise and decay time were measured between 10 and 90% of the threshold-relative amplitude.

Immunocytochemistry and image analysis

Cells were fixed in 4% paraformaldehyde (PFA) containing 4% sucrose for 10 minutes at room temperature (RT), followed by three 5-minute washes in PBS. For permeabilization and blocking, cells were incubated in PBS supplemented with 10% normal goat serum (NGS) and 0.2% Triton X-100 for 30 minutes at RT. Primary antibodies were diluted in PBS containing 5% NGS and 0.1% Triton X-100 and applied overnight at 4 °C. The following primary antibodies were used: guinea pig anti-MAP2 (1:1000; Synaptic Systems, #188004) for quantification of GABAergic neurons, and guinea anti-VGAT (1:200; Synaptic Systems, #131308) and chicken anti-Gephyrin (1:500; Synaptic Systems, #147009) for GABAergic synapse quantification. After primary incubation, cells were washed three times for 5 minutes with PBS and incubated for 1 hour at RT with secondary antibodies (1:1000) and FluoTag-X2 anti-TagFP Atto 488 (1:200; NanoTag Biotechnologies, #N0502-At488-L) for the quantification of GABAergic neurons, diluted in PBS containing 5% NGS and 0.1% Triton X-100. Cells were then washed three times for 5 minutes with PBS. Coverslips were briefly rinsed in ultrapure water and mounted in DAKO mounting medium.

The percentage of GABAergic neurons was determined from images acquired using a Zeiss Axio Imager Z1 microscope. The proportion of TagFP-positive neurons was calculated relative to the total number of MAP2-positive neurons within each field of view, and averaged across images for each coverslip. These mean values were subsequently used for data visualization. For GABAergic synapse quantification, images were acquired using the same microscope. Regions of interest (ROIs) were manually traced along the proximal dendrites of neurons in ImageJ. Synaptic puncta were identified and quantified using the SynBot plugin (Savage, et al. 2024) with the Ilastik-based thresholding method to segment presynaptic VGAT and postsynaptic Gephyrin puncta. Co-localization analysis was then performed to identify putative functional synapses. The number of functional synaptic puncta within each dendritic ROI was quantified using a custom ImageJ macro, normalized to 10 µm of dendrite length, and averaged across dendrites for each neuron. These mean values were subsequently used for data visualization.

Flow cytometry and analysis

GABAergic and glutamatergic iPSCs were plated at a 1:1 ratio on 6-well plates, which were then co-cultured with rodent astrocytes at a neurons:astrocyte ratio of 1:1 under the same culture conditions as other experiments. At DIV49, cells were dissociated using Accutase (Sigma, #A6964) and collected into tubes. Cells were resuspended either in PBS (unstained control) or in PBS containing LIVE/DEAD™ Fixable Near-IR Viability Dye (780) (1:4000; Thermo Fisher, #L34994) and incubated for 30 min at 4 °C protected from light. Cells were washed with flow buffer (PBS supplemented with 1% BSA) and fixed with the Fix solution from the Fix&Perm kit (Nordic-MuBio) for 15 min at room temperature (RT). Fixed cells were washed with flow buffer. Unstained controls were stored at 4 °C until use, while LIVE/DEAD-stained cells were incubated in permeabilization solution (Fix&Perm kit,

Nordic-MuBio) containing CD90 (Thy-1) PE/Dazzle 594 (1:50; BioLegend, #328134) and FluoTag-X2 anti-TagFP Atto 488 (1:200; NanoTag Biotechnologies, #N0502-At488-L). Samples were incubated for 30 min at 4 °C in the dark, washed twice with flow buffer, and resuspended for acquisition. Data were acquired on a Gallios flow cytometer (Beckman Coulter) using calibrated instrument settings and appropriate compensation controls.

Flow cytometry data were analysed using Kaluza 2.1 software (Beckman Coulter). Cells were first gated based on forward scatter area (FSC-A) and side scatter area (SSC-A) to exclude debris. Doublets were then removed by comparing FSC-A vs FSC-H and SSC-A vs SSC-H parameters. From the single-cell population, viable cells were identified as those with low fluorescence in the LIVE/DEAD channel, as the dye penetrates and labels intracellular amines only in cells with compromised membranes. Within the live single-cell population, CD90-positive events were classified as neurons, as astrocytes were confirmed to be CD90-negative (data not shown). The proportion of BFP-positive (GABAergic) neurons was then quantified within this gated population. Unstained control samples were used to define background autofluorescence in each channel and to set thresholds for positive staining.

Supplemental references

Concordet J-P, Haeussler M. CRISPOR: intuitive guide selection for CRISPR/Cas9 genome editing experiments and screens. *Nucleic Acids Research*. 2018;46(W1):W242-W245. doi:10.1093/nar/gky354

Frega M, van Gestel SH, Linda K, van der Raadt J, Keller J, Van Rhijn JR, Schubert D, Albers CA, Nadif Kasri N. Rapid Neuronal Differentiation of Induced Pluripotent Stem Cells for Measuring Network Activity on Micro-electrode Arrays. *J Vis Exp*. Jan 8 2017;(119)doi:10.3791/54900

Hommersom MP, Bijnagte-Schoenmaker C, Albert S, van de Warrenburg BPC, Nadif Kasri N, van Bokhoven H. Generation of induced pluripotent stem cell lines carrying monoallelic (UCSFi001-A-60) or biallelic (UCSFi001-A-61; UCSFi001-A-62) frameshift variants in CACNA1A using CRISPR/Cas9. *Stem Cell Res*. May 2022;61:102730. doi:10.1016/j.scr.2022.102730

Ran FA, Hsu PD, Wright J, Agarwala V, Scott DA, Zhang F. Genome engineering using the CRISPR-Cas9 system. *Nat Protoc*. Nov 2013;8(11):2281-2308. doi:10.1038/nprot.2013.143

Savage JT, Ramirez J, Risher WC, Wang Y, Irala D, Eroglu C. SynBot: An open-source image analysis software for automated quantification of synapses. *bioRxiv*. 2024:2023.06.26.546578. doi:10.1101/2023.06.26.546578

van Voorst TW, van Boven MA, Marinus KI, Colón-Mercado JM, Schretzmeir J, Haag C, Toonen RF, Koopmans F, Ward ME, Smit AB*, et al*. One-step induction of human GABAergic neurons promotes presynaptic development & synapse maturation. *bioRxiv*. 2025:2025.06.30.662293. doi:10.1101/2025.06.30.662293

Yang N, Chanda S, Marro S, Ng Y-H, Janas JA, Haag D, Ang CE, Tang Y, Flores Q, Mall M*, et al*. Generation of pure GABAergic neurons by transcription factor programming. *Nature Methods*. 2017/06/01 2017;14(6):621-628. doi:10.1038/nmeth.4291

Zhang Y, Pak C, Han Y, Ahlenius H, Zhang Z, Chanda S, Marro S, Patzke C, Acuna C, Covy J*, et al*. Rapid single-step induction of functional neurons from human pluripotent stem cells. *Neuron*. Jun 5 2013;78(5):785-98. doi:10.1016/j.neuron.2013.05.029
