# Supplementary material for: Human neuronal networks on micro-electrode arrays as a tool to assess genotype-phenotype correlation in CACNA1A-related disorders
Source: Stem Cell Reports. 2026 Jan 22;21(2):102783. doi: 10.1016/j.stemcr.2025.102783 (PMC12903092; doi:10.1016/j.stemcr.2025.102783)
Supplement: Document S2. Article plus supplemental information [file mmc3.pdf]

STEM CELL REPORTS
Article

OPEN ACCESS

Human neuronal networks on micro-electrode arrays as a tool to assess genotype-phenotype correlation in *CACNA1A*-related disorders

Marina P. Hommersom,[1] Sofía Puvogel,[1] Nicky Scheefhals,[1] Eleonora Carpentiero,[1] Marga Bouma,[1] Ellen van Beusekom,[1] Lieke Dillen,[1] Bart P.C. van de Warrenburg,[2,4] Nael Nadif Kasri,[1,3,4] and Hans van Bokhoven[1,3,4,5,*]

[1]Department of Human Genetics, Radboud University Medical Center, Donders Institute for Brain, Cognition, and Behaviour, Nijmegen 6500 HB, the Netherlands
[2]Department of Neurology, Radboud University Medical Center, Donders Institute for Brain, Cognition, and Behaviour, Nijmegen 6500 HB, the Netherlands
[3]Department of Cognitive Neurosciences, Radboud University Medical Center, Donders Institute for Brain, Cognition, and Behaviour, Nijmegen 6500 HB, the Netherlands
[4]These authors contributed equally
[5]Lead contact
*Correspondence: hans.vanbokhoven@radboudumc.nl
https://doi.org/10.1016/j.stemcr.2025.102783

SUMMARY

CACNA1A-related disorders constitute a diverse group of neurological conditions, including ataxia, migraine, and epilepsy. Despite extensive genetic studies, clear genotype-phenotype correlations remain elusive. Moreover, next-generation sequencing has identified many variants of uncertain significance (VUS). Here, we leveraged patient-derived and CRISPR-Cas9-engineered human neuronal networks to explore relationships between *CACNA1A* variants and neurophysiological activity. *CACNA1A* haploinsufficiency induced subtle alterations in glutamatergic network activity, whereas missense variants had a more pronounced effect on overall network function. Network fingerprints were most affected from patients where ataxia co-occurred with migraine or epilepsy. Furthermore, we analyzed the impact of CRISPR-Cas9-induced VUS on network developmental trajectories. Although functional changes could not be directly linked to clinical phenotypes, all tested variants induced measurable alterations in neuronal network function, supporting their classification as likely pathogenic. These findings highlight the potential of human neuronal networks as a translational model for evaluating *CACNA1A* variant effects and improving clinical variant interpretation.

INTRODUCTION

CACNA1A-related disorders encompass a heterogeneous group of neurological conditions, primarily characterized by episodic or progressive ataxia, hemiplegic migraine, and various forms of epilepsy (Indelicato and Boesch 2021; Hommersom et al., 2022). The gene *CACNA1A* encodes the pore-forming subunit of the P/Q-type voltage-gated calcium channel, $Ca_V2.1$, which plays a critical role in neuronal function. $Ca_V2.1$ is predominantly located at presynaptic terminals, where it regulates neurotransmitter release (Llinás et al., 1989; Turner et al., 1992; Uchitel et al., 1992; Luebke et al., 1993; Takahashi and Momiyama 1993; Wheeler et al., 1994; Lübbert et al., 2019), but it is also present in dendrites and soma contributing to the control of neuronal excitability (Yu et al., 2010). The channel's function is fine-tuned by its position in the plasma membrane, its kinetic properties, and voltage dependence, which are influenced by the intrinsic properties of specific splice isoforms and their interactions with various auxiliary subunits (Soong et al., 2002; Chaudhuri et al., 2004; Davies et al., 2007; Buraei and Yang 2010; Thalhammer et al., 2017). Given this complexity, it is unsurprising that *CACNA1A* variants can have wide-ranging molecular, cellular, and systemic effects.

Functional analysis of overexpressed *CACNA1A* variants in HEK293T cells has provided valuable insights into specific effects of genetic variants on $Ca_V2.1$ channel function (Guida et al., 2001; Cuenca-León et al., 2009; Garza-López, et al. 2012, 2013; Condliffe et al., 2013; Ohmori et al., 2013; Bahamonde et al., 2016; Huang et al., 2019; Jiang et al., 2019; Gandini et al., 2021). However, these models lack the physiological complexity and cellular context of the human brain. They do not capture the influence of genetic background, alternative splicing, or auxiliary subunit interaction, limiting their relevance for understanding the full spectrum of *CACNA1A*-related phenotypes.

In contrast, human-derived neuronal networks offer a distinct advantage for modelling neurological disorders, as they maintain both the human genetic context and physiological relevance. Another direct advantage of using patient-derived cells is that they allow to make direct connection between experimental data and a specific constellation of clinical features. Advances in CRISPR-Cas9 gene-editing techniques enable us to precisely investigate the effects of specific variants by utilizing isogenic cell lines. Furthermore, electrophysiological techniques allow for detailed examination of neuronal (network) activity, shedding light on how human variants affect single-cell or network-level output. Previously, we have demonstrated that control networks

© 2025 The Authors.
Published by Elsevier Inc. on behalf of International Society for Stem Cell Research.
This is an open access article under the CC BY license (http://creativecommons.org/licenses/by/4.0/).

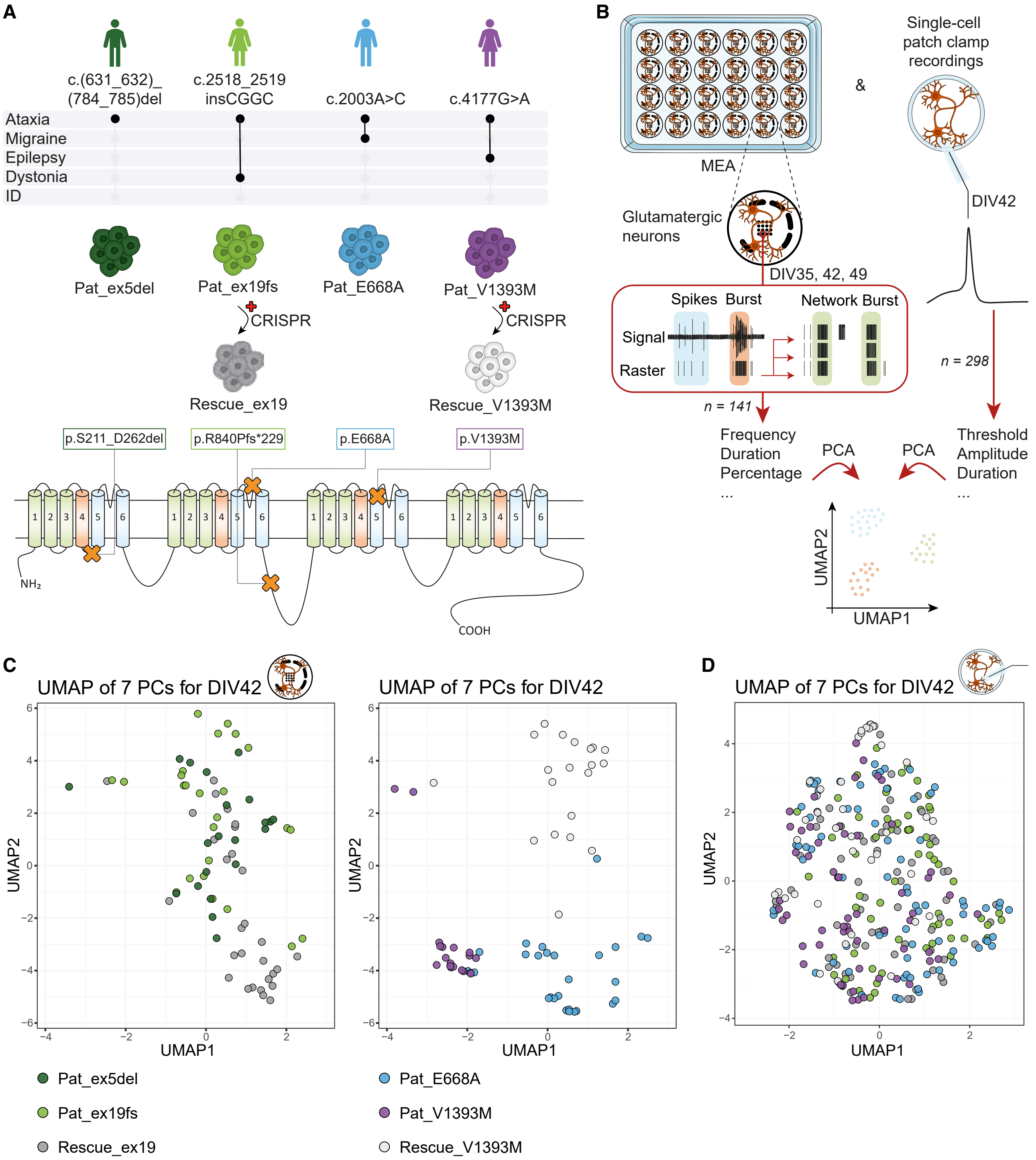

Figure 1. Clustering of *CACNA1A* patient-derived glutamatergic neuronal networks based on their electrophysiological activity over late development

(A) Schematic overview of the clinical phenotypes of *CACNA1A* patients included in this study with their genetic variants (NM_00127221). Two patient-derived induced pluripotent stem cell (iPSC) lines were genetically edited with CRISPR-Cas9 to generate isogenic controls. Genetic variants are also represented as orange crosses in a schematic representation of Cav2.1. Transmembrane domains are shown in green, voltage-sensitive domains in red, and pore-forming domains in blue.

(legend continued on next page)

exhibit robust and highly similar functional properties (Mossink et al., 2021) and that disturbances in neuronal network activity are characteristic for various neurodevelopmental disorders (Frega et al., 2019; Klein Gunnewiek et al., 2020; Mossink et al., 2021; Linda et al., 2022; Wang et al., 2022; van Hugte et al., 2023). In our recent work, we demonstrated that *CACNA1A* haploinsufficiency in induced pluripotent stem cell (iPSC)-derived neurons results in altered network synchronization that is accompanied by reduced synaptic function and increased intrinsic excitability (Hommersom et al., 2024). However, a key limitation of this study was that we relied solely on CRISPR-Cas9-engineered control iPSCs that prevented us from linking our findings to specific clinical manifestations.

To address this issue, we here use multiple patient iPSC-derived neuronal networks consisting of glutamatergic only or glutamatergic and GABAergic neurons to characterize alterations in neurophysiological activity. We measured their electrical activity, both through patch clamp recordings to study single-cell level activity and micro-electrode arrays (MEAs) to study neuronal network-level activity. We hypothesized that neuronal network functioning could discriminate the different iPSC-derived networks based on the different variant types and/or different phenotypes. We show that specific parameters could be correlated to $Ca_V2.1$ gain-of-function with ataxia and epilepsy as a clinical phenotype, which were rescued by Ca_V2 inhibitors. Moreover, we demonstrate an increased inhibitory drive in GABAergic neurons carrying this gain-of-function variant. Lastly, we explored the classification of variants of uncertain significance (VUS) by leveraging the neuronal network phenotypes, hereafter referred to as network fingerprints.

RESULTS

CACNA1A patient-derived glutamatergic neuronal networks show variant type-specific network fingerprints

In order to characterize the neuronal activity of different *CACNA1A* patient-derived neuronal networks, we used iPSC lines of four different patients with diverse clinical phenotypes and genetic variants (Figure 1A). All patients showed episodic or chronic ataxia, either isolated or combined with dystonia, migraine, or epilepsy. The patient-derived iPSC lines harboured different genetic variants, including an exon deletion (Pat_ex5del), a frameshift variant (Pat_ex19fs), and two missense variants, of which one was predicted loss-of-function (Pat_E668A) and one gain-of-function (Pat_V1393M) (Jiang et al., 2019). We generated two isogenic control lines, by rescuing the frameshift variant of Pat_ex19fs (Rescue_ex19) and the missense variant of Pat_V1393M (Rescue_V1393M). We then differentiated these iPSC lines toward glutamatergic neurons via doxycycline-induced *Ngn2* overexpression and recorded their neuronal network activity using MEAs over late development, when all networks showed synchronization in the form of network bursts (Figure 1B). For all networks, except for Pat_ex5del, we also recorded their intrinsic electrophysiological activity through single-cell patch-clamp recordings (Figure 1B). To get a first impression on how the different neuronal network activities would compare to each other, we performed a principal component analysis (PCA) on all available parameters. For 39 MEA parameters recorded over 3 days *in vitro* (DIVs), we plotted the results of seven principal components (PCs) in a uniform manifold approximation and projection (UMAP) space (Figures 1C and S1A). Patient lines harbouring missense variants that are associated with ataxia combined with migraine and epilepsy, clustered further apart from the (isogenic) control lines than patient lines with full loss-of-function alleles that were associated with ataxia and dystonia. For eleven single-cell electrophysiological properties recorded at DIV42, we applied the same approach but observed no clustering (Figure 1D). This suggests that this multiparametric approach cannot be used to discriminate different patient-derived networks with their single-cell intrinsic electrophysiological activity, whereas changes were uncovered between the different networks using single parameters (Figure S1B).

(B) Schematic overview of neuronal differentiation toward recording network activity on micro-electrode arrays (MEAs) at days *in vitro* (DIV)35, 42, and 49 and recording single-cell electrophysiological activity at DIV42. Principal component analysis (PCA) was performed on parameters extracted from these recordings, and seven principal components (PCs) were plotted in uniform manifold approximation and projection (UMAP) space.
(C) UMAP analysis on seven PCs obtained from network activity parameters at DIV42 for all iPSC-derived networks indicated in (A). $n = 21/3$ for Pat_ex5del, $n = 25/4$ for Pat_ex19fs, $n = 29/4$ for Rescue_ex19, $n = 27/4$ for Pat_E668A, $n = 19/3$ for Pat_V1393M, and $n = 20/2$ for Rescue_V1393M.
(D) UMAP analysis on seven PCs obtained from single-cell intrinsic properties at DIV42 for all iPSC-derived indicated in (A), except for Pat_ex5del. $n = 68/8$ for Pat_ex19fs, $n = 63/7$ for Rescue_ex19, $n = 73/5$ for Pat_E668A, $n = 57/5$ for Pat_V1393M, and $n = 41/2$ for Rescue_V1393M.
See also Figure S1.

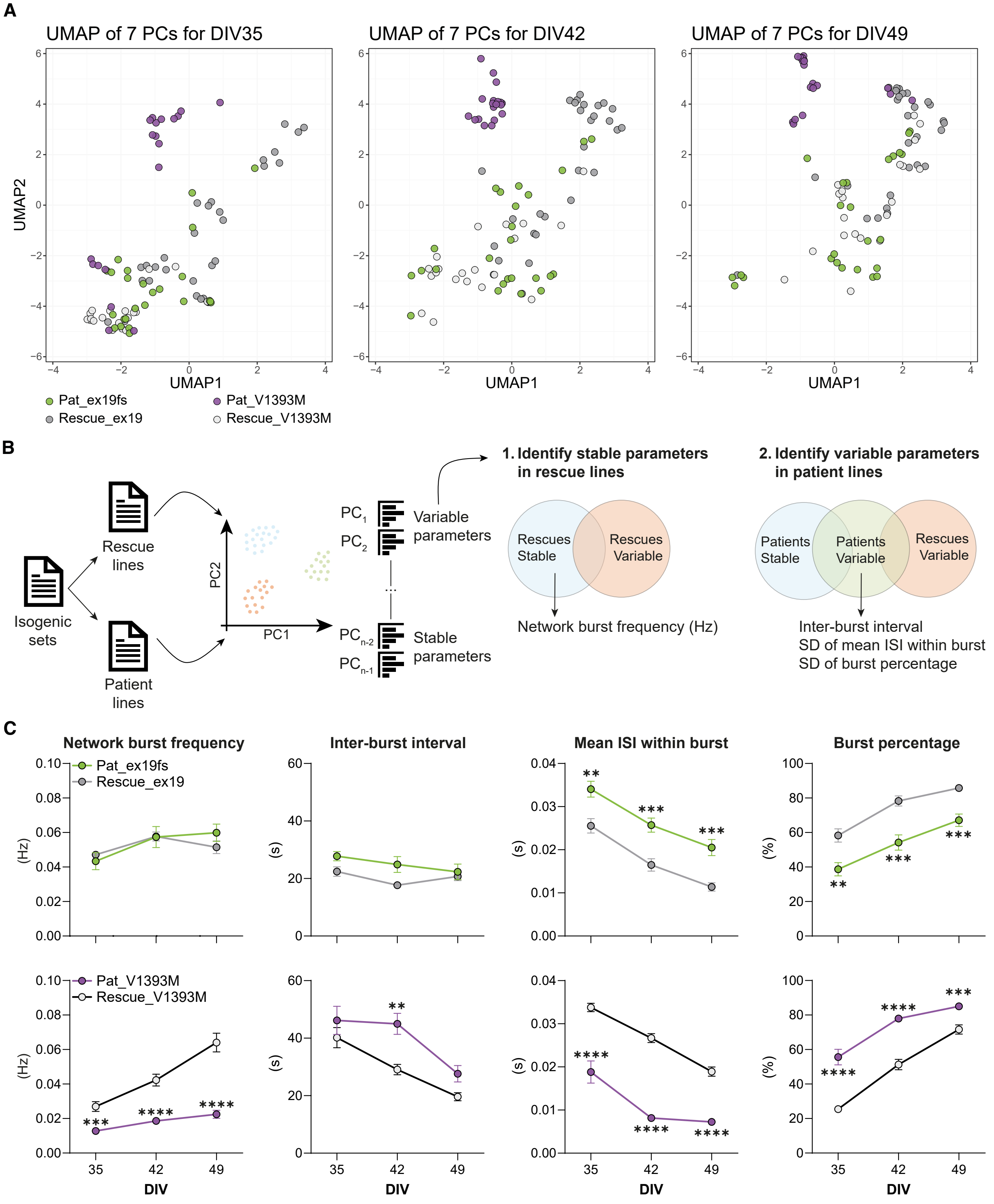

Figure 2. Identification of variant- or phenotype-specific developmental changes in network activity parameters

(A) Uniform manifold approximation and projection (UMAP) analysis on seven principal components (PCs) obtained from network activity parameters at days *in vitro* (DIV)35, 42, and 49 for the isogenic iPSC-derived networks indicated in Figure 1A.

(legend continued on next page)

To narrow down on the parameters that could describe the different variant types (*null* variants vs. missense variants), we focused on the isogenic pairs (Figure 2A). We first stratified the dataset into patient-derived networks and isogenic control (rescue) networks (Figure 2B) and performed PCA on these separate datasets. By selecting ten parameters contributing the most to each PC, we identified those responsible for the greatest variance, i.e., variable parameters (belonging to PC1 and PC2), as well as those contributing the least variance, i.e., stable parameters (belonging to the last two PCs). To minimize the inclusion of extraneous variables, we applied two filtering strategies. First, we identified parameters that remained stable across both rescue networks (Figure 2B). Next, by overlaying the 20 variable parameters with the 20 stable parameters, we extracted those that contributed exclusively to the last two PCs and not to the first two PCs (Table S1). This approach yielded a key parameter: network burst frequency, defined by the number of network bursts over a 5-min period. Indeed, the network burst frequency of both rescue lines fell within similar ranges (Figure 2C). While we did not observe changes in the network burst frequency for the frameshift variant, the neurons carrying the gain-of-function missense variant (Pat_V1393M) consistently showed a reduced network burst frequency throughout development (Figure 2C).

To further refine our analysis, we focused on parameters that varied in patient networks but not in rescue networks (Figure 2B). This approach identified three key parameters: inter-burst interval (IBI), the standard deviation (SD) of the mean inter-spike interval (ISI) within bursts, and the SD of burst percentage. In isogenic control networks, the IBI was either stable or decreased over development (Figure 2C). While Pat_ex19fs did not show significant differences, Pat_V1393M displayed longer IBI at DIV42. For both the mean ISI within burst, reflecting the compactness of spikes within a burst, and the burst percentage, representing the percentage of spikes that occur within bursts, Pat_ex19fs and Pat_V1393M showed opposite trends compared to their isogenic controls (Figure 2C). Pat_ex19fs exhibited a higher mean ISI within burst and a lower burst percentage than Rescue_ex19, whereas Pat_V1393M showed the opposite trend compared to Rescue_V1393M. Notably, these parameters also varied within the rescue lines. This suggests that rescuing a variant does not necessarily restore all network parameters to control levels.

We then compared both full loss-of-function variants and both missense variants to each other and the isogenic controls (Figure S1C). Interestingly, Pat_ex5del and Pat_ex19fs exhibited very similar network features, and both showed an increased mean ISI within burst and lower burst percentage compared to Rescue_ex19. In contrast, Pat_V1393M and Pat_E668A showed more distinct features, particularly in network burst frequency trajectories and the IBI, yet demonstrated comparable mean ISI within burst and burst percentage. This suggests that these two parameters are particularly informative for assessing variant-specific effects relative to the corresponding isogenic control.

In summary, our results suggest that full loss-of-function alleles induce subtle alterations in developmental network activity, whereas missense variants have a more pronounced impact on developmental trajectories. Notably, while network fingerprints remain relatively similar in cases of dystonia co-occurring with ataxia (Pat_ex19fs), the presence of migraine or epilepsy seems to be associated with significant shifts in the network fingerprint (Pat_E668A and Pat_V1393M).

Cav2.1 gain-of-function leads to fragmented synchronization in glutamatergic neuronal networks

We previously characterized the network fingerprint of *CACNA1A* haploinsufficient networks (Hommersom et al., 2024). To further investigate the effects of a missense variant, we introduced the predicted gain-of-function p.(V1393M) variant into a control cell line. We focused on DIV49 (Figure 3A), the time point at which the most pronounced differences between isogenic pairs were observed (Figure 2C). By applying the same PCA-based approaches as before (Table S2), we found that p.(V1393M) networks (Pat_V1393M and Ctr_V1393M) exhibited fragmented bursts, a feature absent in control networks (control and Rescue_V1393M) (Figures 3A, 3B, and 3D). This is interesting as fragmented bursts have been linked to increased calcium availability in the presynapse (Pradeepan et al., 2024), and we previously classified fragmented bursts as seizurogenic activity in control networks upon treatment with proconvulsive compounds (van Hugte et al., 2023). As in previous analyses, we found that the network burst frequency remained stable across all conditions, whereas network burst percentage, IBI, and IBI coefficient of variation (CoV) were variable in

(B) Schematic overview of analysis steps taken to isolate network activity parameters.
(C) Network activity parameters over DIV35, 42, and 49 for the patient and isogenic rescue lines, including network burst frequency, inter-burst interval, mean inter-spike interval (ISI) within burst, and burst percentage. n = 25/4 for Pat_ex19fs, n = 29/4 for Rescue_ex19, n = 19/3 for Pat_V1393M, and n = 20/2 for Rescue_V1393M. Data are represented as mean ± standard error of the mean (SEM). $^{**}p < 0.01$, $^{***}p < 0.001$, $^{****}p < 0.0001$, two-way ANOVA with mixed-effects model if there are missing values, followed by Šídák's test for multiple comparisons.
See also Figure S1; Table S1.

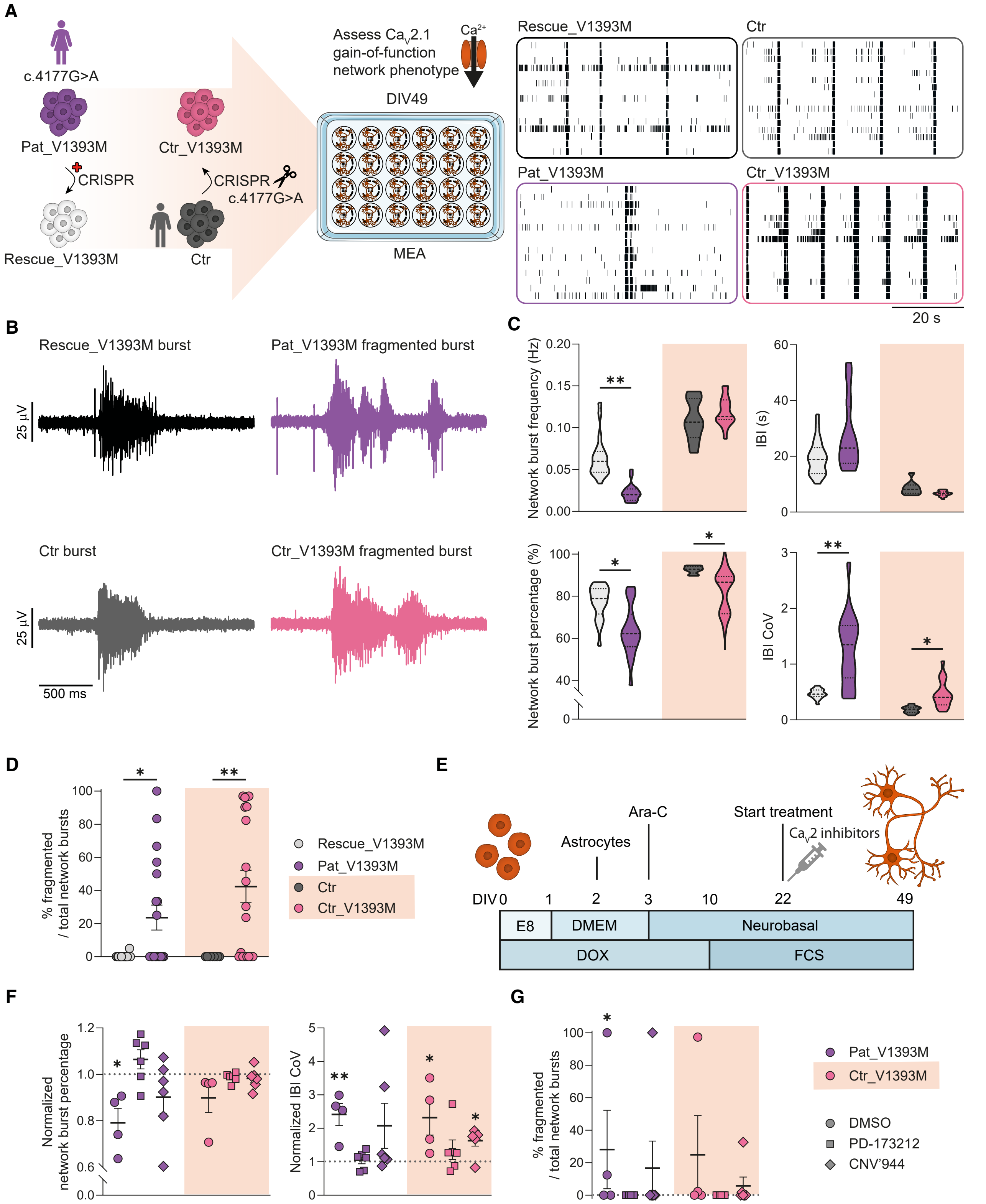

(legend on next page)

p.(V1393M) but not in the control networks. Compared to their respective isogenic controls, Pat_V1393M networks showed a decrease in network burst frequency (Figure 3C), suggesting that human genetic context influences the expressed network fingerprint. While IBI itself was not significantly altered, both IBI CoV and network burst percentage were increased in Pat_V1393M and Ctr_V1393M networks compared to their isogenic controls, indicating that these parameters are specifically modulated by the p.(V1393M) variant.

Since the p.(V1393M) variant has been shown to result in gain-of-function of $Ca_V2.1$ (Jiang et al., 2019), we explored whether these network alterations could be rescued by treating the networks with Ca_V2 inhibitors, PD-173212 (Okada et al., 2021), and CNV'944 (Figure 3E). PD-173212 treatment restored both IBI CoV and network burst percentage to control levels (Figure 3F), while also eliminating fragmented bursts in p.(V1393M) neurons (Figure 3G). CNV'944 treatment reduced the percentage of fragmented bursts (Figure 3G), though it did not fully restore IBI CoV in Ctr_V1393M neurons (Figure 3F).

Taken together, these findings demonstrate that network burst fragmentation, IBI CoV, and network burst percentage are specifically modulated by the p.(V1393M) missense variant in *CACNA1A*, independent of the genetic background. Furthermore, these parameters may be linked to clinical phenotypes such as chronic ataxia and epilepsy (van Hugte et al., 2023).

Cav2.1 gain-of-function leads to an early increased inhibitory drive in GABAergic neurons

CACNA1A is not only essential for glutamatergic neurotransmission but also plays a prominent role in GABAergic neurons (Rossignol et al., 2013; Lupien-Meilleur et al., 2021; Singh et al., 2023), potentially contributing to the coordination between excitation (E) and inhibition (I). To investigate whether *CACNA1A* variants in GABAergic neurons contribute to shifts in E/I coordination, we differentiated Ctr, Ctr_V1393M, Rescue_V1393M, and Pat_V1393M toward GABAergic neurons, via overexpression of *Ascl1* and *Dlx2* (Figure 4A). This recently published protocol efficiently yields pure GABAergic neurons (van Voorst et al., 2025). We then co-cultured these GABAergic neurons with glutamatergic neurons at a 1:1 ratio, resulting in functional E/I networks with an approximate 60:40 glutamatergic-to-GABAergic composition, determined by immunocytochemistry and flow cytometry (Figures S2A–S2C). Within these co-cultures, we consistently observed the formation of functional inhibitory synapses, as evidenced by the colocalization of presynaptic VGAT and postsynaptic gephyrin (Figures S2D and S2E).

To specifically assess the effect of the p.(V1393M) variant on GABAergic neuronal output while maintaining a consistent glutamatergic population, we co-cultured Ctr glutamatergic neurons with GABAergic neurons from Ctr, Ctr_V1393M, Rescue_V1393M, and Pat_V1393M lines (Figure 4B). We then recorded spontaneous network activity

Figure 3. Specific network parameters of p.(V1393M) neuronal networks are rescued by Cav2 inhibitors
(A) Schematic overview of the two isogenic p.(V1393M) pairs that were differentiated toward glutamatergic neurons and recorded on micro-electrode array (MEA) including representative rasterplots (60 s) of network activity of the indicated neuronal networks at days *in vitro* (DIV)49.
(B) Representative electrode bursts (2 s) of Rescue_V1393M and Ctr neuronal networks showing a burst, and Pat_V1393M and Ctr_V1393M neuronal networks showing fragmented synchronization.
(C) Quantification of network activity parameters at DIV49 for isogenic control and p.(V1393M) lines, including network burst frequency, inter-burst interval (IBI), network burst percentage, and IBI coefficient of variation (CoV). n = 20/3 for Rescue_V1393M, n = 19/3 for Pat_V1393M, n = 9/3 for Ctr, and n = 19/3 for Ctr_V1393M. Dashed line represents the median, dotted line represents the quartiles. $^*p < 0.05$, $^{**}p < 0.01$, Kruskal-Wallis test with Dunn's test for multiple comparisons.
(D) Quantification of the percentage fragmented over total network bursts. n = 20/3 for Rescue_V1393M, n = 19/3 for Pat_V1393M, n = 9/3 for Ctr, and n = 19/3 for Ctr_V1393M. Data are represented as mean ± standard error of the mean (SEM). $^*p < 0.05$, $^{**}p < 0.01$, Kruskal-Wallis test with Dunn's test for multiple comparisons.
(E) Schematic overview of treatment strategy of p.(V1393M) networks with Cav2 inhibitors. Dimethyl sulfoxide (DMSO) was used as a vehicle.
(F) Quantification of normalized network parameters including network burst percentage and IBI CoV upon treatment with vehicle (DMSO; 0.05%) or Cav2 inhibitors, PD-173212 (500 nM) or CNV'944 (30 μM). Parameters were normalized against the respective control values. n = 4/2 for Pat_V1393M + DMSO, n = 6/2 for Pat_V1393M + PD-173212, n = 6/2 for Pat_V1393M + CNV'944, n = 4/2 for Ctr_V1393M + DMSO, n = 6/2 for Ctr_V1393M + PD-173212, and n = 6/2 for Ctr_V1393M + CNV'944. Data are represented as mean ± SEM. $^*p < 0.05$, $^{**}p < 0.01$, Kruskal-Wallis test with Dunn's test for multiple comparisons.
(G) Quantification of the percentage fragmented over total network bursts upon treatment with vehicle (DMSO; 0.05%) or Cav2 inhibitors, PD-173212 (500 nM) or CNV'944 (30 μM). n = 4/2 for Pat_V1393M + DMSO, n = 6/2 for Pat_V1393M + PD-173212, n = 6/2 for Pat_V1393M + CNV'944, n = 4/2 for Ctr_V1393M + DMSO, n = 6/2 for Ctr_V1393M + PD-173212, and n = 6/2 for Ctr_V1393M + CNV'944. Data represented as mean ± SEM. $^*p < 0.05$, Kruskal-Wallis test with Dunn's test for multiple comparisons.
See also Table S2.

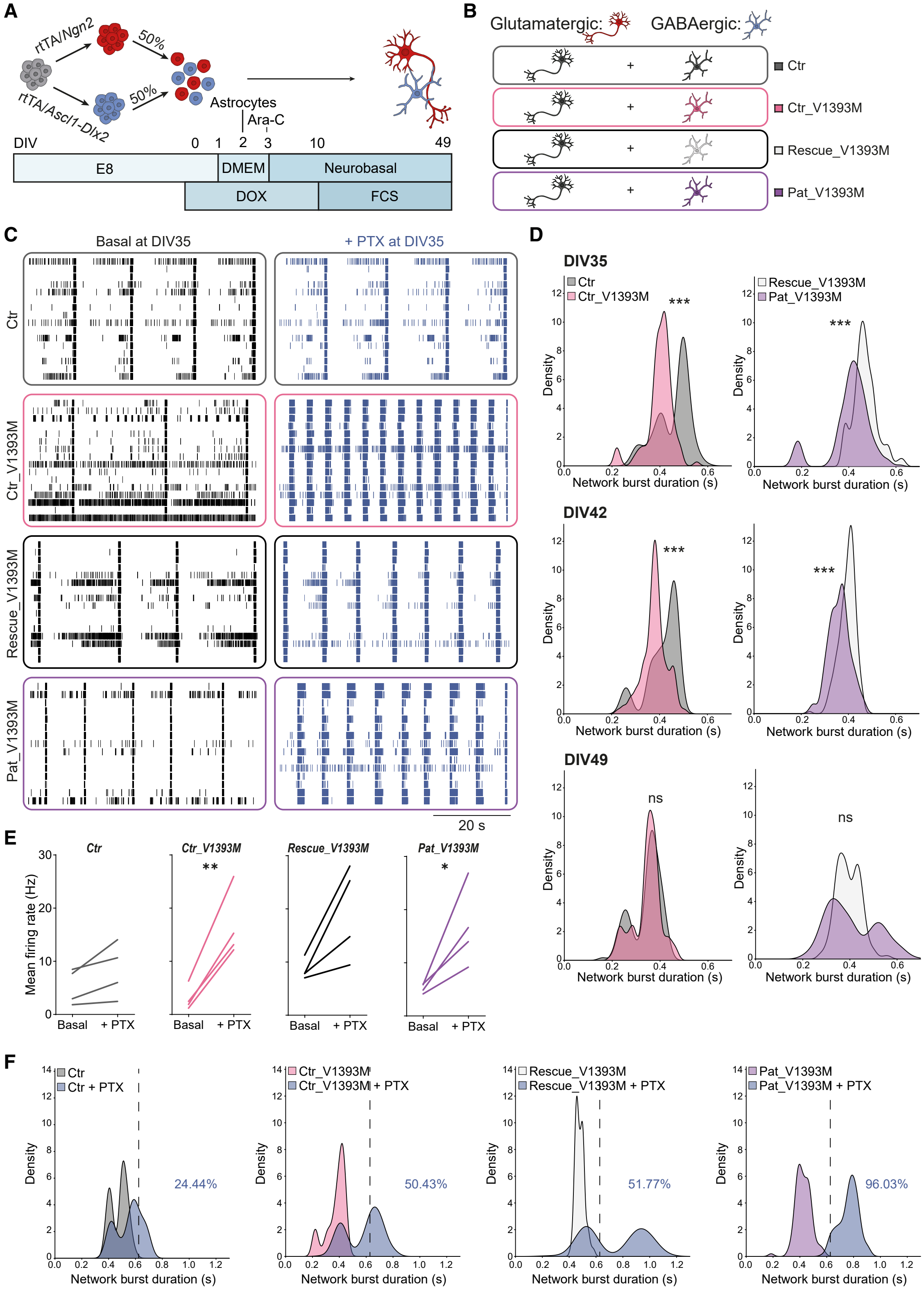

(legend on next page)

on MEAs at DIV35, DIV42, and DIV49 (Figures 4C and 4D). To better capture the heterogeneity in network burst durations and avoid over-reliance on mean values, we analyzed the full distribution of individual network burst durations (Figure 4D). Our rationale was that not all neurons undergo the GABA shift simultaneously, and this cellular heterogeneity can influence network-level readouts in ways that average-based comparisons may obscure. Consistent with our previous findings (Mossink et al., 2022), network burst duration decreased over development of the control networks (Figure 4D), a characteristic parameter for functional inhibition by GABAergic neurons (Mossink et al., 2022; Wang et al., 2023). At DIV35 and DIV42, we observed a clear shift in the distribution toward shorter network burst duration with p.(V1393M) GABAergic neurons (Figure 4D), an effect that disappeared by DIV49. This suggests that p.(V1393M) GABAergic neurons exert an increased inhibitory drive during early development. To further confirm this, we treated the networks with picrotoxin (PTX) at DIV35 to assess how the network responded to acute removal of inhibitory control (Figure 4C) (Mossink et al., 2022). As expected, PTX treatment did not alter the mean firing rate in control networks at DIV35 (Figure 4E), a developmental stage at which the hyperpolarizing shift of the GABA reversal potential has not yet occurred in all neurons of the network (Mossink et al., 2022). However, in both p.(V1393M) networks, the mean firing rate increased upon treatment with PTX (Figure 4E). To assess responses to PTX more precisely, we quantified the proportion of prolonged network bursts exceeding 0.625 s, a threshold above which fewer than 2.5% of events occurred under basal conditions across all groups (Figure 4F). Following PTX treatment, a higher proportion of long-duration network bursts was observed in p.(V1393M) networks compared to their isogenic controls. These findings support the conclusion that the p.(V1393M) variant enhances GABAergic output during early network development.

Taken together, these results suggest that the *CACNA1A* p.(V1393M) variant induces a shift in E/I coordination, characterized by increased inhibition during early neuronal network development. It is worth highlighting that the patient carrying the p.(V1393M) missense variant exhibits the earliest and most pronounced symptoms among the individuals we investigated. This indicates that MEA-based neuronal networks provide a powerful platform for establishing genotype-phenotype correlations and assessing disease-related network dysfunction.

VUS in *CACNA1A* alter neuronal network activity but cannot be linked to a clinical phenotype when mimicked by CRISPR-Cas9

To uncover whether we could use changes in network developmental trajectories for different variant types and clinical phenotypes to classify VUS, we mimicked three variants in the Ctr iPSC line by genome editing. We mimicked a p.(N390K) missense variant from a patient presenting ataxia and migraine, a p.(R1434Q) missense variant from a patient with ataxia, epilepsy, and intellectual disability (ID), and a p.(R1857*) nonsense variant in mutually exclusive exon 37A from a patient who presented with ataxia and epilepsy (Figure 5A). In literature, three other patients have been described with the latter variant, presenting isolated episodic ataxia (Figure 5A) (Graves et al., 2008; Sintas et al., 2017). We used CRISPR-Cas9 to introduce the variants into Ctr, resulting in an exact mimic of p.(N390K) and p.(R1434Q) missense variants. These missense variants are classified as likely pathogenic by AlphaMissense (Figures 5B; Table 1) (Cheng et al., 2023). The p.(R1857*) nonsense variant was mimicked through deletion of five base pairs in exon 37A, predicting a p.(P1862Rfs*9) frameshift variant. To revert to a diagnostic framework with a simplified model, we differentiated these iPSCs into glutamatergic-only neuronal networks. We recorded their neuronal network activity over development and

Figure 4. Cav2.1 gain-of-function leads to an early increased inhibitory drive in iPSC-derived GABAergic neurons

(A) Schematic representation of the differentiation protocol from iPSCs to glutamatergic/GABAergic co-cultures.

(B) Schematic overview of co-cultured conditions used in this study.

(C) Representative rasterplots (60 s) of network activity of the indicated neuronal networks at days *in vitro* (DIV)35 before (basal) and after 5 min treatment with 100 μM picrotoxin (PTX).

(D) Density plots of network burst durations of Ctr glutamatergic neurons cultured with Ctr, Ctr_V1393M, Rescue_V1393M, or Patient_V1393M GABAergic neurons at DIV35, 42, and 49. $n = 12/2$ for Ctr, $n = 12/2$ for Ctr_V1393M, $n = 12/2$ for Rescue_V1393M, and $n = 12/2$ for Pat_V1393M. $^{***}p < 0.001$, Wilcoxon rank-sum test with continuity correction.

(E) Quantification of mean firing rate at DIV35 at baseline and after 5 min treatment with 100 μM PTX. $n = 4/2$ for Ctr, $n = 4/2$ for Ctr_V1393M, $n = 4/2$ for Rescue_V1393M, and $n = 4/2$ for Pat_V1393M. $^{*}p < 0.05$, $^{**}p < 0.01$, paired t test.

(F) Density plots of network burst durations of Ctr glutamatergic neurons cultured with Ctr, Ctr_V1393M, Rescue_V1393M, or Patient_V1393M GABAergic neurons at baseline and after 5 min treatment with 100 μM PTX at DIV35. $n = 4/2$ for Ctr, $n = 4/2$ for Ctr_V1393M, $n = 4/2$ for Rescue_V1393M, and $n = 4/2$ for Pat_V1393M. The percentage indicates the percentage of events after treatment with PTX exceeding 0.625 s, the threshold above which fewer than 2.5% of network burst durations occurred under basal conditions across all groups.

See also Figure S2.

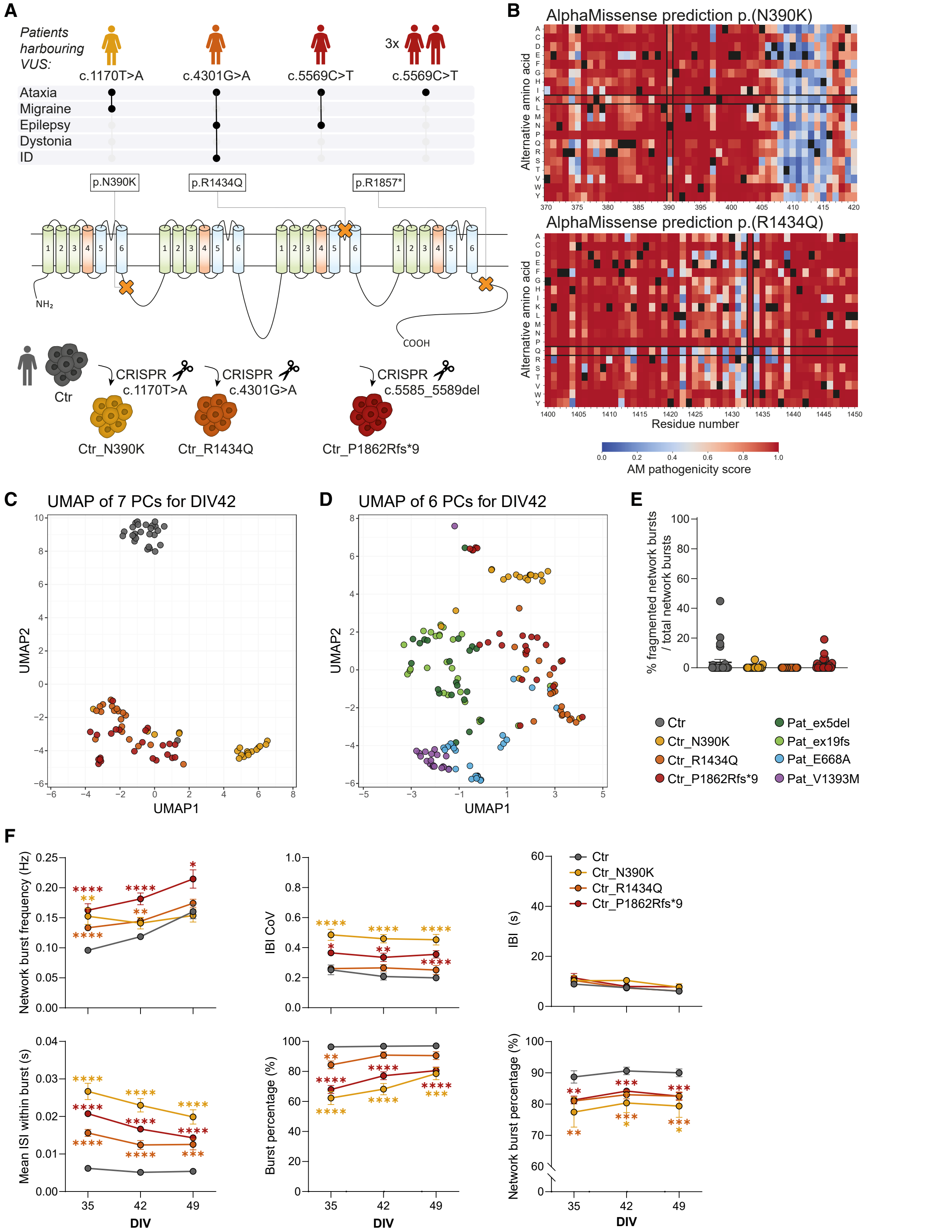

(legend on next page)

investigated how neuronal network activity compared to the Ctr and patient-derived networks in UMAP space (Figures 5C and 5D). Ctr and Ctr-derived mutant networks clustered distinctly, with each Ctr-derived mutant network forming a separate cluster. This suggests that introduction of these variants altered the control network function, all in a variant-specific manner. Ctr_R1434Q and Ctr_P1862Rfs*9 networks clustered closer to each other, suggesting some similarity in neurophysiological changes. These clusters did not overlap with the previously identified clusters (Figure 5D), preventing to link these variants to patient-derived networks based on their full network functional profile. Because epilepsy was reported in two of the patients harbouring VUS [p.(R1434Q) and p.(R1857*)], we first asked whether any of the recorded networks showed an increase in the percentage of fragmented network bursts. None of the networks demonstrated a change in the percentage of fragmented network bursts (Figure 5E), suggesting that these variants cannot fully be linked to seizurogenic activity. The same *CACNA1A* variant can lead to diverse clinical phenotypes depending on the genetic background (Lipman et al., 2022), as evidenced by p.(R1857*) manifesting as either isolated ataxia or a combination of ataxia and epilepsy. Therefore, introduction of variants p.(R1434Q) and p.(R1857*) in Ctr may not be sufficient to cause seizurogenic activity.

We then investigated whether these networks resembled any developmental trajectories as characterized for the patient-derived neuronal networks. Notably, network burst frequency was generally higher in Ctr than in the rescue networks (Figures 2C and 5F). In addition, all introduced variants led to higher network burst frequencies compared to Ctr at DIV35. In terms of developmental trajectories, Ctr_R1434Q and Ctr_P1862Rfs*9 resembled Ctr and Rescue_V1393M, whereas Ctr_N390K resembled the developmental trajectory of Pat_E668A (Figures 2C and 5F). Interestingly, these variants clinically both lead to ataxia combined with migraine, indicating that this developmental trajectory might be coupled to migraine as a clinical phenotype. Both Ctr_N390K and Ctr_P1862Rfs*9 showed a consistent elevated IBI CoV compared to Ctr over development, as well as a decreased network burst percentage (Figure 5F). The IBI remained unaltered, and we observed a higher mean ISI within burst and a lower burst percentage, which could be indicative of a loss-of-function effect of the variants.

Together, these results show functional changes in neuronal networks carrying *CACNA1A* VUS, supporting classification as likely pathogenic, as indicated for the missense variants by AlphaMissense. However, careful interpretation is required. By introducing these variants into a control line, it did not allow us to directly link functional changes to specific clinical phenotypes, and neurophysiological changes may vary depending on the genetic background.

DISCUSSION

MEA-based neuronal networks have been widely used to investigate genotype-phenotype correlations across various neurological disorders, including autism spectrum disorder, epilepsy, and Fragile X syndrome (Nageshappa et al., 2016; Deneault et al., 2019; Flaherty et al., 2019; Frega et al., 2019; Klein Gunnewiek et al., 2020; Tidball et al., 2020; Utami et al., 2020; Negraes et al., 2021; Que

Figure 5. Clustering of glutamatergic neuronal networks carrying introduced *CACNA1A* variants of uncertain significance based on their network activity over late development

(A) Schematic overview of the clinical phenotype of *CACNA1A* patients resembled in this study with their genetic variants (NM_00127221). These variants were mimicked in a control induced pluripotent stem cell (iPSC) line with CRISPR-Cas9 to generate isogenic lines. Genetic variants are also represented as orange crosses in a schematic representation of Cav2.1. Transmembrane domains are shown in green, voltage-sensitive domains in red, and pore-forming domains in blue.

(B) Pathogenicity scores predicted by AlphaMissense for missense variants.

(C and D) Uniform manifold approximation and projection (UMAP) analysis on seven principal components (PCs) obtained from network activity parameters at days *in vitro* (DIV)42 for all iPSC-derived networks carrying variants of uncertain significance (VUS) compared to the isogenic control (C) and compared to all patient-derived networks indicated in Figure 1A (D). n = 21/3 for Pat_ex5del, n = 25/4 for Pat_ex19fs, n = 27/4 for Pat_E668A, n = 19/3 for Pat_V1393M, n = 27/5 for Ctr, n = 20/4 for Ctr_N390K, n = 19/3 for Ctr_R1434Q, and n = 23/5 for Ctr_P1862Rfs*9.

(E) Quantification of the percentage fragmented over total network bursts at DIV49. n = 27/5 for Ctr, n = 20/4 for Ctr_N390K, n = 17/3 for Ctr_R1434Q, and n = 23/5 for Ctr_P1862Rfs*9. Data are represented as mean ± standard error of the mean (SEM). Kruskal-Wallis test with Dunn's test for multiple comparisons.

(F) Network activity parameters over DIV35, 42, and 49 for the different isogenic lines, including network burst frequency, inter-burst interval (IBI) coefficient of variation (CoV), IBI, mean inter-spike interval (ISI) within burst, burst percentage, and network burst percentage. n = 27/5 for Ctr, n = 20/4 for Ctr_N390K, n = *19/3* for Ctr_R1434Q, and n = *23/5* for Ctr_P1862Rfs*9. Data are represented as mean ± SEM. *$p < 0.05$, **$p < 0.01$, ***$p < 0.001$, ****$p < 0.0001$, two-way ANOVA with mixed-effects model if there are missing values, followed by Šídák's test for multiple comparisons.

Table 1. AlphaMissense predictions of *CACNA1A* missense variants of uncertain significance

Genetic variant	Coding variant	Protein variant	Pathogenicity score	Class	pLDDT score of original amino acid
Chr19: g.13334406A>T	c.1170T>A	p.(N390K)	0.975	likely pathogenic	70.54
Chr19: g.13259654C>T	c.4301G>A	p.(R1434Q)	0.997	likely pathogenic	88.45

Genetic variants are listed in GRCh38; coding variants are listed in NM_001127221.1.
pLDDT, predicted local distance difference test.

et al., 2021; Simkin et al., 2021; Trujillo et al., 2021; Linda et al., 2022; Mossink et al., 2022; Wang et al., 2022; van Hugte et al., 2023). These studies, using patient iPSC-derived and CRISPR-engineered neuronal networks, have demonstrated how disease-associated genetic variants can lead to alterations in network excitability, burst dynamics, and synchronization. In this study, we extend these approaches by performing a comprehensive characterization of the full network neurophysiological profile in *CACNA1A*-related disorders, integrating multiple electrophysiological parameters to derive network-level fingerprints. This systematic analysis allowed us to relate specific variant types and clinical phenotypes to distinct patterns of network activity, thereby contributing a more quantitative framework for interpreting functional consequences of *CACNA1A* variants. We showed that *null* alleles induced subtle changes in glutamatergic network activity, while missense variants exerted a more pronounced effect on network function. While network fingerprints remained relatively similar in case of dystonia co-occurring with ataxia, the network fingerprint appeared to be significantly different when clinical features such as migraine or epilepsy were present. We furthermore showed functional changes in neuronal networks when VUS were introduced in *CACNA1A*, supporting their classification as likely pathogenic. However, we could not relate these functional changes to specific clinical phenotypes as the network functional profile did not overlap with those of patient-derived networks and because the variants were introduced in a control line. CRISPR-Cas9 enables the introduction or correction of variants in iPSCs with an identical genetic background, providing a powerful approach to isolate the specific effects of a given variant. However, a variant's effect on cellular behaviour may be influenced by the broader genetic context, meaning it might not consistently manifest a disease-relevant effect across different backgrounds. This challenge is particularly evident when control iPSC lines are used as donors for introducing genetic variants, as they may lack the patient's unique genomic composition, potentially missing gene-gene interactions that contribute to disease pathogenesis. This limitation is particularly pronounced in the study of voltage-gated ion channels, where genetic modifier alleles can influence phenotypic variability through compensatory mechanisms. For instance, the broad phenotypic spectrum observed in *CACNA1A*-related disorders suggests the involvement of genetic modifiers. A study identified *UBR4* and *SLC1A3* as potential genetic modifiers that may exert a synergistic effect on *CACNA1A* variants, influencing disease severity and presentation (Choi et al., 2017). Furthermore, certain variants in *CACNA1A* were associated with earlier onset of seizures in individuals with Dravet syndrome, suggesting a modifying effect of these *CACNA1A* variants on the epileptic phenotype (Ohmori et al., 2013). However, whether a similar correlation exists for symptom onset in *CACNA1A*-related disorders remains to be determined. To address these complexities, we generally recommend the use of patient-derived cells in combination with multiple control lines to establish a well-characterized reference framework. Ideally, cells should be sourced from families in which all members carry the same variant but exhibit either phenotypic variability or a consistent phenotype. This approach allows for definitive conclusions about genotype-phenotype correlations while minimizing the influence of genetic background. When using CRISPR-Cas9, we suggest examining the variant of interest across multiple control genetic backgrounds to account for potential background-specific effects. Additionally, introducing known benign variants into the same iPSC lines can serve as essential controls to distinguish variant-specific effects from those arising due to genetic background variability or the editing process itself.

We here report a clear network fingerprint related to gain-of-function of $Ca_V2.1$. We identified certain network parameters, such as increased IBI CoV and fragmented network bursts, that are specifically modulated by the p.(V1393M) variant. Notably, the observation that this gain-of-function variant leads to network burst fragmentation is particularly interesting, as fragmentation has been linked to asynchronous neurotransmitter release driven by increased presynaptic calcium availability (Doorn et al., 2024; Pradeepan et al., 2024). This asynchronous release can in turn prolong excitation and enhance short-term synaptic depression (Doorn et al., 2024). Indeed, other gain-of-function variants have also been associated with enhanced short-term synaptic depression (Tottene et al., 2009; Di Guilmi et al., 2014), suggesting that network burst fragmentation may be a common feature of $Ca_V2.1$ gain-of-function.

The p.(V1393M) variant is recurrently observed among individuals with *CACNA1A*-related disorders (Lipman et al., 2022). Although the specific clinical phenotypes associated with this variant varied slightly among patients, ataxia and epilepsy are core features (Hamdan et al., 2017; Travaglini et al., 2017; Jiang et al., 2019; Zhang et al., 2020; Le Roux et al., 2021; Lipman et al., 2022; Niu et al., 2022; Kessi et al., 2023). This suggests that ataxia and epilepsy are a primary manifestation of the variant, independent of the patient's genetic background. This enabled us to correlate specific network parameters to the consistent clinical features associated with this variant, even when the variant was introduced into a control iPSC line. The fact that we could not replicate a seizurogenic network fingerprint for one of the VUS, p.(R1857*), may be due to the fact that this variant does not lead to epilepsy in different genetic backgrounds. Three other patients have been reported in literature with this variant, all of them showing classic episodic ataxia type 2 (Graves et al., 2008; Sintas et al., 2017). Therefore, mimicking this variant in a control line may not lead to seizurogenic activity. To determine whether the variant is the underlying cause of epilepsy in our proband, it would be crucial to use patient-derived iPSCs and include a family member as donor to control for potential confounding genetic factors.

Another possibility is that the chosen model may not fully capture variant-related effects. The effects of the variant could manifest in other cell types than the iPSC-derived glutamatergic neurons being studied. Since the variant is located in exon 37A—a mutually exclusive exon that undergoes a developmental switch—its expression dynamics may influence neurophysiological outcomes. Early in development, exon 37B is predominantly expressed, while expression of exon 37A gradually increases postnatally (Vigues et al., 2002; Chaudhuri, et al. 2004, 2005). If exon 37A is not sufficiently expressed in the current model, the expected fingerprint may not be detectable. In the adult brain, both isoforms are generally expressed at similar levels across most regions (Bourinet et al., 1999; Soong et al., 2002; Vigues et al., 2002; Chaudhuri et al., 2005); however, at the cellular level, distinct expression patterns emerge. Parvalbumin-positive interneurons in particular seem to rely exclusively on exon 37A (Huntley et al., 2020), suggesting that a more comprehensive model—including both glutamatergic and GABAergic neurons—may be necessary to fully capture the variant's effect.

We showed that a *CACNA1A* gain-of-function variant exerted effects on GABAergic neuronal functioning specifically, by co-culturing control glutamatergic neurons with p.(V1393M) GABAergic neurons. We demonstrated that p.(V1393M) GABAergic neurons showed an increased inhibitory drive in early development of the network. At this moment of neuronal network development, control networks may not have undergone the GABA shift (Mossink et al., 2022)—an increase in the KCC2:NKCC1 chloride cotransporter expression through which the chloride reversal potential hyperpolarizes (Ben-Ari et al., 2007). It remains to be determined whether the observed increase in inhibitory drive represents an early GABA shift or results from enhanced GABA release at the synapse. Interestingly, loss of $Ca_V2.1$ function compromised GABA release from parvalbumin-positive interneurons specifically, whereas signaling was preserved from somatostatin-positive interneurons (Rossignol et al., 2013). Because the p.(V1393M) variant has been shown to lead to gain-of-function, the increased inhibitory drive may also stem from increased GABA release. Although we have not characterized the specific interneuron subtypes within our glutamatergic/GABAergic networks, we demonstrate an increased inhibitory drive that is independent of genetic background. Therefore, we propose that these networks serve as a valuable platform for exploring genotype-phenotype correlations and their impact on neurophysiological activity in *CACNA1A*-related disorders.

Our findings further underscore the potential of specific network metrics, such as mean ISI within burst, burst percentage, and fragmented network bursts, as biomarkers for guiding variant-specific therapeutic strategies. For example, networks carrying variants like p.(V1393M), which exhibit fragmented bursting, may benefit from treatments targeting presynaptic calcium handling. In contrast, *null* alleles, with more subtle network effects in glutamatergic networks, may require modelling in glutamatergic/GABAergic co-cultures and treatments targeting the inhibitory circuit. These observations highlight the need for stratified treatment strategies based on the specific functional fingerprint of each variant.

We also demonstrate that genetic background can modulate these fingerprints, which may complicate the interpretation and translational relevance of functional findings. While some variant effects (e.g., p.(V1393M)-induced inhibitory drive) appear to be independent of genetic background, others, particularly VUS introduced into control lines, did not recapitulate expected phenotypes. This discrepancy may reflect the absence of disease-relevant modifier alleles in unrelated control lines. These observations raise the question whether functional studies should rely more heavily on patient-derived iPSC models, particularly for variants where the clinical presentation is variable or unclear. We propose that, where resources allow, validating findings in patient-derived cells provides a more reliable framework for understanding disease mechanisms. This approach may be especially valuable in the context of precision medicine, where both variant-specific and background-sensitive phenotypes could inform tailored therapeutic strategies.

Limitations of the study

Although we used multiple patient-derived neuronal lines, including some with CRISPR-Cas9-corrected/mimicking variants, the statistical power of this study is not sufficient to establish definitive genotype-phenotype correlations. Instead, our findings should be viewed as a proof of concept, highlighting both the strengths and limitations of iPSC-based neuronal modelling. One key limitation is the challenge of interpreting CRISPR-engineered VUS. While CRISPR-Cas9 remains a standard tool in the field, these engineered lines lack direct clinical data, making it difficult to contextualize their functional impact. Unlike patient-derived lines, CRISPR-edited lines do not inherently reflect the complex genetic and epigenetic background of an affected individual, which may influence neuronal network activity. This underscores the need for complementary approaches, such as functional assays in patient-derived cells and longitudinal clinical studies, to improve variant classification and our understanding of *CACNA1A*-related disorders.

METHODS

Human iPSC lines

In this study, we used a total of eleven iPSC lines, derived from five distinct individuals (supplemental methods, Figure S3). Informed consent was obtained from participants to collect and process their data. The institutional ethical review committee CMO Radboudumc, Nijmegen, the Netherlands, has given approval to conduct this research (CMO Radboudumc dossier numbers: 2017–3827, 2019–5250).

Generation of rtTA-*Ngn2*- or rtTA-*Ascl1*/*Dlx2*-positive iPSCs and neuronal differentiation

To differentiate iPSCs into glutamatergic neurons, rtTA-*Ngn2*-positive iPSCs were generated according to a previously published protocol (supplemental methods) (Zhang et al., 2013; Frega et al., 2017). In order to differentiate iPSCs into GABAergic neurons, rtTA-*Ascl1*/*Dlx2*-positive iPSCs were generated according to a recently published protocol (supplemental methods) (Yang et al., 2017; van Voorst et al., 2025). At 80%–90% confluency (DIV0), single cells were generated from rtTA-*Ngn2*-positive or rtTA-*Ascl1*-*Dlx2*-positive iPSCs for neuronal differentiation (supplemental methods). The cells were plated at a final density of approximately 600 cells/mm^2. For glutamatergic and GABAergic cocultures, cells were plated in a 50:50 ratio.

Micro-electrode array recordings and analysis

Spontaneous neuronal network activity was recorded from neuronal networks grown on 48-well Cytoview MEAs (Axion BioSystems, Atlanta, GA, USA) for 5 min in a Maestro Pro MEA system (Axion BioSystems) equipped with AxIS Navigator software (Axion BioSystems) when temperature and CO_2 were stable at 37°C and 5%, respectively. Within each well, 16 electrodes are embedded at the bottom in a 4 × 4 grid (diameter 50 μm, spacing 350 μm). The sampling frequency was set at 12.5 kHz. For spike detection, an adaptive threshold of ±6 standard deviations of the noise was applied, creating .spk files. Time-stamps per electrode were obtained from these raw .spk files using AxionFileLoader toolbox in MATLAB. Electrode and network bursts were detected using modified functions from the meaRtools R package (Gelfman et al., 2018). For electrode burst detection, we used the maximum ISI algorithm, setting maximum ISI to 0.1 s. Electrode bursts were restricted to be constituted by 5 spikes at least and to be longer than 0.05 s. Consecutive electrode bursts separated by less than 0.05 s were merged. For network bursts detection, time-stamps were binned in 2 ms intervals, and a Gaussian filter with a 70 ms window size was applied to the binned data. The smoothed signals from individual electrodes were standardized, combined, and then smoothed again using the same Gaussian filter. Network burst intervals were identified by thresholding the combined signal with the Otsu global thresholding algorithm, requiring the involvement of at least 25% of the electrodes and a duration of more than 0.1 s. In addition, we imposed a restriction on network bursts, requiring a minimum firing rate of 12 Hz in at least 25% of the actively participating electrodes. We identified a population of particularly short network bursts (<0.32 s) that followed "main network bursts" (>0.32 s) with no more than a 1.1-s gap (Figure 3B). These were termed "fragmented network bursts" and were analyzed separately. Various average and variability-related variables were extracted from electrode bursts, network bursts, and fragmented network bursts. These variables are detailed in Table S3.

To evaluate whether the different *CACNA1A* variants induced effects relative to the control network activity, we performed PCA on scaled parameters for DIV35, DIV42, and DIV49 using the prcomp function from the stats R package. We then applied UMAP to the PCs that accounted for more than 2.5% of the variance in the data. The number of PCs considered is indicated in the corresponding figures.

Single-cell electrophysiology

Single-cell recordings in whole-cell patch-clamp configuration were performed at DIV42 as previously described (Hommersom et al., 2024) and further described in the supplemental methods. We performed PCA on scaled parameters using the prcomp function from the stats R

package. We then applied UMAP to the PCs that accounted for more than 5% of the variance in the data.

Compounds

All compounds were prepared into concentrated stocks in dimethyl sulfoxide (DMSO) and stored at −20°C, unless mentioned otherwise. Glutamatergic-only cultures were treated over development with Cav2 inhibitors, PD-173212 (Sigma-Aldrich, #SML3506) and CNV2197944 (CNV’944; provided by the CACNA1A Foundation; stored at 4°C). Upon treatment, these compounds were first diluted 1:20 in Dulbecco’s phosphate-buffered saline with calcium and magnesium (Gibco, #14040117). Treatment started at DIV22 by diluting the compound 1:100 into the full volume of medium of the well. Every time the medium was refreshed afterward, the compound was diluted 1:100 into half of the volume of the well, resulting in maximum final concentrations of 500 nM for PD-173212 and 30 μM for CNV’944. We added DMSO as a vehicle in the same way, resulting in a maximum final DMSO percentage of 0.05%. We assessed network activity at DIV49.

At DIV34, we collected 50% pre-conditioned medium from glutamatergic/GABAergic co-cultures, while refreshing 50% of the medium. At DIV35, we assessed the basal network activity of these co-cultures for 5 min. After this recording, co-cultures were treated with 100 μM PTX (in 96% ethanol; Tocris, #1128) or 0.1% ethanol. We immediately assessed spontaneous network activity without GABAergic signaling for 20 min. Consecutively, PTX and vehicle were washed out by three gentle washes with plain Neurobasal medium (Gibco, #21103049), and medium was fully refreshed by combining the pre-conditioned medium in a 1:1 ratio with freshly prepared Neurobasal medium with supplements (supplemental methods).

Statistics

Statistical analysis was performed using GraphPad PRISM 10.1.2 (GraphPad Software, Inc., CA, USA). Each figure legend indicates how data are visualized and what tests were performed. In all figures, p values are indicated as follows: < 0.05 (*), < 0.01 (**), < 0.001 (***), < 0.0001 (****). In each figure legend, $n = (a)/(b)$ indicates the number of MEA wells or patched cells (a) per independent differentiation (b). We provide a summary of every statistical test performed per figure panel in Table S4.

RESOURCE AVAILABILITY

Lead contact

Requests for further information and resources should be directed to and will be fulfilled by the lead contact, Hans van Bokhoven (hansvanbokhoven@radboudumc.nl).

Materials availability

This study did not generate new unique reagents apart from iPSC lines.

Data and code availability

All data reported in this paper will be shared by the lead contact upon request. All original code has been deposited at GitHub and is publicly available at https://github.com/nadifkasri-lab/hommersom_2025_cacna1a_variants as of the date of publication. Any additional information required to reanalyze the data reported in this paper is available from the lead contact upon request.

ACKNOWLEDGMENTS

This research was made possible through the CACNA1A Foundation, with samples available from the COMBINEDBrain Biorepository. We wish to thank the Radboudumc Stem Cell Technology Center (https://www.radboudumc.nl/en/research/radboud-technology-centers/stem-cells) for genome editing and characterizing the Rescue_V1393M iPSC line. This work was supported by a grant from the Radboud University Medical Center and Donders Institute for Brain, Cognition, and Behaviour (B.P.C.v.d.W. and H.v.B.) and a grant from the CACNA1A Foundation (B.P.C.v.d.W. and M.P.H.). N.N.K. was supported by the BRAINMODEL ZonMw PSIDER program 10250022110003 and SFARI grant 890042.

AUTHOR CONTRIBUTIONS

B.P.C.v.d.W., N.N.K., and H.v.B. conceptualized and supervised the study. M.P.H. wrote the original draft of this manuscript and was responsible for the visualization of the data. B.P.C.v.d.W., N.N.K., and H.v.B. reviewed and edited the manuscript. M.P.H. and E.C. performed CRISPR-Cas9 experiments to generate iPSC models. L.D. performed experiments for the validation of iPSC lines. M.B. and E.v.B. performed MEA experiments together with M.P.H. S.P. created the MATLAB and R codes for analysis of the MEA data and provided support when these were executed by M.P.H. M.P.H. performed single-cell patch-clamp experiments and performed subsequent analyses.

DECLARATION OF INTERESTS

The authors declare no competing interests.

DECLARATION OF GENERATIVE AI AND AI-ASSISTED TECHNOLOGIES IN THE WRITING PROCESS

During the preparation of this work, the authors used ChatGPT to improve language and readability. After using this tool/service, the authors reviewed and edited the content as needed and take full responsibility for the content of the publication.

SUPPLEMENTAL INFORMATION

Supplemental information can be found online at https://doi.org/10.1016/j.stemcr.2025.102783.

Received: June 17, 2025
Revised: December 17, 2025
Accepted: December 18, 2025
Published: January 22, 2026

REFERENCES

Bahamonde, M.I., Serra, S.A., Drechsel, O., Rahman, R., Marcé-Grau, A., Prieto, M., Ossowski, S., Macaya, A., and Fernández-Fernández, J.M. (2015). A Single Amino Acid Deletion (ΔF1502) in the S6 Segment of CaV2.1 Domain III Associated with Congenital Ataxia Increases Channel Activity and Promotes Ca2+ Influx. PLoS One *10*, e0146035. https://doi.org/10.1371/journal.pone.0146035.

Ben-Ari, Y., Gaiarsa, J.L., Tyzio, R., and Khazipov, R. (2007). GABA: a pioneer transmitter that excites immature neurons and generates primitive oscillations. Physiol. Rev. *87*, 1215–1284. https://doi.org/10.1152/physrev.00017.2006.

Bourinet, E., Soong, T.W., Sutton, K., Slaymaker, S., Mathews, E., Monteil, A., Zamponi, G.W., Nargeot, J., and Snutch, T.P. (1999). Splicing of α1A subunit gene generates phenotypic variants of P- and Q-type calcium channels. Nat. Neurosci. *2*, 407–415. https://doi.org/10.1038/8070.

Buraei, Z., and Yang, J. (2010). The ß subunit of voltage-gated Ca2+ channels. Physiol. Rev. *90*, 1461–1506. https://doi.org/10.1152/physrev.00057.2009.

Chaudhuri, D., Alseikhan, B.A., Chang, S.Y., Soong, T.W., and Yue, D.T. (2005). Developmental Activation of Calmodulin-Dependent Facilitation of Cerebellar P-Type Ca^{2+} Current. J. Neurosci. *25*, 8282–8294. https://doi.org/10.1523/jneurosci.2253-05.2005.

Chaudhuri, D., Chang, S.Y., DeMaria, C.D., Alvania, R.S., Soong, T.W., and Yue, D.T. (2004). Alternative splicing as a molecular switch for Ca2+/calmodulin-dependent facilitation of P/Q-type Ca2+ channels. J. Neurosci. *24*, 6334–6342. https://doi.org/10.1523/jneurosci.1712-04.2004.

Cheng, J., Novati, G., Pan, J., Bycroft, C., Žemgulytė, A., Applebaum, T., Pritzel, A., Wong, L.H., Zielinski, M., Sargeant, T., et al. (2023). Accurate proteome-wide missense variant effect prediction with AlphaMissense. Science *381*, eadg7492. https://doi.org/10.1126/science.adg7492.

Choi, K.-D., Kim, J.-S., Kim, H.-J., Jung, I., Jeong, S.-H., Lee, S.-H., Kim, D.U., Kim, S.-H., Choi, S.Y., Shin, J.-H., et al. (2017). Genetic Variants Associated with Episodic Ataxia in Korea. Sci. Rep. *7*, 13855. https://doi.org/10.1038/s41598-017-14254-7.

Condliffe, S.B., Fratangeli, A., Munasinghe, N.R., Saba, E., Passafaro, M., Montrasio, C., Ferrari, M., Rosa, P., and Carrera, P. (2013). The E1015K Variant in the Synprint Region of the CaV2.1 Channel Alters Channel Function and Is Associated with Different Migraine Phenotypes. J. Biol. Chem. *288*, 33873–33883. https://doi.org/10.1074/jbc.M113.497701.

Cuenca-León, E., Banchs, I., Serra, S.A., Latorre, P., Fernàndez-Castillo, N., Corominas, R., Valverde, M.A., Volpini, V., Fernández-Fernández, J.M., Macaya, A., and Cormand, B. (2009). Late-onset episodic ataxia type 2 associated with a novel loss-of-function mutation in the CACNA1A gene. J. Neurol. Sci. *280*, 10–14. https://doi.org/10.1016/j.jns.2009.01.005.

Davies, A., Hendrich, J., Van Minh, A.T., Wratten, J., Douglas, L., and Dolphin, A.C. (2007). Functional biology of the α2δ subunits of voltage-gated calcium channels. Trends Pharmacol. Sci. *28*, 220–228. https://doi.org/10.1016/j.tips.2007.03.005.

Deneault, E., Faheem, M., White, S.H., Rodrigues, D.C., Sun, S., Wei, W., Piekna, A., Thompson, T., Howe, J.L., Chalil, L., et al. (2019). CNTN5-/+or EHMT2-/+human iPSC-derived neurons from individuals with autism develop hyperactive neuronal networks. eLife *8*, e40092. https://doi.org/10.7554/eLife.40092.

Di Guilmi, M.N., Wang, T., Inchauspe, C.G., Forsythe, I.D., Ferrari, M.D., van den Maagdenberg, A.M.J.M., Borst, J.G.G., and Uchitel, O.D. (2014). Synaptic gain-of-function effects of mutant Cav2.1 channels in a mouse model of familial hemiplegic migraine are due to increased basal [Ca2+]i. J. Neurosci. *34*, 7047–7058. https://doi.org/10.1523/jneurosci.2526-13.2014.

Doorn, N., Voogd, E.J.H.F., Levers, M.R., van Putten, M.J.A.M., and Frega, M. (2024). Breaking the burst: Unveiling mechanisms behind fragmented network bursts in patient-derived neurons. Stem Cell Rep. *19*, 1583–1597. https://doi.org/10.1016/j.stemcr.2024.09.001.

Flaherty, E., Zhu, S., Barretto, N., Cheng, E., Deans, P.J.M., Fernando, M.B., Schrode, N., Francoeur, N., Antoine, A., Alganem, K., et al. (2019). Neuronal impact of patient-specific aberrant NRXN1α splicing. Nat. Genet. *51*, 1679–1690. https://doi.org/10.1038/s41588-019-0539-z.

Frega, M., Linda, K., Keller, J.M., Gümüş-Akay, G., Mossink, B., van Rhijn, J.-R., Negwer, M., Klein Gunnewiek, T., Foreman, K., Kompier, N., et al. (2019). Neuronal network dysfunction in a model for Kleefstra syndrome mediated by enhanced NMDAR signaling. Nat. Commun. *10*, 4928. https://doi.org/10.1038/s41467-019-12947-3.

Frega, M., van Gestel, S.H.C., Linda, K., van der Raadt, J., Keller, J., Van Rhijn, J.R., Schubert, D., Albers, C.A., and Nadif Kasri, N. (2017). Rapid Neuronal Differentiation of Induced Pluripotent Stem Cells for Measuring Network Activity on Micro-electrode Arrays. J. Vis. Exp. *119*, 54900. https://doi.org/10.3791/54900.

Gandini, M.A., Souza, I.A., Ferron, L., Innes, A.M., and Zamponi, G.W. (2021). The de novo CACNA1A pathogenic variant Y1384C associated with hemiplegic migraine, early onset cerebellar atrophy and developmental delay leads to a loss of Cav2.1 channel function. Mol. Brain *14*, 27. https://doi.org/10.1186/s13041-021-00745-2.

Garza-López, E., González-Ramírez, R., Gandini, M.A., Sandoval, A., and Felix, R. (2013). The familial hemiplegic migraine type 1 mutation K1336E affects direct G protein-mediated regulation of neuronal P/Q-type Ca2+ channels. Cephalalgia *33*, 398–407. https://doi.org/10.1177/0333102412475236.

Garza-López, E., Sandoval, A., González-Ramírez, R., Gandini, M.A., Van den Maagdenberg, A., De Waard, M., and Felix, R. (2012). Familial hemiplegic migraine type 1 mutations W1684R and V1696I alter G protein-mediated regulation of CaV2.1 voltage-gated calcium channels. Biochim. Biophys. Acta Mol. Basis Dis. *1822*, 1238–1246. https://doi.org/10.1016/j.bbadis.2012.04.008.

Gelfman, S., Wang, Q., Lu, Y.-F., Hall, D., Bostick, C.D., Dhindsa, R., Halvorsen, M., McSweeney, K.M., Cotterill, E., Edinburgh, T., et al. (2018). meaRtools: An R package for the analysis of neuronal networks recorded on microelectrode arrays. PLoS Comput. Biol. *14*, e1006506. https://doi.org/10.1371/journal.pcbi.1006506.

Graves, T.D., Imbrici, P., Kors, E.E., Terwindt, G.M., Eunson, L.H., Frants, R.R., Haan, J., Ferrari, M.D., Goadsby, P.J., Hanna, M.G., et al. (2008). Premature stop codons in a facilitating EF-hand splice variant of CaV2.1 cause episodic ataxia type 2. Neurobiol. Dis. *32*, 10–15. https://doi.org/10.1016/j.nbd.2008.06.002.

Guida, S., Trettel, F., Pagnutti, S., Mantuano, E., Tottene, A., Veneziano, L., Fellin, T., Spadaro, M., Stauderman, K., Williams, M., et al. (2001). Complete Loss of P/Q Calcium Channel Activity Caused by a CACNA1A Missense Mutation Carried by Patients with Episodic Ataxia Type 2. Am. J. Hum. Genet. *68*, 759–764. https://doi.org/10.1086/318804.

Hamdan, F.F., Myers, C.T., Cossette, P., Lemay, P., Spiegelman, D., Laporte, A.D., Nassif, C., Diallo, O., Monlong, J., Cadieux-Dion, M., et al. (2017). High Rate of Recurrent De Novo Mutations in Developmental and Epileptic Encephalopathies. Am. J. Hum. Genet. *101*, 664–685. https://doi.org/10.1016/j.ajhg.2017.09.008.

Hommersom, M.P., Doorn, N., Puvogel, S., Lewerissa, E.I., Mordelt, A., Ciptasari, U., Kampshoff, F., Dillen, L., van Beusekom, E., Oudakker, A., et al. (2025). CACNA1A haploinsufficiency leads to reduced synaptic function and increased intrinsic excitability. Brain *148*, 1286–1301. https://doi.org/10.1093/brain/awae330.

Hommersom, M.P., van Prooije, T.H., Pennings, M., Schouten, M.I., van Bokhoven, H., Kamsteeg, E.J., and van de Warrenburg, B.P.C. (2022). The complexities of CACNA1A in clinical neurogenetics. J. Neurol. *269*, 3094–3108. https://doi.org/10.1007/s00415-021-10897-9.

Huang, Y.C., Pirri, J.K., Rayes, D., Gao, S., Mulcahy, B., Grant, J., Saheki, Y., Francis, M.M., Zhen, M., and Alkema, M.J. (2019). Gain-of-function mutations in the UNC-2/CaV2α channel lead to excitation-dominant synaptic transmission in Caenorhabditis elegans. eLife *8*, e45905. https://doi.org/10.7554/eLife.45905.

Huntley, M.A., Srinivasan, K., Friedman, B.A., Wang, T.-M., Yee, A.X., Wang, Y., Kaminker, J.S., Sheng, M., Hansen, D.V., and Hanson, J.E. (2020). Genome-Wide Analysis of Differential Gene Expression and Splicing in Excitatory Neurons and Interneuron Subtypes. J. Neurosci. *40*, 958–973. https://doi.org/10.1523/jneurosci.1615-19.2019.

Indelicato, E., and Boesch, S. (2021). From Genotype to Phenotype: Expanding the Clinical Spectrum of CACNA1A Variants in the Era of Next Generation Sequencing. Front. Neurol. *12*, 639994. https://doi.org/10.3389/fneur.2021.639994.

Jiang, X., Raju, P.K., D'Avanzo, N., Lachance, M., Pepin, J., Dubeau, F., Mitchell, W.G., Bello-Espinosa, L.E., Pierson, T.M., Minassian, B.A., et al. (2019). Both gain-of-function and loss-of-function de novo 1A mutations cause severe developmental epileptic encephalopathies in the spectrum of Lennox-Gastaut syndrome. Epilepsia *60*, 1881–1894. https://doi.org/10.1111/epi.16316.

Kessi, M., Chen, B., Pang, N., Yang, L., Peng, J., He, F., and Yin, F. (2023). The genotype–phenotype correlations of the CACNA1A-related neurodevelopmental disorders: a small case series and literature reviews. Original Research. Front. Mol. Neurosci. *16*, 1222321. https://doi.org/10.3389/fnmol.2023.1222321.

Klein Gunnewiek, T.M., Van Hugte, E.J.H., Frega, M., Guardia, G.S., Foreman, K., Panneman, D., Mossink, B., Linda, K., Keller, J.M., Schubert, D., et al. (2020). m. 3243A > G-Induced Mitochondrial Dysfunction Impairs Human Neuronal Development and Reduces Neuronal Network Activity and Synchronicity. Cell Rep. *31*, 107538. https://doi.org/10.1016/j.celrep.2020.107538.

Le Roux, M., Barth, M., Gueden, S., Desbordes de Cepoy, P., Aeby, A., Vilain, C., Hirsch, E., de Saint Martin, A., Portes, V., Lesca, G., et al. (2021). CACNA1A-associated epilepsy: Electroclinical findings and treatment response on seizures in 18 patients. Eur. J. Paediatr. Neurol. *33*, 75–85. https://doi.org/10.1016/j.ejpn.2021.05.010.

Linda, K., Lewerissa, E.I., Verboven, A.H.A., Gabriele, M., Frega, M., Klein Gunnewiek, T.M., Devilee, L., Ulferts, E., Hommersom, M., Oudakker, A., et al. (2022). Imbalanced autophagy causes synaptic deficits in a human model for neurodevelopmental disorders. Autophagy *18*, 423–442. https://doi.org/10.1080/15548627.2021.1936777.

Lipman, A.R., Fan, X., Shen, Y., and Chung, W.K. (2022). Clinical and genetic characterization of -related disease. Clin. Genet. *102*, 288–295. https://doi.org/10.1111/cge.14180.

Llinás, R., Sugimori, M., Lin, J.W., and Cherksey, B. (1989). Blocking and isolation of a calcium channel from neurons in mammals and cephalopods utilizing a toxin fraction (FTX) from funnel-web spider poison. Proc. Natl. Acad. Sci. USA *86*, 1689–1693. https://doi.org/10.1073/pnas.86.5.1689.

Lübbert, M., Goral, R.O., Keine, C., Thomas, C., Guerrero-Given, D., Putzke, T., Satterfield, R., Kamasawa, N., and Young, S.M., Jr. (2019). Ca(V)2.1 α(1) Subunit Expression Regulates Presynaptic Ca(V)2.1 Abundance and Synaptic Strength at a Central Synapse. Neuron *101*, 260–273.e6. https://doi.org/10.1016/j.neuron.2018.11.028.

Luebke, J.I., Dunlap, K., and Turner, T.J. (1993). Multiple calcium channel types control glutamatergic synaptic transmission in the hippocampus. Neuron *11*, 895–902. https://doi.org/10.1016/0896-6273(93)90119-c.

Lupien-Meilleur, A., Jiang, X., Lachance, M., Taschereau-Dumouchel, V., Gagnon, L., Vanasse, C., Lacaille, J.C., and Rossignol, E. (2021). Reversing frontal disinhibition rescues behavioural deficits in models of CACNA1A-associated neurodevelopment disorders. Mol. Psychiatry *26*, 7225–7246. https://doi.org/10.1038/s41380-021-01175-1.

Mossink, B., van Rhijn, J.-R., Wang, S., Linda, K., Vitale, M.R., Zöller, J.E.M., van Hugte, E.J.H., Bak, J., Verboven, A.H.A., Selten, M., et al. (2022). Cadherin-13 is a critical regulator of GABAergic modulation in human stem-cell-derived neuronal networks. Mol. Psychiatry *27*, 1–18. https://doi.org/10.1038/s41380-021-01117-x.

Mossink, B., Verboven, A.H.A., van Hugte, E.J.H., Klein Gunnewiek, T.M., Parodi, G., Linda, K., Schoenmaker, C., Kleefstra, T., Kozicz, T., van Bokhoven, H., et al. (2021). Human neuronal networks on micro-electrode arrays are a highly robust tool to study disease-specific genotype-phenotype correlations in vitro. Stem Cell Rep. *16*, 2182–2196. https://doi.org/10.1016/j.stemcr.2021.07.001.

Nageshappa, S., Carromeu, C., Trujillo, C.A., Mesci, P., Espuny-Camacho, I., Pasciuto, E., Vanderhaeghen, P., Verfaillie, C.M., Raitano, S., Kumar, A., et al. (2016). Altered neuronal network and rescue in a human MECP2 duplication model. Mol. Psychiatry *21*, 178–188. https://doi.org/10.1038/mp.2015.128.

Negraes, P.D., Trujillo, C.A., Yu, N.-K., Wu, W., Yao, H., Liang, N., Lautz, J.D., Kwok, E., McClatchy, D., Diedrich, J., et al. (2021). Altered network and rescue of human neurons derived from individuals with early-onset genetic epilepsy. Mol. Psychiatry *26*, 7047–7068. https://doi.org/10.1038/s41380-021-01104-2.

Niu, X., Yang, Y., Chen, Y., Cheng, M., Liu, M., Ding, C., Tian, X., Yang, Z., Jiang, Y., and Zhang, Y. (2022). Genotype–phenotype correlation of CACNA1A variants in children with epilepsy. Dev. Med. Child Neurol. *64*, 105–111. https://doi.org/10.1111/dmcn.14985.

Ohmori, I., Ouchida, M., Kobayashi, K., Jitsumori, Y., Mori, A., Michiue, H., Nishiki, T., Ohtsuka, Y., and Matsui, H. (2013). CACNA1A variants may modify the epileptic phenotype of Dravet syndrome. Neurobiol. Dis. *50*, 209–217. https://doi.org/10.1016/j.nbd.2012.10.016.

Okada, T., Kato, D., Nomura, Y., Obata, N., Quan, X., Morinaga, A., Yano, H., Guo, Z., Aoyama, Y., Tachibana, Y., et al. (2021). Pain induces stable, active microcircuits in the somatosensory cortex that provide a therapeutic target. Sci. Adv. *7*, eabd8261. https://doi.org/10.1126/sciadv.abd8261.

Pradeepan, K.S., McCready, F.P., Wei, W., Khaki, M., Zhang, W., Salter, M.W., Ellis, J., and Martinez-Trujillo, J. (2024). Calcium-Dependent Hyperexcitability in Human Stem Cell-Derived Rett Syndrome Neuronal Networks. Biol. Psychiatry Glob. Open Sci. *4*, 100290. https://doi.org/10.1016/j.bpsgos.2024.100290.

Que, Z., Olivero-Acosta, M.I., Zhang, J., Eaton, M., Tukker, A.M., Chen, X., Wu, J., Xie, J., Xiao, T., Wettschurack, K., et al. (2021). Hyperexcitability and Pharmacological Responsiveness of Cortical Neurons Derived from Human iPSCs Carrying Epilepsy-Associated Sodium Channel Nav1.2-L1342P Genetic Variant. J. Neurosci. *41*, 10194–10208. https://doi.org/10.1523/jneurosci.0564-21.2021.

Rossignol, E., Kruglikov, I., van den Maagdenberg, A.M.J.M., Rudy, B., and Fishell, G. (2013). CaV 2.1 ablation in cortical interneurons selectively impairs fast-spiking basket cells and causes generalized seizures. Ann. Neurol. *74*, 209–222. https://doi.org/10.1002/ana.23913.

Simkin, D., Marshall, K.A., Vanoye, C.G., Desai, R.R., Bustos, B.I., Piyevsky, B.N., Ortega, J.A., Forrest, M., Robertson, G.L., Penzes, P., et al. (2021). Dyshomeostatic modulation of Ca2+-activated K+ channels in a human neuronal model of KCNQ2 encephalopathy. eLife *10*, e64434. https://doi.org/10.7554/eLife.64434.

Singh, M., Sapkota, K., Sakimura, K., Kano, M., Cowell, R.M., Overstreet-Wadiche, L., Hablitz, J.J., and Nakazawa, K. (2023). Maturation of GABAergic Synaptic Transmission From Neocortical Parvalbumin Interneurons Involves N-methyl-D-aspartate Receptor Recruitment of Cav2.1 Channels. Neuroscience *513*, 38–53. https://doi.org/10.1016/j.neuroscience.2023.01.007.

Sintas, C., Carreño, O., Fernàndez-Castillo, N., Corominas, R., Vila-Pueyo, M., Toma, C., Cuenca-León, E., Barroeta, I., Roig, C., Volpini, V., et al. (2017). Mutation Spectrum in the CACNA1A Gene in 49 Patients with Episodic Ataxia. Sci. Rep. *7*, 2514. https://doi.org/10.1038/s41598-017-02554-x.

Soong, T.W., DeMaria, C.D., Alvania, R.S., Zweifel, L.S., Liang, M.C., Mittman, S., Agnew, W.S., and Yue, D.T. (2002). Systematic Identification of Splice Variants in Human P/Q-Type Channel $\alpha_1$2.1 Subunits: Implications for Current Density and Ca^{2+}-Dependent Inactivation. J. Neurosci. *22*, 10142–10152. https://doi.org/10.1523/jneurosci.22-23-10142.2002.

Takahashi, T., and Momiyama, A. (1993). Different types of calcium channels mediate central synaptic transmission. Nature *366*, 156–158. https://doi.org/10.1038/366156a0.

Thalhammer, A., Contestabile, A., Ermolyuk, Y.S., Ng, T., Volynski, K.E., Soong, T.W., Goda, Y., and Cingolani, L.A. (2017). Alternative Splicing of P/Q-Type Ca(2+) Channels Shapes Presynaptic Plasticity. Cell Rep. *20*, 333–343. https://doi.org/10.1016/j.celrep.2017.06.055.

Tidball, A.M., Lopez-Santiago, L.F., Yuan, Y., Glenn, T.W., Margolis, J.L., Clayton Walker, J., Kilbane, E.G., Miller, C.A., Martina Bebin, E., Scott Perry, M., et al. (2020). Variant-specific changes in persistent or resurgent sodium current in SCN8A-related epilepsy patient-derived neurons. Brain *143*, 3025–3040. https://doi.org/10.1093/brain/awaa247.

Tottene, A., Conti, R., Fabbro, A., Vecchia, D., Shapovalova, M., Santello, M., van den Maagdenberg, A.M.J.M., Ferrari, M.D., and Pietrobon, D. (2009). Enhanced Excitatory Transmission at Cortical Synapses as the Basis for Facilitated Spreading Depression in CaV2.1 Knockin Migraine Mice. Neuron *61*, 762–773. https://doi.org/10.1016/j.neuron.2009.01.027.

Travaglini, L., Nardella, M., Bellacchio, E., D'Amico, A., Capuano, A., Frusciante, R., Di Capua, M., Cusmai, R., Barresi, S., Morlino, S., et al. (2017). Missense mutations of CACNA1A are a frequent cause of autosomal dominant nonprogressive congenital ataxia. Eur. J. Paediatr. Neurol. *21*, 450–456. https://doi.org/10.1016/j.ejpn.2016.11.005.

Trujillo, C.A., Adams, J.W., Negraes, P.D., Carromeu, C., Tejwani, L., Acab, A., Tsuda, B., Thomas, C.A., Sodhi, N., Fichter, K.M., et al. (2021). Pharmacological reversal of synaptic and network pathology in human MECP2-KO neurons and cortical organoids. EMBO Mol. Med. *13*, e12523. https://doi.org/10.15252/emmm.202012523.

Turner, T.J., Adams, M.E., and Dunlap, K. (1992). Calcium channels coupled to glutamate release identified by omega-Aga-IVA. Science *258*, 310–313. https://doi.org/10.1126/science.1357749.

Uchitel, O.D., Protti, D.A., Sanchez, V., Cherksey, B.D., Sugimori, M., and Llinás, R. (1992). P-type voltage-dependent calcium channel mediates presynaptic calcium influx and transmitter release in mammalian synapses. Proc. Natl. Acad. Sci. USA *89*, 3330–3333. https://doi.org/10.1073/pnas.89.8.3330.

Utami, K.H., Skotte, N.H., Colaço, A.R., Yusof, N.A.B.M., Sim, B., Yeo, X.Y., Bae, H.-G., Garcia-Miralles, M., Radulescu, C.I., Chen, Q., et al. (2020). Integrative Analysis Identifies Key Molecular Signatures Underlying Neurodevelopmental Deficits in Fragile X Syndrome. Biol. Psychiatry *88*, 500–511. https://doi.org/10.1016/j.biopsych.2020.05.005.

van Hugte, E.J.H., Lewerissa, E.I., Wu, K.M., Scheefhals, N., Parodi, G., van Voorst, T.W., Puvogel, S., Kogo, N., Keller, J.M., Frega, M., et al. (2023). SCN1A-deficient excitatory neuronal networks display mutation-specific phenotypes. Brain *146*, 5153–5167. https://doi.org/10.1093/brain/awad245.

van Voorst, T.W., van Boven, M.A., Marinus, K.I., Colón-Mercado, J.M., Schretzmeir, J., Haag, C., Toonen, R.F., Koopmans, F., Ward,

M.E., Smit, A.B., et al. (2025). One-step induction of human GABAergic neurons promotes presynaptic development & synapse maturation. Preprint at bioRxiv. https://doi.org/10.1101/2025.06.30.662293.

Vigues, S., Gastaldi, M., Massacrier, A., Cau, P., and Valmier, J. (2002). The α1A subunits of rat brain calcium channels are developmentally regulated by alternative RNA splicing. Neuroscience *113*, 509–517. https://doi.org/10.1016/S0306-4522(02)00213-0.

Wang, S., Hesen, R., Mossink, B., Nadif Kasri, N., and Schubert, D. (2023). Generation of glutamatergic/GABAergic neuronal co-cultures derived from human induced pluripotent stem cells for characterizing E/I balance in vitro. STAR Protoc. *4*, 101967. https://doi.org/10.1016/j.xpro.2022.101967.

Wang, S., Rhijn, J.R.v., Akkouh, I., Kogo, N., Maas, N., Bleeck, A., Ortiz, I.S., Lewerissa, E., Wu, K.M., Schoenmaker, C., et al. (2022). Loss-of-function variants in the schizophrenia risk gene SETD1A alter neuronal network activity in human neurons through the cAMP/PKA pathway. Cell Rep. *39*, 110790. https://doi.org/10.1016/j.celrep.2022.110790.

Wheeler, D.B., Randall, A., and Tsien, R.W. (1994). Roles of N-type and Q-type Ca2+ channels in supporting hippocampal synaptic transmission. Science *264*, 107–111. https://doi.org/10.1126/science.7832825.

Yang, N., Chanda, S., Marro, S., Ng, Y.-H., Janas, J.A., Haag, D., Ang, C.E., Tang, Y., Flores, Q., Mall, M., et al. (2017). Generation of pure GABAergic neurons by transcription factor programming. Nat. Methods *14*, 621–628. https://doi.org/10.1038/nmeth.4291.

Yu, Y., Maureira, C., Liu, X., and McCormick, D. (2010). P/Q and N channels control baseline and spike-triggered calcium levels in neocortical axons and synaptic boutons. J. Neurosci. *30*, 11858–11869. https://doi.org/10.1523/jneurosci.2651-10.2010.

Zhang, L., Wen, Y., Zhang, Q., Chen, Y., Wang, J., Shi, K., Du, L., and Bao, X. (2020). CACNA1A Gene Variants in Eight Chinese Patients With a Wide Range of Phenotypes. Front. Pediatr. *8*, 577544. https://doi.org/10.3389/fped.2020.577544.

Zhang, Y., Pak, C., Han, Y., Ahlenius, H., Zhang, Z., Chanda, S., Marro, S., Patzke, C., Acuna, C., Covy, J., et al. (2013). Rapid single-step induction of functional neurons from human pluripotent stem cells. Neuron *78*, 785–798. https://doi.org/10.1016/j.neuron.2013.05.029.

Stem Cell Reports, Volume *21*

Supplemental Information

Human neuronal networks on micro-electrode arrays as a tool to assess genotype-phenotype correlation in *CACNA1A*-related disorders

Marina P. Hommersom, Sofía Puvogel, Nicky Scheefhals, Eleonora Carpentiero, Marga Bouma, Ellen van Beusekom, Lieke Dillen, Bart P.C. van de Warrenburg, Nael Nadif Kasri, and Hans van Bokhoven

Supplemental Information

Supplemental Figures

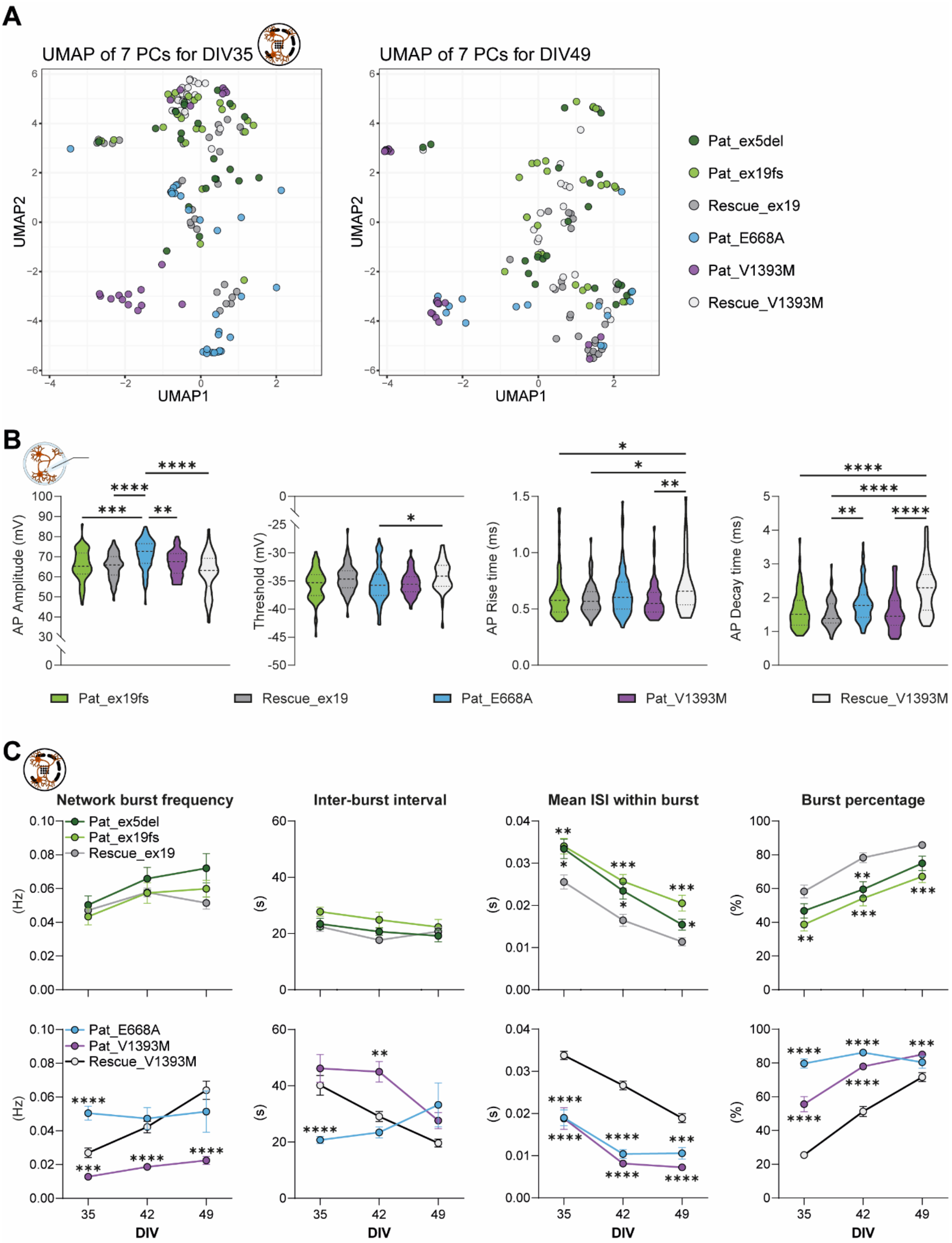

Figure S1 Quantification of single-cell and network electrophysiological parameters of patient-derived glutamatergic neurons, related to Figure 1 and 2. (**A**) Uniform manifold approximation and projection (UMAP) analysis on seven principal components (PCs) obtained from network activity parameters at days *in vitro* (DIV)35, and 49 for all iPSC-derived networks indicated in Figure 1A. (**B**) Quantification of single-cell electrophysiological properties including action potential (AP) amplitude, threshold, rise time and decay time at DIV42. *n = 68/8* for Pat_ex19fs, *n = 63/7* for Rescue_ex19, *n = 73/5* for Pat_E668A, *n = 57/4* for Pat_V1393M, and *n = 41/2* for Rescue_V1393M. Dashed line

represents the median, dotted line represents the quartiles. *$P < 0.05$, **$P < 0.01$ ***$P < 0.001$, ****$P < 0.0001$. Kruskal-Wallis test with Dunn's test for multiple comparisons. (**C**) Network activity parameters over days *in vitro* (DIV)35, 42, and 49 for the patient and isogenic rescue lines, including network burst frequency, inter-burst interval, mean inter spike interval (ISI) within burst, and burst percentage. *n = 21/3* for Pat_ex5del, *n = 25/4* for Pat_ex19fs, *n = 29/4* for Rescue_ex19, *n = 27/4* for Pat_E668A, *n = 19/3* for Pat_V1393M, and *n = 20/2* for Rescue_V1393M. Data represented as mean ± standard error of the mean (SEM). *$P < 0.05$, **$P < 0.01$, ***$P < 0.001$, ****$P < 0.0001$. Two-way ANOVA with mixed-effects model if there are missing values, followed by Šídák's test for multiple comparisons.

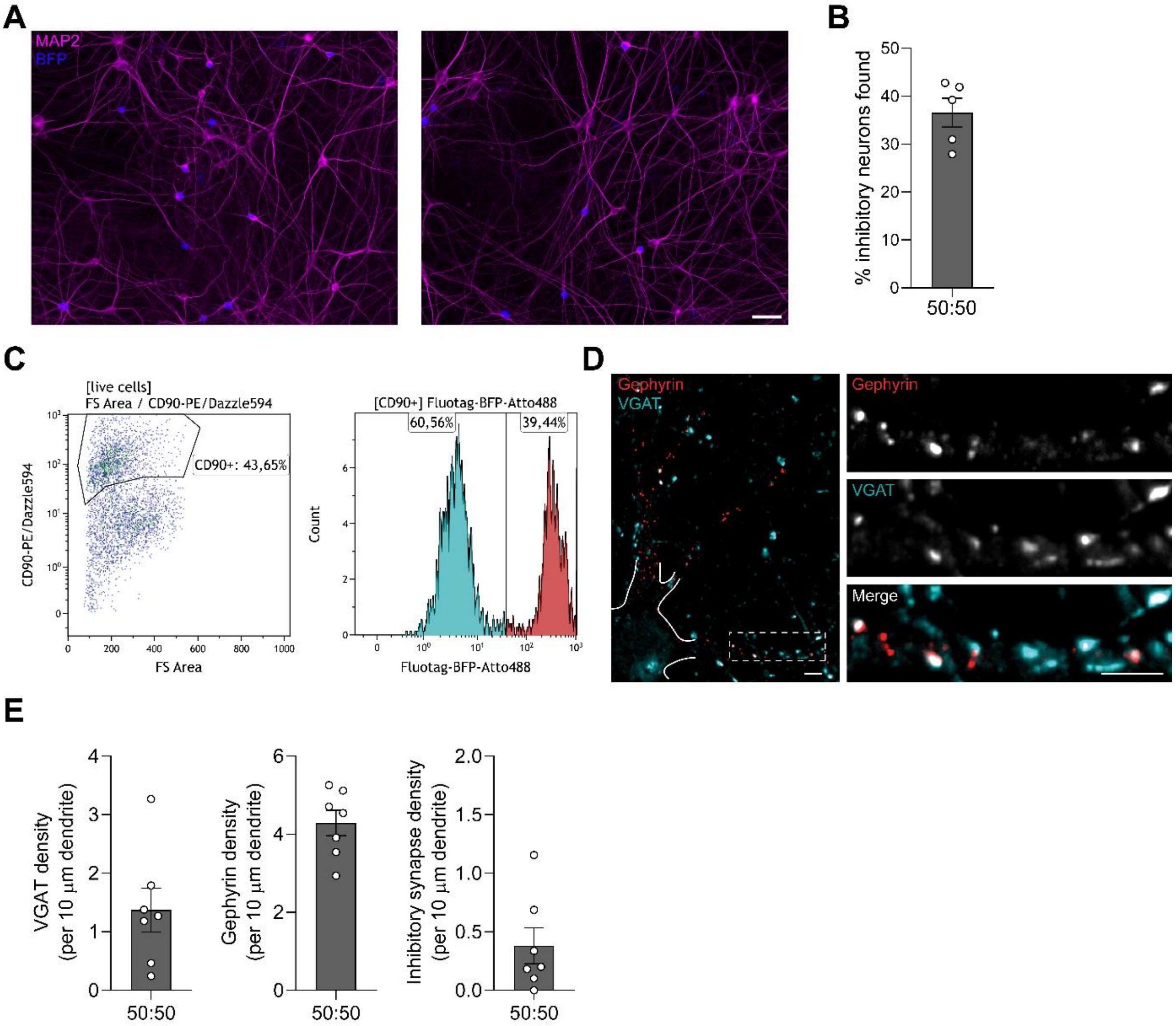

Figure S2 Characterization of glutamatergic/GABAergic co-cultures, related to Figure 4. (**A**) Representative images of 50:50 Ctr:Ctr glutamatergic/GABAergic co-cultures at days *in vitro* (DIV)49 (scale bar = 50 µm). Neurons are stained for microtubule-associated protein 2 (MAP2, in magenta) and GABAergic neurons are show blue fluorescent protein (BFP, in blue)-positive nuclei. (**B**) Quantification of the percentage of GABAergic neurons (BFP-positive nuclei) in 50:50 Ctr:Ctr glutamatergic/GABAergic co-cultures at DIV49. *n = 5/2*. Data represent means ± standard error of the mean (SEM), with individual datapoints representing the mean percentage per coverslip. (**C**) Quantification of the percentage of GABAergic neurons in 50:50 Ctr:Ctr glutamatergic/GABAergic co-cultures at DIV49 by flow cytometry. Cells were first gated for $CD90^+$ events to select neurons and exclude astrocytes prior to the gating of neurons positive for BFP (GABAergic neurons). (**D**) Representative image of 50:50 Ctr:Ctr glutamatergic/GABAergic co-cultures stained with Gephyrin and VGAT to label GABAergic synapses (scale bar = 5 µm). (**E**) Quantification of the number of VGAT, Gephyrin and co-localized VGAT/Gephyrin puncta per 10 µm dendritic length. *n = 7.* Data represent means ± SEM, with individual datapoints representing the mean puncta density per neuron.

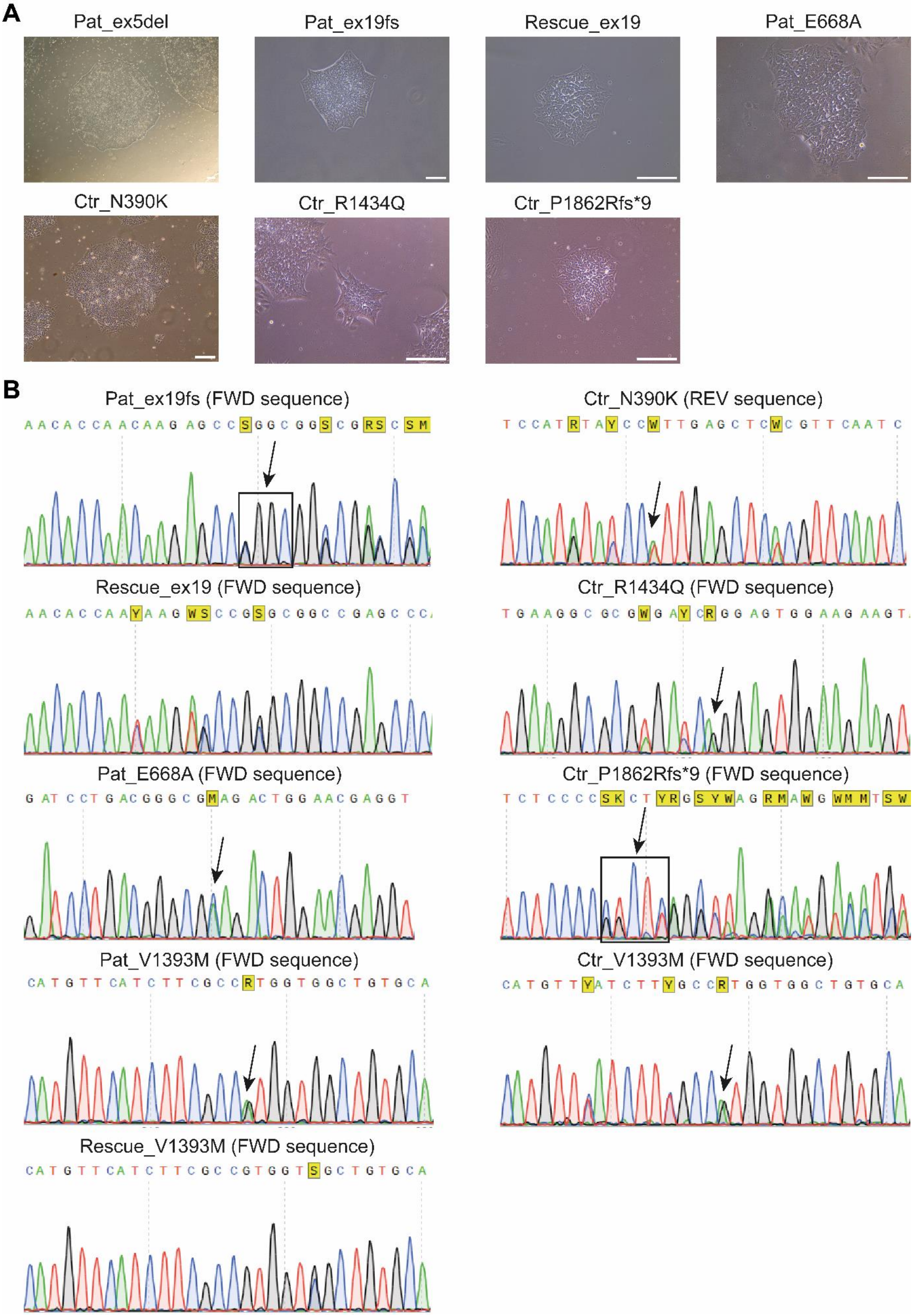
A
Pat_ex5del
Pat_ex19fs
Rescue_ex19
Pat_E668A
Ctr_N390K
Ctr_R1434Q
Ctr_P1862Rfs*9
B
Pat_ex19fs (FWD sequence)
Ctr_N390K (REV sequence)
Rescue_ex19 (FWD sequence)
Ctr_R1434Q (FWD sequence)
Pat_E668A (FWD sequence)
Ctr_P1862Rfs*9 (FWD sequence)
Pat_V1393M (FWD sequence)
Ctr_V1393M (FWD sequence)
Rescue_V1393M (FWD sequence)

Figure S3 Characterization of the included cell lines in this study, related to Figure 1 and 5. (**A**) Induced pluripotent stem cell (iPSC) colonies all showed characteristic morphology. Scale bar = 200 µm. (**B**) Sanger sequences of cell lines used in this study. Arrows indicate the patient or CRISPR-induced variants. In CRISPR lines, silent variants were introduced to disturb guide RNA recognition. Above each sequence, it is indicated whether the forward (FWD) or reverse (REV) sequence is shown.

Supplemental Tables

Table S1 Top 10 parameters contributing to each PC related to Figure 2.

PC	Top 10 Parameter Rescues	PC	Top 10 Parameter Patients
PC1	Burst_Percentage_Avg	PC1	Firing_Rate_in_NB_Avg
	Random_Spikes_Percentage_Avg		total_spikes_in_all_NBs_d_nNB
	Mean_ISI_within_Burst_Avg_sec		Spike_Frequency_per_Electrode
	Number_of_Bursting_Electrodes		Mean_ISI_within_Burst_Avg_sec
	Number_of_spikes		Mean_ISI_within_Burst_Std_sec
	Mean_Firing_Rate_Hz		Number_of_Spikes_per_Burst_Avg
	Spike_Frequency_per_Electrode		Burst_Percentage_Avg
	Firing_Rate_in_NB_Avg		Random_Spikes_Percentage_Avg
	Number_of_Spikes_per_Burst_Avg		Burst_Percentage_Std
	total_spikes_in_all_NBs_d_nNB		hf_Spike_Frequency_per_Electrode
PC2	total_spikes_in_all_hf_NBs_d_nNB	PC2	hf_Network_Burst_Duration_Avg_sec
	hf_Network_Burst_Frequency		Inter_Burst_Interval_Avg_sec
	Number_of_hf_Network_Bursts		Number_of_hf_Network_Bursts
	hf_Network_Burst_Duration_Avg_sec		hf_Network_Burst_Frequency
	hf_Firing_Rate_in_NB_Avg		Network_Burst_Duration_Std_sec
	hf_Spike_Frequency_per_Electrode		total_spikes_in_all_hf_NBs_d_nNB
	hf_Network_Burst_Percentage		Burst_Frequency_Avg_Hz
	Firing_Rate_in_NB_std		hf_Spike_Frequency_per_Electrode
	hf_Network_Burst_Duration_Std_sec		hf_Firing_Rate_in_NB_Avg
	hf_Firing_Rate_in_NB_std		hf_Network_Burst_Percentage
PC38	hf_Network_Burst_Frequency	PC38	Number_of_hf_Network_Bursts
	Number_of_hf_Network_Bursts		hf_Network_Burst_Frequency
	Random_Spikes_Percentage_Avg		Burst_Percentage_Avg
	Burst_Percentage_Avg		Random_Spikes_Percentage_Avg
	Number_of_Network_Bursts		Network_Burst_Frequency
	Network_Burst_Frequency		Number_of_Network_Bursts
	hf_Spike_Frequency_per_Electrode		hf_Spike_Frequency_per_Electrode
	hf_Firing_Rate_in_NB_Avg		hf_Firing_Rate_in_NB_Avg
	total_spikes_in_all_hf_NBs_d_nNB		hf_Network_Burst_Percentage
	Firing_Rate_in_NB_Avg		Firing_Rate_in_NB_Avg
PC39	Random_Spikes_Percentage_Avg	PC39	Burst_Percentage_Avg
	Burst_Percentage_Avg		Random_Spikes_Percentage_Avg
	hf_Network_Burst_Frequency		Number_of_hf_Network_Bursts
	Number_of_hf_Network_Bursts		hf_Network_Burst_Frequency
	Number_of_Network_Bursts		Network_Burst_Frequency
	Network_Burst_Frequency		Number_of_Network_Bursts
	Firing_Rate_in_NB_Avg		hf_Spike_Frequency_per_Electrode
	Spike_Frequency_per_Electrode		hf_Firing_Rate_in_NB_Avg
	hf_Firing_Rate_in_NB_Avg		Number_of_Bursts
	hf_Spike_Frequency_per_Electrode		total_spikes_in_all_NBs_d_nNB

Table S2 Top 10 parameters contributing to each PC related to Figure 3.

PC	Top 10 Parameter controls	PC	Top 10 Parameter p.(V1393M)
PC1	Spike_Frequency_per_Electrode	PC1	Mean_Firing_Rate_Hz
	Firing_Rate_in_NB_Avg		Number_of_spikes
	Mean_Firing_Rate_Hz		Burst_Frequency_Avg_Hz
	Number_of_spikes		Number_of_Bursts
	Mean_ISI_within_Burst_Avg_sec		Number_of_Spikes_per_Burst_Avg
	total_spikes_in_all_NBs_d_nNB		Burst_Percentage_Avg
	Number_of_Spikes_per_Burst_Avg		Random_Spikes_Percentage_Avg
	Burst_Percentage_Avg		total_spikes_in_all_NBs_d_nNB
	Random_Spikes_Percentage_Avg		Mean_ISI_within_Burst_Avg_sec
	Burst_Percentage_Std		Inter_Burst_Interval_Avg_sec
PC2	hf_Firing_Rate_in_NB_std	PC2	Network_Burst_Percentage
	Number_of_hf_Network_Bursts		hf_Network_Burst_Percentage
	hf_Network_Burst_Frequency		IBI_Coefficient_of_Variation_Avg
	hf_Spike_Frequency_per_Electrode		hf_Network_Burst_Duration_Avg_sec
	total_spikes_in_all_hf_NBs_d_nNB		Firing_Rate_in_NB_std
	hf_Network_Burst_Percentage		hf_Firing_Rate_in_NB_Avg
	hf_Network_Burst_Duration_Avg_sec		hf_Spike_Frequency_per_Electrode
	hf_Network_Burst_Duration_Std_sec		hf_Firing_Rate_in_NB_std
	hf_Firing_Rate_in_NB_Avg		total_spikes_in_all_hf_NBs_d_nNB
	Firing_Rate_in_NB_std		IBI_Coefficient_of_Variation_Std
PC28	Number_of_spikes	PC37	Number_of_Network_Bursts
	Mean_Firing_Rate_Hz		Network_Burst_Frequency
	Firing_Rate_in_NB_Avg		hf_Network_Burst_Frequency
	Spike_Frequency_per_Electrode		Number_of_hf_Network_Bursts
	Number_of_Bursts		Burst_Percentage_Avg
	total_spikes_in_all_NBs_d_nNB		Random_Spikes_Percentage_Avg
	Number_of_Spikes_per_Burst_Avg		Mean_Firing_Rate_Hz
	NBI_Avg_sec		Number_of_spikes
	Inter_Burst_Interval_Avg_sec		Number_of_Bursts
	Mean_ISI_within_Burst_Avg_sec		Burst_Frequency_Avg_Hz
PC29	Firing_Rate_in_NB_Avg	PC38	Random_Spikes_Percentage_Avg
	Spike_Frequency_per_Electrode		Burst_Percentage_Avg
	Mean_Firing_Rate_Hz		Number_of_Network_Bursts
	Number_of_spikes		Network_Burst_Frequency
	Burst_Frequency_Avg_Hz		Number_of_hf_Network_Bursts
	Number_of_Bursts		hf_Network_Burst_Frequency
	hf_Firing_Rate_in_NB_std		Number_of_spikes
	Number_of_Network_Bursts		Mean_Firing_Rate_Hz
	Number_of_Spikes_per_Burst_Avg		Number_of_Bursts
	Network_Burst_Frequency		Burst_Frequency_Avg_Hz

Table S3 Description of extracted MEA parameters.

Metric type	Parameter	Unit	Description
Single spikes	Number of spikes		Total number of spikes over the duration of the analysis.
	Mean firing rate	Hz	Total number of spikes divided by the duration of the analysis.
Electrode bursts	Number of bursts		Total number of single-electrode bursts over the duration of the analysis. For a well, the total number of electrode bursts across all electrodes in a well is reported.
	Number of bursting electrodes		Total number of electrodes within the well with bursts/minute greater than the burst electrode criterion.
	Burst duration (Avg)	s	Average time from the first spike to last spike in a single-electrode burst. For a well, the average across electrode averages is reported.
	Burst duration (Std)	s	The standard deviation across electrode burst durations.
	Number of spikes per burst (Avg)		Average number of spikes in a single-electrode burst. For an electrode, the average across bursts is reported. For a well, the average across electrode averages is reported.
	Number of spikes per burst (Std)		The standard deviation across electrode numbers of spikes per burst.
	Mean ISI within burst (Avg)	s	Mean inter-spike interval, time between spikes, for spikes in a single-electrode burst. For a well, the average across electrode averages is reported.
	Mean ISI within burst (Std)	s	The standard deviation across electrode mean ISIs within burst
	Inter-burst interval (Avg)	s	Average time between the start of single-electrode bursts. For a well, the average across electrode averages is reported.
	Inter-burst interval (Std)	s	The standard deviation across electrode IBIs
	IBI Coefficient of Variation (Avg)		The coefficient of variation (standard deviation/mean) of the inter-burst interval, the time between single-electrode bursts. This is a measure of single-electrode burst regularity. For a well, the average across electrode IBI CoVs is reported.
	IBI Coefficient of Variation (Std)		The standard deviation across electrode IBI CoVs
	Burst frequency (Avg)	Hz	Total number of single-electrode bursts divided by the duration of the analysis, in Hz. For a well, the average across electrode burst frequencies is reported.
	Burst frequency (Std)	Hz	The standard deviation across electrode burst frequencies.
	Burst percentage (Avg)	%	The number of spikes in single-electrode bursts divided by the total number of spikes, multiplied by 100. For a well, the average across electrode burst percentages is reported.
	Burst percentage (Std)	%	The standard deviation across electrode burst percentages.
	Random spikes percentage(Avg)	%	The number of spikes not in single-electrode bursts divided by the total number of spikes, multiplied by 100.
Network bursts	Number of network bursts		Total number of network bursts over the duration of the analysis.
	Network burst frequency	Hz	Total number of network bursts divided by the duration of the analysis.

	Spike frequency per electrode	Hz	Total number of spikes detected in all network bursts divided by the durations of all network bursts. This represents the average firing activity within a network burst.
	Total spikes in all NBs d nNB		Total number of spikes detected in all network bursts divided by the number of network bursts. This represents the average number of spikes per network burst.
	Network burst percentage	%	The number of spikes in network bursts divided by the total number of spikes, multiplied by 100.
	Network burst duration (Avg)	s	Average time from the first spike to last spike in a network burst.
	Network burst duration (Std)	s	The standard deviation across all network burst durations.
	Firing rate in NB (Avg)	Hz	Average number of spikes occurring within a network burst divided by the duration of that network burst. This represents the average firing activity within a network burst.
	Firing rate in NB (Std)	Hz	The standard deviation across firing rates across network bursts. This represents the variability in the firing rate within network bursts.
	Network inter-burst interval (Avg)	s	Average time interval between consecutive network bursts.
	Network inter-burst interval (Std)	s	The standard deviation across all network inter-burst intervals. This represents the variability in time intervals between consecutive network bursts.
Fragments / network bursts	Number of hf network bursts		Total number of high frequency network bursts over the duration of the analysis.
	hf Network burst frequency	Hz	Total number of high frequency network bursts divided by the duration of the analysis.
	hf Spike frequency per electrode	Hz	Total number of spikes detected in all high frequency network bursts divided by the durations of all high frequency network bursts. This represents the average firing activity within a high frequency network burst.
	Total spikes in all hf NBs d nNB		Total number of spikes detected in all high frequency network bursts divided by the number of high frequency network bursts. This represents the average number of spikes per high frequency network burst.
	hf Network burst percentage	%	The number of spikes in high frequency network bursts divided by the total number of spikes, multiplied by 100.
	hf Network burst duration (Avg)	s	Average time from the first spike to last spike in a high frequency network burst.
	hf Network burst duration (Std)	s	The standard deviation across all high frequency network burst durations.
	hf Firing rate in NB (Avg)	Hz	Average number of spikes occurring within a high frequency network burst divided by the duration of that high frequency network burst. This represents the average firing activity within a high frequency network burst.
	hf Firing rate in NB (Std)	Hz	The standard deviation across firing rates across high frequency network bursts. This represents the variability in the firing rate within high frequency network bursts.

Table S5 CRISPR/Cas9 strategies and sequences used in this study, related to Figure 1 and 5.

Generated clones	Plasmid-based or RNP complex-based protocol	Forward sequence (5'-3')	Reverse sequence (5'-3')	HDR template (5'-3')	*CACNA1A* variant
Rescue_Ex19	RNP complex-based	**AACACCAACAAGAGCCCGGC**	N/A	C*G*GGCCCGATCGTGGTAGCGGGCCTGTTTCCTGAGGAAGTCCTCGGCGCGCTGCTGGCCGAGGCGCTGGTCCACGGTGGGCTCGGCCGCGCGGGACTTATTGGTGTTGTTGTTGCGGTTCTCCTG*C*G	c.2518_2519 insCGGC corrected
Rescue_V1393M	RNP complex-based	**CATGTTCATCTTCGCCATGG**	N/A	A*A*AGTGGAGACCCACCGACAATCTTTCTCAAACTCTTTGGACTCGTCAGTGCAGTGGAAGAATTTCCCCTTGAAGAGCTGCACAGCGACCACGGCGAAGATGAACATGAATAGCATGTAGACGAT*G*A	c.4177G>A corrected
Ctr_V1393M	RNP complex-based	**ATTCATGTTCATCTTCGCCG**	N/A	G*T*GGAGACCCACCGACAATCTTTCTCAAACTCTTTGGACTCGTCAGTGCAGTGGAAGAATTTCCCCTTGAAGAGCTGCACAGCCACCATGGCAAAGATAAACATGAATAGCATGTAGACGATGAG*G*A	c.4177G>A
Ctr_N390K	Plasmid-based	ccacg**ACGTGAGCTCAATGGGTACA**	aaac**TGTACCCATTGAGCTCACGT**c	C*A*TGACTCTCTTTGTACTCCGTGGCCTGGGATCTCCATCCCTGGGCCCCAGGATGAAAGGGCCTCACCTGCTTTTGAGATCCACTCCATATATCCTTTGAGCTCTCGTTCAATCTGTTGTTGCCG*C*C	c.1170T>A
Ctr_R1434Q	RNP complex-based	**TGAGGTGAAGGCGCGAGACC**	N/A	G*G*CCAGCCTTCTCCCGTGGACACGGTGAAGAGGGTCAGCAGAGCCCACAGCACATTGTCGTAATGGAATTCATACTTCTTCCACTCCTGATCACGCGCCTTCACCTCATTCTTCTCGTAGAGGAG*G*T	c.4301G>A
Ctr_P1862Rfs*9	Plasmid-based	caccc**GTTTCTTGCCTAAGCCGAGAG**	aaac**CTCTCGGCTTAGGCAAGAAAC**	N/A	c.5585_5589 del

Coding variants are listed in NM_001127221.1. Abbreviations: HDR, homology directed repair; N/A, not applicable; RNP, ribonucleoprotein

Table S6 PCR primers used in this study.

PCR primer target	Forward (5'-3')	Reverse (5'-3')
CACNA1A exon 8	GGCACAGTTGTCGGGAAATG	AACGGAGGTACAGGGATGTG
CACNA1A exon 19	TCCATCCAAGCTACAGTGCC	ATGACGTCGATGCTCCCC
CACNA1A exon 26	GTGTTGGGCTCCTGTATCAC	GGGAATGTGCTGGAAAGTGG
CACNA1A exon 27	ACCACTCTTCCTTTCCCTCC	AGCTCTCAGGCCCTTTATCC
CACNA1A exon 37	CGACTGACATCCTACACCCC	AAGAACCCCAAGCCACACTC

Supplemental Methods

Patient information and human iPSC line generation

Pat_ex5del originated from a 47-year old male, who presented symptoms of episodic ataxia type 2, with a heterozygous deletion (NM_001127221.1: c.(631_632)_(784_785)del) in exon 5 of *CACNA1A*. This line was reprogrammed from peripheral blood mononuclear cells (PBMCs) by episomal vectors expressing SOX2, KLF4, MYC, LIN28 and OCT3/4 by the LUMC human iPSC Hotel (https://www.lumc.nl/research/facilities/hipsc-core-facility/).

Pat_ex19fs originated from a 67-year old female who presented with late-onset, chronic ataxia and dystonia, carrying a heterozygous frameshift variant (NM_001127221.1: c.2518_2519insCGGC) in exon 19 of *CACNA1A*. Pat_E668A originated from a 27-year old male who presented a mild, barely progressive ataxia and migraine, and carried a heterozygous missense variant (NM_001127221.1: c.2003A>C) in exon 16 of *CACNA1A*. Both these iPSC lines were reprogrammed from PBMCs with episomal vectors expressing SOX2, KLF4, MYC, LIN28 and OCT3/4 by the Radboudumc Stem Cell Technology Center (SCTC) (https://www.radboudumc.nl/en/research/radboud-technology-centers/stem-cells).

Pat_V1393M originated from a 6-year old female with congenital chronic ataxia and early-onset epileptic encephalopathy with refractory seizures. She carried a heterozygous missense variant (NM_001127221.1: c.4177G>A) in exon 26 of *CACNA1A*. This cell line was reprogrammed from erythroblasts using Sendai virus using the Cytotune reprogramming kit and was made available through the CACNA1A Foundation and the COMBINEDBrain Biorepository. The control cell line, Ctr, (UCSFi001-A, obtained from the Coriell Institute (GM25256, RRID: CVCL_Y803)) was reprogrammed from skin fibroblasts of a 30-year old healthy male.

CRISPR/Cas9 editing of *CACNA1A*

Generation of isogenic cell lines was performed with two different CRISPR/Cas9 strategies: plasmid-based and ribonucleoprotein (RNP) complex-based. Ctr_N390K and Ctr_P1862Pfs*9 iPSCs were generated with the plasmid-based protocol, whereas Rescue_ex19, Rescue_V1393M, Ctr_V1393M and Ctr_R1434Q iPSCs were generated with the RNP complex-based protocol (Table S5). The single guide (sg)RNAs and HDR templates were designed with Benchling (https://www.benchling.com/) (Table S5).

For the plasmid-based protocol, as previously described (Hommersom, et al. 2022), sgRNAs were cloned into the pSpCas9(BB)-2A-Puro (PX459) V2.0 plasmid (a gift from Feng Zhang (RRID:Addgene_62988)) (Ran, et al. 2013). Nucleofection of 800,000 cells with 5 µg plasmid, and if applicable, 4 µM HDR template (Ultramer DNA oligo, IDT) was performed with the 4D-Nucleofector™ System (program CA-137, Lonza). Cells were seeded into a recombinant human laminin LN521 (5 µg/mL; BioLamina, #LN521)-coated 6-well plate in Essential 8™ Flex Basal Medium (Gibco,

#A2858501) supplemented with primocin (0.1 µg/ml; Invivogen, #ant-pm-2) and Revitacell (Gibco, #A2644501). 24 h after nucleofection, the cells were selected with puromycin (0.5 µg/ml; Sigma-Aldrich, #P9620) for 24 h. Surviving colonies were picked and individually transferred into a 96-well plate.

For the RNP complex-based protocol, sgRNAs were ordered as Alt-R crRNAs (IDT), which were annealed to Alt-R tracrRNAs (IDT, #1072533) in nuclease-free duplex buffer (IDT, #11-01-03-01) at 95 °C for 5 min. The resultant sgRNAs were incubated with Alt-R S.p. Cas9 Nuclease V3 (IDT, #1081059) for 20 min at room temperature. Nucleofection of 200,000 or 300,000 cells with 4 µM RNP complex and 4 µM HDR template was performed with the 4D-Nucleofector™ System (program CA-137, Lonza). Cells were seeded into a recombinant human laminin 521-coated 12- or 24-well plate in the presence of Revitacell. 96 h after nucleofection, half of the cells were plated as single cells onto a 6-well plate and half was taken for DNA isolation and Sanger sequencing to check for HDR efficiency. Colonies derived from the single cells were picked and individually transferred into a 96-well plate.

To identify edited clones, DNA was isolated using Proteinase K (1 mg/mL, Thermo Scientific) and amplified by PCR (Table S6). PCR products were purified with Exonuclease I (1.5 U/µL, Thermo Scientific) and FastAP Thermosensitive Alkaline Phosphatase (0.3 U/µL, Thermo Scientific), and sequenced by Sanger sequencing (Figure S3).

Characterization of iPSC lines

Morphology of the induced pluripotent stem cell (iPSC) lines was assessed by bright-field microscopy (Figure S3). Images were taken with an Invitrogen™ EVOS™ XL Core cell imaging system with 4x, 10x and 20x EVOS objectives. DNA isolation for rtTA-*Ngn2*-positive or rtTA-*Ascl1/Dlx2*-positive iPSCs (see below) was done via the QIAamp DNA Mini Kit (Qiagen, #51306). For Rescue_ex19, Ctr_N390K, Ctr_R1434Q, and Ctr_P1862Pfs*9 iPSC lines, off-target analysis was performed by sequencing the top 3 off-target sites of each sgRNA predicted by both Benchling and CRISPOR.(Concordet and Haeussler 2018) These iPSC lines, as well as Pat_ex5del, Pat_ex19fs and Pat_E668A lines were validated for pluripotency markers and trilineage differentiation potential (STEMdiff™ Trilineage Differentiation Kit, STEMCELL Technologies, #05230) by immunocytochemistry after all experiments were performed, within 12 passages after thawing the cell lines. Short tandem repeat (STR) analysis of 16 loci was performed using the AmpFLSTR identifiler PCR amplification kit (Life Technologies). Lastly, copy number variation (CNV)/whole exome sequencing (WES) analysis was carried out for all these cell lines to check for major karyotype abnormalities, after all experiments were performed to ensure genomic integrity throughout the experiment. CNV/WES revealed a 60-65% trisomy 8 in rtTA-*Ngn2*-positive Pat_E668A cells and a chr.7p gain in rtTA-*Ngn2*-positive Pat_ex5del cells. For Pat_V1393M, Rescue_V1393M, and Ctr_V1393M rtTA-*Ngn2*-positive or rtTA-*Ascl1/Dlx2*-positive iPSCs, genomic stability was assessed before experimentation by detection of recurrent genetic abnormalities using the iCS-digital™ PSC test, provided as a service by Stem Genomics (https://www.stemgenomics.com/), which identified a chr.12p gain in the rtTA-*Ngn2*-positive Pat_V1393M cell line. The absence of mycoplasma was tested regularly by the MycoAlert™ PLUS mycoplasma detection kit (Lonza, # LT07-710). All data is available upon request.

Generation of rtTA-*Ngn2*-positive iPSCs

All cell lines in this study were transduced with lentiviral vectors to integrate rtTA (pLV-EF1α>Tet3G:IRES:Neo) and *Ngn2* (pLV[TetOn]-Puro-TRE3G>mNeurog2([NM_009718.3])) transgenes into their genome. 48 h after transduction with both vectors, cells were selected with G418 (25 µg/ml; Sigma- Aldrich, #G8168) and puromycin (0.5 µg/ml), of which concentrations increased over time. Colonies that survived the selection process were cultured in Essential 8™ Flex Basal medium, supplemented with primocin, G418 (50 µg/ml) and puromycin (0.5 µg/ml) on Vitronectin-N (VTN-N; Gibco, #A14700) or Geltrex (Gibco, #A1413302)-coated plates at 37°C/5% CO_2. Cells were passaged with ReLeSR (STEMCELL Technologies, #100-0483), 1-2 times per week when they reached 80-90% confluency and cryopreserved using PSC cryomedium (Gibco, #A2644601).

Generation of rtTA-*Ascl1/Dlx2*-positive iPSCs

24-well plates were precoated with recombinant human laminin LN521. Single cells were generated with TrypLE™ Express (Gibco, #12604021) and 50,000 cells were plated in Essential 8™ Basal medium (Gibco, #A1517001), supplemented with primocin and RevitaCell into one well of a 24-well plate. 24 h after plating, iPSCs were refreshed with Essential 8™ Basal medium supplemented with primocin, and transfected with a piggybac vector PB-*Ascl1*-*Dlx2* (van Voorst, et al. 2025) and a transposase using Lipofectamine Stem Transfection Reagent (Invitrogen, #STEM00003) in Opti-MEM (Gibco, #31985062). 24 h after transfection medium was refreshed and selection with puromycin was started (0.5 µg/ml), which was increased over time. The iPSCs that survived the selection process were cultured in Essential 8™ Flex Basal medium, supplemented with primocin, and treated with puromycin (0.5 µg/ml) for 24 h during every second or third split.

Neuronal differentiation

Human iPSCs were differentiated into glutamatergic neurons by doxycycline-inducible *Ngn2* overexpression (Zhang, et al. 2013, Frega, et al. 2017) or into GABAergic cortical neurons by doxycycline-inducible *Ascl1* and *Dlx2* overexpression (Yang, et al. 2017, van Voorst, et al. 2025). After at least 2 and maximal 10 passages after thawing, single cells [days *in vitro* (DIV0)] were generated from rtTA-*Ngn2*-positive or rtTA-*Ascl1/Dlx2*-positive iPSCs for neuronal differentiation by incubating with TrypLE™ Express at 37°C/5% CO_2. The iPSCs were resuspended in Essential 8™ Basal medium, supplemented with RevitaCell, primocin and doxycycline (4 µg/mL; Sigma Aldrich, #D9891). The plates were pre-coated with poly-L-ornithine hydrobromide (50 µg/mL; Sigma-Aldrich, #P3655) in borate buffer (50 mM) for 3 h at 37°C/5% CO_2, followed by overnight incubation with human recombinant laminin LN521 at 4°C. At DIV1, culture medium was changed to DMEM/F12 medium (Gibco, #11320074), supplemented with MEM non-essential amino acid solution (Sigma-Aldrich, #M7145), N2 (Gibco, #17502048), recombinant human BDNF (10 ng/mL; PromoCell, #C-6621), NT3 (10 ng/mL; PromoCell, #C-66425), doxycycline and mouse laminin from Engelbreth-Holm-Swarm sarcoma (0.2 µg/mL; Sigma-Aldrich, #L2020). To support neuronal maturation and viability, rat embryonic astrocytes were added to the neuronal culture in a 1:1 ratio/well at DIV2. At DIV3, the medium was changed to Neurobasal medium (Gibco, #21103049) supplemented with B-27 (20 µg/mL; Gibco, #17504001), primocin, GlutaMAX (10 µg/mL; Gibco, #35050038), BDNF, NT3 and doxycycline. Furthermore, cytosine β-D-arabinofuranoside hydrochloride (Ara-C) (2 µM; Sigma-Aldrich, #C6645) was added once at DIV3, to remove any proliferating cells from the culture. From DIV6 to DIV9, half of the medium was refreshed every other day with fresh neurobasal medium supplemented with B-27, primocin, GlutaMAX, BDNF, NT3 and doxycycline. From DIV10 onwards till the end of the neuronal culture, every other day half of the medium was refreshed with neurobasal medium supplemented with B-27, primocin, GlutaMAX, BDNF, NT3 and additional 2.5% fetal bovine serum (FBS; Sigma-Aldrich, #F7524), to support astrocyte viability. Throughout the entire differentiation process, the cultures were incubated at 37°C/5% CO_2.

Single-cell electrophysiology

Coverslips with DIV42 neurons were placed in a recording chamber on the stage of an Olympus BX51WI upright microscope (Olympus Life Science), equipped with infrared differential interference contrast optics, an Olympus LUMPlanFL N 60x water-immersion objective (Olympus Life Science), and a kappa MXC 200 camera system (Kappa optronics GmbH) for visualization. The recording chamber was continuously perfused with oxygenated (95% O_2/5% CO_2) artificial cerebrospinal fluid (aCSF) at 32°C containing (in mM): 124 NaCl, 1.25 NaH_2PO_4, 3 KCl, 26 $NaHCO_3$, 11 Glucose, 2 $CaCl_2$, 1 $MgCl_2$. Patch pipettes (ID 0.86 mm, OD1.05 mm, resistance 6–8 MΩ) were pulled from borosilicate glass with filament and fire-polished ends (Science Products GmbH) using the Narishige PC-10 micropipette puller. These pipettes were filled with a potassium-based intracellular solution containing (in mM): 130 K-Gluconate, 5 KCl, 10 HEPES, 2.5 $MgCl_2$, 4 Na_2-ATP, 0.4 Na_3-GTP, 10 Na-phosphocreatine, 0.6 EGTA (with pH adjusted to 7.25 and osmolarity to 290 mOsmol). We acquired

recordings using a Digidata 1140A digitizer and a Multiclamp 700B amplifier (Molecular Devices), with a sampling rate set at 20 kHz and a lowpass 1kHz filter during recording. We did not correct for liquid junction potential. Recordings were omitted from analysis if series resistance was above 25 MΩ or when the recording reached below a 10:1 ratio of membrane resistance to series resistance. Analysis of passive membrane properties was conducted in voltage clamp mode at a holding potential of -60 mV. Resting membrane potential (RMP), was determined in current clamp mode directly after reaching whole-cell configuration. Consequently, active intrinsic properties were measured with a stepwise current injection protocol ranging from -30 pA to +70 pA. Intrinsic properties were analysed with the Action Potential Search algorithm of Clampfit 11.2 (Molecular devices). We assessed the properties of every first elicited action potential. Where applicable, measurements were taken relative to the threshold of each action potential. Rise and decay time were measured between 10 and 90% of the threshold-relative amplitude.

Immunocytochemistry and image analysis

Cells were fixed in 4% paraformaldehyde (PFA) containing 4% sucrose for 10 minutes at room temperature (RT), followed by three 5-minute washes in PBS. For permeabilization and blocking, cells were incubated in PBS supplemented with 10% normal goat serum (NGS) and 0.2% Triton X-100 for 30 minutes at RT. Primary antibodies were diluted in PBS containing 5% NGS and 0.1% Triton X-100 and applied overnight at 4 °C. The following primary antibodies were used: guinea pig anti-MAP2 (1:1000; Synaptic Systems, #188004) for quantification of GABAergic neurons, and guinea anti-VGAT (1:200; Synaptic Systems, #131308) and chicken anti-Gephyrin (1:500; Synaptic Systems, #147009) for GABAergic synapse quantification. After primary incubation, cells were washed three times for 5 minutes with PBS and incubated for 1 hour at RT with secondary antibodies (1:1000) and FluoTag-X2 anti-TagFP Atto 488 (1:200; NanoTag Biotechnologies, #N0502-At488-L) for the quantification of GABAergic neurons, diluted in PBS containing 5% NGS and 0.1% Triton X-100. Cells were then washed three times for 5 minutes with PBS. Coverslips were briefly rinsed in ultrapure water and mounted in DAKO mounting medium.

The percentage of GABAergic neurons was determined from images acquired using a Zeiss Axio Imager Z1 microscope. The proportion of TagFP-positive neurons was calculated relative to the total number of MAP2-positive neurons within each field of view, and averaged across images for each coverslip. These mean values were subsequently used for data visualization. For GABAergic synapse quantification, images were acquired using the same microscope. Regions of interest (ROIs) were manually traced along the proximal dendrites of neurons in ImageJ. Synaptic puncta were identified and quantified using the SynBot plugin (Savage, et al. 2024) with the Ilastik-based thresholding method to segment presynaptic VGAT and postsynaptic Gephyrin puncta. Co-localization analysis was then performed to identify putative functional synapses. The number of functional synaptic puncta within each dendritic ROI was quantified using a custom ImageJ macro, normalized to 10 μm of dendrite length, and averaged across dendrites for each neuron. These mean values were subsequently used for data visualization.

Flow cytometry and analysis

GABAergic and glutamatergic iPSCs were plated at a 1:1 ratio on 6-well plates, which were then co-cultured with rodent astrocytes at a neurons:astrocyte ratio of 1:1 under the same culture conditions as other experiments. At DIV49, cells were dissociated using Accutase (Sigma, #A6964) and collected into tubes. Cells were resuspended either in PBS (unstained control) or in PBS containing LIVE/DEAD™ Fixable Near-IR Viability Dye (780) (1:4000; Thermo Fisher, #L34994) and incubated for 30 min at 4 °C protected from light. Cells were washed with flow buffer (PBS supplemented with 1% BSA) and fixed with the Fix solution from the Fix&Perm kit (Nordic-MuBio) for 15 min at room temperature (RT). Fixed cells were washed with flow buffer. Unstained controls were stored at 4 °C until use, while LIVE/DEAD-stained cells were incubated in permeabilization solution (Fix&Perm kit,

Nordic-MuBio) containing CD90 (Thy-1) PE/Dazzle 594 (1:50; BioLegend, #328134) and FluoTag-X2 anti-TagFP Atto 488 (1:200; NanoTag Biotechnologies, #N0502-At488-L). Samples were incubated for 30 min at 4 °C in the dark, washed twice with flow buffer, and resuspended for acquisition. Data were acquired on a Gallios flow cytometer (Beckman Coulter) using calibrated instrument settings and appropriate compensation controls.

Flow cytometry data were analysed using Kaluza 2.1 software (Beckman Coulter). Cells were first gated based on forward scatter area (FSC-A) and side scatter area (SSC-A) to exclude debris. Doublets were then removed by comparing FSC-A vs FSC-H and SSC-A vs SSC-H parameters. From the single-cell population, viable cells were identified as those with low fluorescence in the LIVE/DEAD channel, as the dye penetrates and labels intracellular amines only in cells with compromised membranes. Within the live single-cell population, CD90-positive events were classified as neurons, as astrocytes were confirmed to be CD90-negative (data not shown). The proportion of BFP-positive (GABAergic) neurons was then quantified within this gated population. Unstained control samples were used to define background autofluorescence in each channel and to set thresholds for positive staining.

Supplemental references

Concordet J-P, Haeussler M. CRISPOR: intuitive guide selection for CRISPR/Cas9 genome editing experiments and screens. *Nucleic Acids Research*. 2018;46(W1):W242-W245. doi:10.1093/nar/gky354

Frega M, van Gestel SH, Linda K, van der Raadt J, Keller J, Van Rhijn JR, Schubert D, Albers CA, Nadif Kasri N. Rapid Neuronal Differentiation of Induced Pluripotent Stem Cells for Measuring Network Activity on Micro-electrode Arrays. *J Vis Exp*. Jan 8 2017;(119)doi:10.3791/54900

Hommersom MP, Bijnagte-Schoenmaker C, Albert S, van de Warrenburg BPC, Nadif Kasri N, van Bokhoven H. Generation of induced pluripotent stem cell lines carrying monoallelic (UCSFi001-A-60) or biallelic (UCSFi001-A-61; UCSFi001-A-62) frameshift variants in CACNA1A using CRISPR/Cas9. *Stem Cell Res*. May 2022;61:102730. doi:10.1016/j.scr.2022.102730

Ran FA, Hsu PD, Wright J, Agarwala V, Scott DA, Zhang F. Genome engineering using the CRISPR-Cas9 system. *Nat Protoc*. Nov 2013;8(11):2281-2308. doi:10.1038/nprot.2013.143

Savage JT, Ramirez J, Risher WC, Wang Y, Irala D, Eroglu C. SynBot: An open-source image analysis software for automated quantification of synapses. *bioRxiv*. 2024:2023.06.26.546578. doi:10.1101/2023.06.26.546578

van Voorst TW, van Boven MA, Marinus KI, Colón-Mercado JM, Schretzmeir J, Haag C, Toonen RF, Koopmans F, Ward ME, Smit AB*, et al*. One-step induction of human GABAergic neurons promotes presynaptic development & synapse maturation. *bioRxiv*. 2025:2025.06.30.662293. doi:10.1101/2025.06.30.662293

Yang N, Chanda S, Marro S, Ng Y-H, Janas JA, Haag D, Ang CE, Tang Y, Flores Q, Mall M*, et al*. Generation of pure GABAergic neurons by transcription factor programming. *Nature Methods*. 2017/06/01 2017;14(6):621-628. doi:10.1038/nmeth.4291

Zhang Y, Pak C, Han Y, Ahlenius H, Zhang Z, Chanda S, Marro S, Patzke C, Acuna C, Covy J*, et al*. Rapid single-step induction of functional neurons from human pluripotent stem cells. *Neuron*. Jun 5 2013;78(5):785-98. doi:10.1016/j.neuron.2013.05.029
